# Integrated Analysis of Copy Number Variations and Gene Expression Profiling in Hepatocellular carcinoma

**DOI:** 10.1038/s41598-017-11029-y

**Published:** 2017-09-05

**Authors:** Chenhao Zhou, Wentao Zhang, Wanyong Chen, Yirui Yin, Manar Atyah, Shuang Liu, Lei Guo, Yi Shi, Qinghai Ye, Qiongzhu Dong, Ning Ren

**Affiliations:** 1 Department of Liver Surgery, Liver Cancer Institute, Zhongshan Hospital, Fudan University, Shanghai, China; 2 Institute of Fudan-Minhang Academic Health System, Minhang Hospital, Zhongshan Hospital, Fudan University, Shanghai, China; 3 Key Laboratory of Carcinogenesis and Cancer Invasion, Ministry of Education, Shanghai, China; 4 Biomedical Research Centre, Zhongshan Hospital, Fudan University, Shanghai, China; 5 Institutes of Biomedical Sciences, Fudan University, Shanghai, China

## Abstract

Hepatocellular carcinoma (HCC) is one of the top three cancer killers worldwide. To identify CNV-driven differentially expressed genes (DEGs) in HBV related HCC, this study integrated analysis of copy number variations (CNVs) and gene expression profiling. Significant genes in regions of CNVs were overlapped with those obtained from the expression profiling. 93 CNV-driven genes exhibiting increased expression in the duplicated regions and 45 showing decreased expression in the deleted regions were obtained, which duplications and deletions were mainly documented at chromosome 1 and 4. Functional and pathway enrichment analyses were performed using DAVID and KOBAS, respectively. They were mainly enriched in metabolic process and cell cycle. Protein-protein interaction (PPI) network was constructed by Cytoscape, then four hub genes were identified. Following, survival analyses indicated that only high NPM1 expression was significantly and independently associated with worse survival and increased recurrence in HCC patients. Moreover, this correlation remained significant in patients with early stage of HCC. In addition, we showed that NPM1 was overexpressed in HCC cells and in HCC versus adjacent non-tumor tissues. In conclusion, these results showed that integrated analysis of genomic and expression profiling might provide a powerful potential for identifying CNV-driven genes in HBV related HCC pathogenesis.

## Introduction

Hepatocellular carcinoma (HCC) is one of the top three types of fatal cancer in China and the world^1, 2^. In China, the high incidence of HCC is mainly attributed to the prevalence of hepatitis virus infection, especially hepatitis B virus (HBV). Non-alcoholic fatty liver diseases and alcoholic liver diseases are also risk factors to drive the process of developing HCC^3^. However, a lack of knowledge regarding the precise molecular mechanisms underlying HCC progression limits the ability to treat HCC effectively.

Copy number variations (CNVs) are DNA segments, which are larger than 1 kb in length when compared to a reference genome, that can lead to activation of oncogenes and inactivation of tumor suppressor genes in cancers^4, 5^. CNVs can effectively affect gene expression and are related to the susceptibility of diseases. Several studies have shown that a duplication or deletion of CNVs affects the expression of genes and cancer-related biologic processes^6^. Duplications of chromosome 1, 7, 8 and 20 and deletions of chromosome 4, 8, 13 and 17 have been identified in HCC through traditional technical methods. For example, the CNV of chromosome 13q might be used to monitor the progression of chronic hepatitis-associated liver carcinogenesis^7–10^.

Gene expression profiling by microarray analysis has been shown to be a powerful tool for the identification of cancer-related genes. However, a large number of differentially expressed genes (DEGs) can be obtained through the analysis. Hence, the key points of analyzing gene expression profiling are how to accurately select out which DEGs are critical to neoplastic process (“driver genes”) and which are not (“passenger genes”)^11^.

Several studies have been conducted through integrated analysis of CNVs and gene expression profiling in HCC, but they were limited to the use of small sized tumor samples or relatively lower-resolution platforms^12, 13^.

In this study, we applied a whole-genome SNP 6.0 array to analyze CNVs of the 33 paired HBV related HCC and non-tumor tissues. The gene expression profiling data were obtained from our previous studies (GSE14520). By integrating the analysis of CNVs and gene expression profiling to identify CNV-driven DEGs, we may light further insights of HBV related HCC development at a molecular level, and explore a clinically useful candidate gene for diagnosis, prognosis, and drug targets.

## Results

### Identification of significant CNVs in HCC genomes

**Figure 1:**
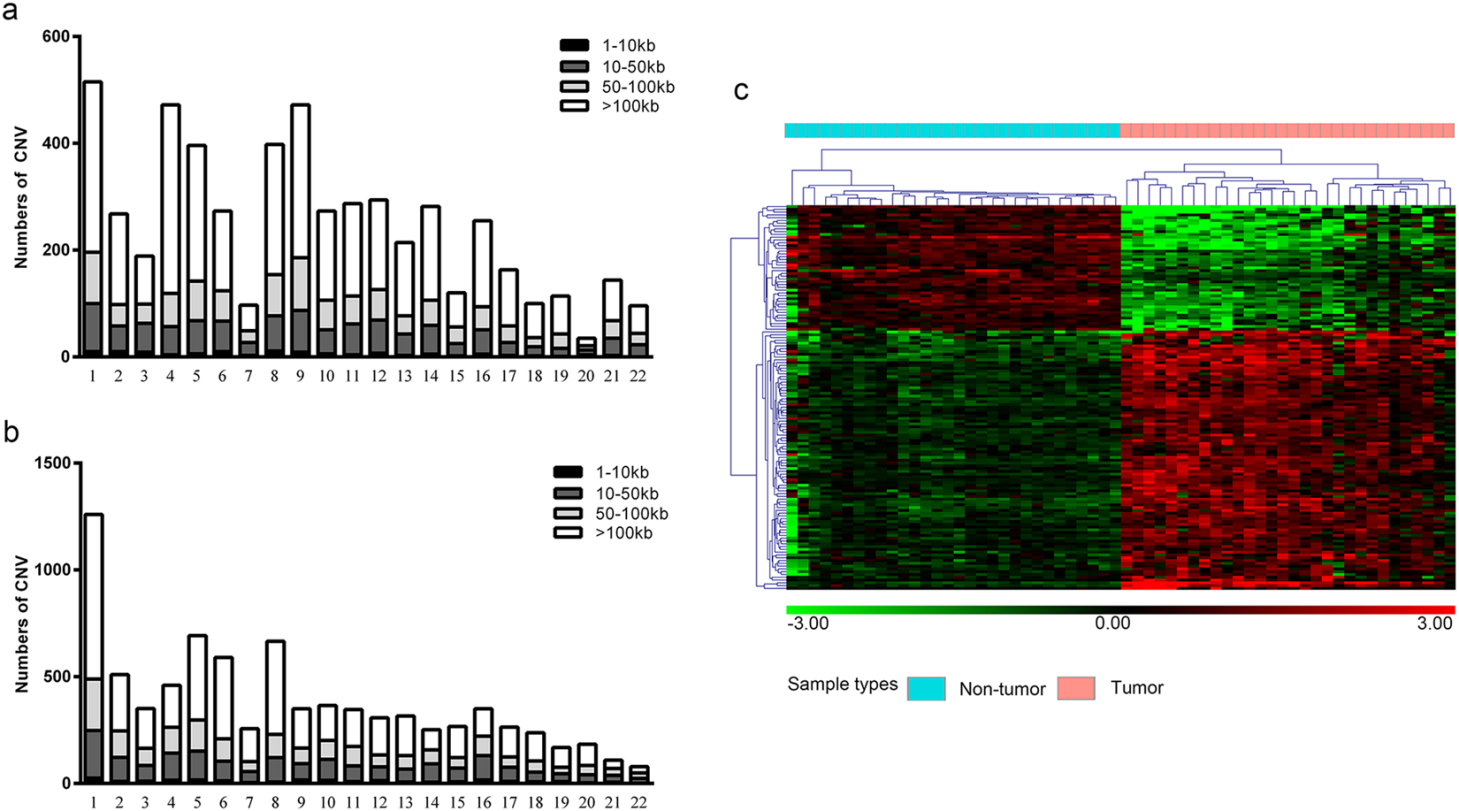
(**a**) Distributions of copy number deletions in the chromosomes, (**b**) Distributions of copy number duplications in the chromosomes. (**c**) Hierarchical clustering of gene expression profiling. Samples are indicated along the horizontal axis and grouped by the color bar above the heat map. Blue represents non-tumor tissue and red represents tumor tissue.

We analyzed the hybridization signal intensities of 33 paired HCC and non-tumor tissues to identify regions of CNVs. A total of 13,839 CNVs were identified in the 33 HCC genomes, including 5,457 copy number deletions (mean size, 349.1 kb) and 8,382 copy number duplications (mean size, 419.0 kb) (Supplementary Table 1). CNVs were scattered across chromosome 1 to 22, and both of the highest duplication and deletion were found in chromosome 1. The second highest number of duplications and deletions were documented at chromosome 5 and 4, respectively. HCC genomes had a mean of 419 CNVs, and copy number duplications were more commonly observed than deletions (1.5:1). It was found that regions of >100 kb long had the most copy number deletions and duplications (Fig. 1a,b).

To find potential HCC-related significant CNVs, we further evaluated CNVs using the following standard: the gene of CNVs was present in at least 10% (4 samples) samples. Accordingly, a total of 2,912 significant CNV genes were obtained, including 875 deletions and 2,037 duplications (Supplementary Table 2 and 3).

### Analysis of gene expression profiling

The total number of samples analyzed was 30 paired HCC and non-tumor tissues from our previous study (GSE14520). The baseline characteristics of these patients were similar to the 33 patients whose paired HCC and non-tumor tissues analyses were carried out with a whole-genome SNP 6.0 array (Table 1). All patients of these two cohorts were infected with hepatitis B virus. In total, 965 genes were differentially expressed by at least two-fold with statistical significance (*P* < 0.05). Of these, 389 genes were found to be up-regulated while 576 genes were found to be down-regulated.

**Table 1:** Clinical Characteristics of Patients in two Cohorts at the Time of Surgery.

Clinical variable — no. (%)	Cohort 1 (N = 33)	Cohort 2 (N = 30)	*P* value^a^
Gender			
Male	30(90.9)	25(83.3)	0.462
Female	3(9.1)	5(16.7)	
Age			
≥50 years	17(51.5)	15(50)	1.000
<50 years	16(48.5)	15(50)	
AFP			
>300 ng/mL	12(36.4)	18(60)	0.073
≤300 ng/mL	21(63.6)	12(40)	
ALT			
>50 U/L	12(36.4)	15(50)	0.316
≤50 U/L	21(63.6)	15(50)	
Cirrhosis			
Yes	31(93.9)	28(93.3)	1.000
No	2(6.1)	2(6.7)	
Tumor size			
>5 cm	15(45.5)	14(46.7)	1.000
≤5 cm	18(54.5)	16(53.3)	
Multinodular			
Yes	12(36.4)	6(20)	0.174
No	21(63.6)	24(80)	
BCLC staging			
0-A	21(63.6)	20(66.7)	1.000
B-C	12(36.4)	10(33.3)	
TNM staging			
I	13(39.4)	14(46.7)	0.616
I–III	20(60.6)	16(53.3)	

^a^Fisher’s exact tests; *P* value < 0.05 was considered statistically significant.

Abbreviations: AFP, alpha-fetoprotein; ALT, alanine aminotransferase; BCLC, Barcelona Clinic Liver Cancer; TNM, tumor-nodes-metastases.

### Integrative analysis of CNVs and gene expression profiling to identify CNV-driven DEGs

To identify driver genes in regions of CNVs, we performed an integrated analysis of CNVs and gene expression profiling. A total of 793 genes with only gene expression changes and 2740 genes with CNV changes, but no changes in transcript levels, were observed. Furthermore, 138 CNV-driven genes were identified with the same expressional tendencies, including 93 CNV-driven genes exhibiting increased expression in the duplicated regions and 45 showing decreased expression in the deleted regions (Supplementary Table 4). The highest number of duplications was documented at chromosome 1, while the highest number of deletions was recorded at chromosome 4. Hierarchical clustering of the normalized gene expression values for the 138 CNV-driven genes was generated and visualized via a heat map, where distinctive expression patterns can be observed. The clustered heat map of expression values for 138 DEGs in the regions of CNVs can effectively distinguish tumor and non-tumor samples (Fig. 1c).

### Functional and pathway enrichment analysis of CNV-driven DEGs

To gain further insights into the function of identified CNV-driven DEGs, functional and pathway enrichment analyses were performed using DAVID and KOBAS, respectively.

According to Go analysis results, the down-regulated CNV-driven DEGs were significantly enriched in biological processes (BP), including ethanol oxidation, xenobiotic metabolic process and metabolic process; the up-regulated CNV-driven DEGs were significantly enriched in positive regulation of translation, cell division and DNA damage response (Table 2). On the basis of cellular component (CC), the down-regulated CNV-driven DEGs mostly assembled at the cytosol, extracellular exosome and apical plasma membrane, while up-regulated CNV-driven DEGs assembled at the pathways of membrane, nucleoplasm and cytoplasm (Table 2). In addition, for molecular function (MF), the down-regulated CNV-driven DEGs prominently accumulated in alcohol dehydrogenase activity and serine-type endopeptidase activity, and up-regulated CNV-driven DEGs were enriched in protein binding, poly(A) RNA binding and cadherin binding involved in cell-cell adhesion (Table 2).

**Table 2:** Gene ontology analysis of CNV-driven DEGs associated with HCC.

Expression	Category	Term/gene function	Count	*P* value
Down-regulated	GOTERM_BP_DIRECT	GO:0006069~ethanol oxidation	3	3.97E-04
	GOTERM_BP_DIRECT	GO:0006805~xenobiotic metabolic process	4	9.72E-04
	GOTERM_BP_DIRECT	GO:0006508~proteolysis	6	0.006776
	GOTERM_BP_DIRECT	GO:0008152~metabolic process	4	0.00949
	GOTERM_BP_DIRECT	GO:0051919~positive regulation of fibrinolysis	2	0.009971
	GOTERM_CC_DIRECT	GO:0005829~cytosol	17	0.002497
	GOTERM_CC_DIRECT	GO:0070062~extracellular exosome	13	0.02121
	GOTERM_CC_DIRECT	GO:0016324~apical plasma membrane	4	0.031988
	GOTERM_MF_DIRECT	GO:0004024~alcohol dehydrogenase activity, zinc-dependent	3	9.64E-05
	GOTERM_MF_DIRECT	GO:0004252~serine-type endopeptidase activity	6	3.21E-04
	GOTERM_MF_DIRECT	GO:0016491~oxidoreductase activity	5	0.001768
	GOTERM_MF_DIRECT	GO:0004060~arylamine N-acetyltransferase activity	2	0.007705
	GOTERM_MF_DIRECT	GO:0030492~hemoglobin binding	2	0.01026
Up-regulated	GOTERM_BP_DIRECT	GO:0045727~positive regulation of translation	4	0.003197
	GOTERM_BP_DIRECT	GO:0051301~cell division	7	0.010732
	GOTERM_BP_DIRECT	GO:0042769~DNA damage response, detection of DNA damage	3	0.015911
	GOTERM_BP_DIRECT	GO:0090306~spindle assembly involved in meiosis	2	0.015999
	GOTERM_BP_DIRECT	GO:0006695~cholesterol biosynthetic process	3	0.017632
	GOTERM_CC_DIRECT	GO:0016020~membrane	26	4.42E-05
	GOTERM_CC_DIRECT	GO:0005654~nucleoplasm	29	1.19E-04
	GOTERM_CC_DIRECT	GO:0005737~cytoplasm	43	2.37E-04
	GOTERM_CC_DIRECT	GO:0005829~cytosol	32	2.73E-04
	GOTERM_CC_DIRECT	GO:0000785~chromatin	5	0.001167
	GOTERM_MF_DIRECT	GO:0005515~protein binding	53	1.60E-05
	GOTERM_MF_DIRECT	GO:0044822~poly(A) RNA binding	18	7.10E-05
	GOTERM_MF_DIRECT	GO:0051082~unfolded protein binding	4	0.018488
	GOTERM_MF_DIRECT	GO:0098641~cadherin binding involved in cell-cell adhesion	6	0.018571

If there were more than five terms enriched in this category, top five terms were selected according to *P* value.

Count: the number of enriched genes in each term.

DEGs, differentially expressed genes; GO, gene ontology; CNV, copy number variation.

**Figure 2:**
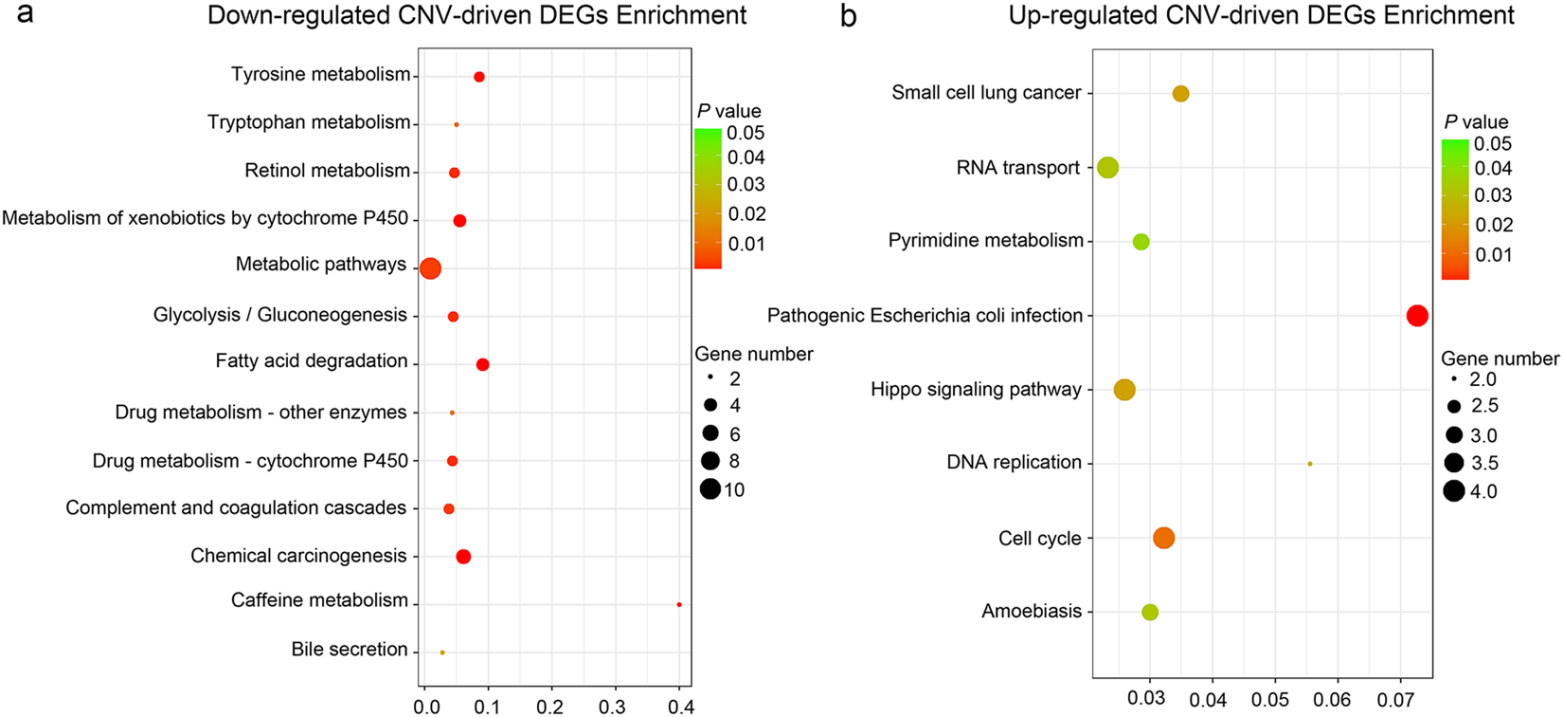
Kyoto Encyclopedia of Genes and Genomes (KEGG) enrichment scatter plot of DEGs. The y-axis represents the name of the pathway, and the x-axis represents the Rich factor. Dot size represents the number of different genes and the color indicates the q-value. (**a**) Down-regulated CNV-driven DEGs Enrichment analysis results. (**b**) Up-regulated CNV-driven DEGs Enrichment analysis results.

As for KEGG pathway enrichment analysis, thirteen enriched key pathways were presented in the down-regulated CNV-driven DEGs, including chemical carcinogenesis and several metabolic pathways, such as metabolism of xenobiotics by cytochrome P450, tyrosine metabolism, and retinol metabolism (Fig. 2a). In addition, eight KEGG pathways were significant for the up-regulated CNV-driven DEGs, such as cell cycle, DNA replication, and Hippo signaling pathway (Fig. 2b).

### PPI network construction and module screening from the PPI network

**Figure 3:**
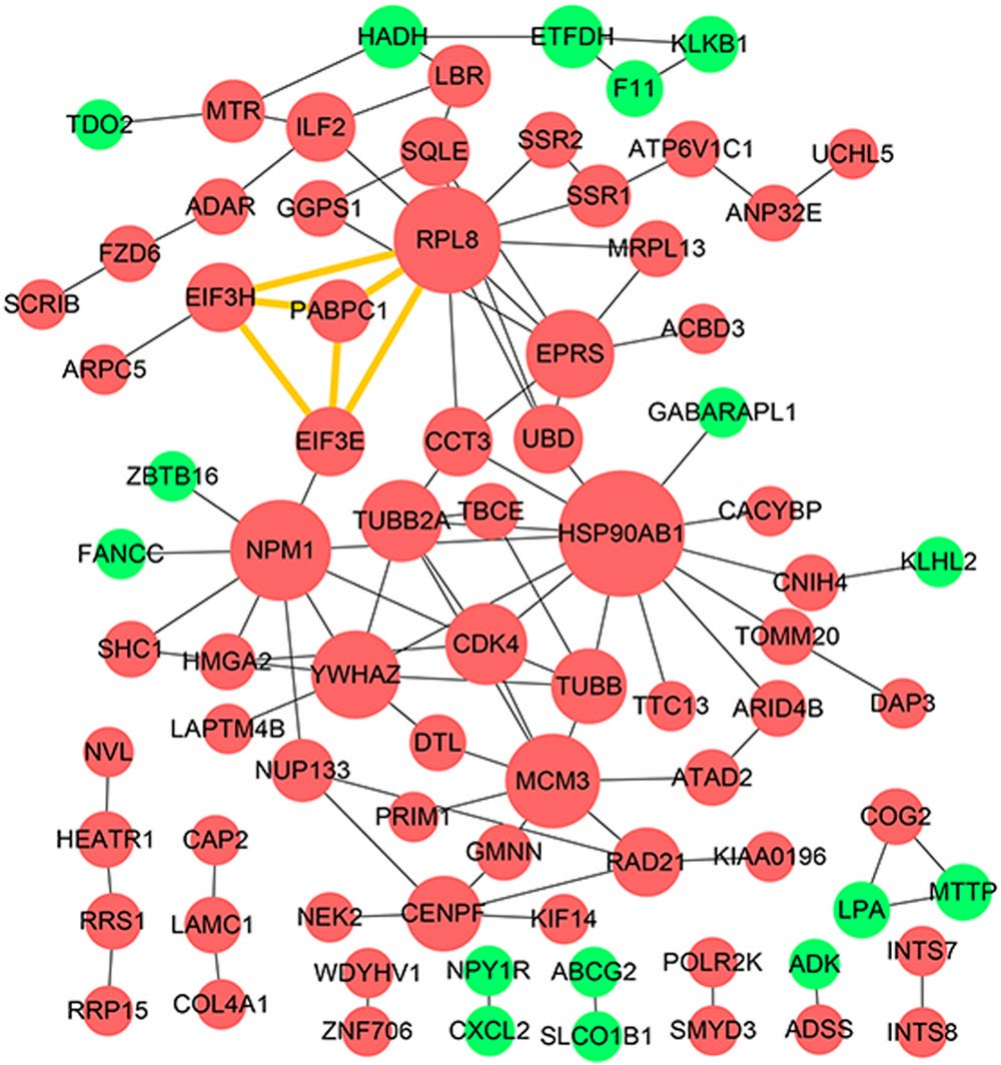
Protein-protein interaction (PPI) network constructed by STRING database for CNV-driven DEGs. Red and green circles represent up-regulated and down-regulated CNV-driven DEGs, respectively. Nodes with yellow edges are genes of the significant module. The size of the circles represents the degrees of the CNV-driven DEGs.

The STRING database was used to analyze the PPI relationships of the CNV-driven DEGs. The PPI network of the CNV-driven DEGs consisted of 81 nodes and 103 edges, including sixteen down-regulated and 65 up-regulated CNV-driven DEGs (Fig. 3). Node degrees ≥ eight was selected as the threshold. Heat shock protein 90 alpha family class B member 1 (HSP90AB1), ribosomal protein L8 (RPL8), nucleophosmin (NPM1), and mini-chromosome maintenance complex component 3 (MCM3) were selected as the hug genes.

We then screened the significant module of the PPI network using plug-ins MCODE. In our study, four nodes were involved in this module and one hub gene with higher degrees, including eukaryotic translation initiation factor 3 subunit E (EIF3E), poly(A) binding protein cytoplasmic 1 (PABPC1), eukaryotic translation initiation factor 3 subunit H (EIF3H), and RPL8. (Fig. 3) Interestingly, all of these four genes were located at chromosome 8. Functional and KEGG pathway enrichment analysis revealed that genes in this module were mainly associated with translational initiation, RNA transport, and hepatitis C pathway (Supplementary Table 5).

### Validation studies of the hub CNV-driven DEGs in large cohort of patients with HCC

We assessed the prognostic value of four hub CNV-driven DEGs through the data of 180 HCC patients from our previous study (GSE14520), and follow-up data of 30 HCC patients who were involved in integrated analysis with CNVs were not included. Patients were divided into a high expression group and a low expression group, using the mean expression levels of the hug genes as the cutoff point. According to the low and high expression of each hub gene, overall survival (OS) and time to recurrence (TTR) for patients with HCC were obtained.

**Figure 4:**
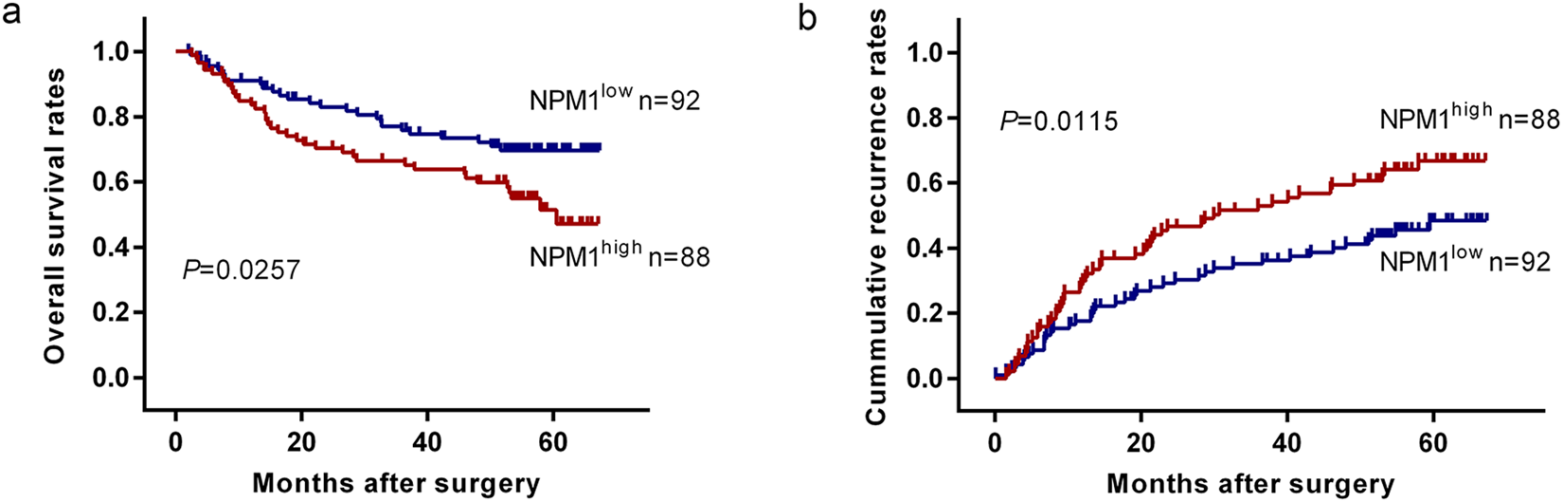
Kaplan-Meier curves for overall survival (OS) and time to recurrence (TTR) based on NPM1 expression in HCC cohort (n = 180).

Obviously, the Kaplan–Meier analysis revealed that individuals with high intra-tumor expression levels of NPM1 had a significantly worse prognosis than those with low intra-tumor expression levels of NPM1, as reflected by the OS and TTR (*P* = 0.0257 and *P* = 0.0115, respectively; Fig. 4). NPM1 was located at chromosome 5. To further evaluate the prognostic value of NPM1 for HCC patients, univariate and multivariate analyses were performed with the clinic-pathological characteristics and NPM1 (Table 3). In the univariate analysis, serum alpha-fetoprotein (AFP) level, tumor-nodes-metastases (TNM) stage, and Barcelona Clinic Liver Cancer (BCLC) stage were revealed to significantly associated with OS and TTR of HCC patients. Gender was also revealed to be significantly associated with TTR of HCC patients. NPM1 was also significantly associated with both OS and TTR. No significant prognostic associations were found among the other characteristics including age, HBV status, ALT, liver cirrhosis, tumor size and multinodular of NPM1 for OS or TTR (Table 3). In the multivariate analysis, NPM1 was still revealed to be independent prognostic indicator for both OS (hazard ratio = 2.197, 95% CI = 1.298–3.721, *P* = 0.003) and TTR (hazard ratio = 1.846, 95% CI = 1.207–2.822, *P* = 0.005) (Table 3). However, HSP90AB1, RPL8, and MCM3 had no significant differences related to OS and TTR (Supplementary Figure 1). Moreover, copy number duplications of NPM1 were presented at four of the thirty-three paired samples using whole-genome SNP 6.0 array. BCLC stages and TNM stages of the four patients were stage A and stage I.

**Table 3:** Univariate and multivariate analysis of factors associated with survival and recurrence in 180 HCCs.

Factors	OS			TTR		
	Univariate	Multivariate		Univariate	Multivariate	
	*P*	HR (95% CI)	*P*	*P*	HR (95% CI)	*P*
Gender (female vs. male)	0.098		NA	**0.031**		NS
Age (<50 y vs. ≥50 y)	0.640		NA	0.525		NA
HBV(AVR-CC vs. CC)	0.593		NA	0.484		NA
ALT (>50 U/L vs. ≤50 U/L)	0.428		NA	0.071		NA
AFP (>300 ng/mL vs. ≤300 ng/mL)	**0.041**		NS	0.391		NA
Cirrhosis (yes vs. no)	0.064		NA	0.193		NA
Tumor size (>5 cm vs. ≤5 cm)	0.065		NA	0.459		NA
Multinodular (yes vs. no)	0.056		NA	0.281		NA
TNM stage (III vs. II vs. I)						
Overall	**<0.001**		NS	**<0.001**		**0.012**
II vs. I	**0.021**		NS	**0.001**	**1.993 (1.210–3.282)**	**0.007**
III vs. I	**<0.001**		NS	**<0.001**		NS
BCLC stage (C vs. B vs. A vs. 0)						
Overall	**<0.001**		**<0.001**	**<0.001**		**0.002**
A vs. 0	0.064		NA	0.128		NA
B vs. 0	**0.013**	**6.747 (1.394–32.658)**	**0.018**	**0.010**	**3.181 (1.055–9.596)**	**0.040**
C vs. 0	**<0.001**	**27.456 (6.169–122.201)**	**<0.001**	**<0.001**	**11.855 (3.208–43.810)**	**<0.001**
NPM1 (high vs. low)	**0.028**	**2.197 (1.298–3.721)**	**0.003**	**0.013**	**1.846 (1.207–2.822)**	**0.005**

Univariate analysis was calculated by the Kaplan–Meier method (log-rank test). Multivariate analysis was done using the Cox multivariate proportional hazard regression model with stepwise manner.

OS, overall survival; TTR, time to recurrence; AVR-CC, active viral replication chronic carrier; CC, chronic carrier. TNM, tumor-nodes-metastases; HR, hazard ratio; CI, confidential interval; NA, not adopted; NS, not significant.

To make sure whether the association of NPM1 expression with clinical outcomes of patients depends on BCLC stage and TNM stage, additional subgroup analyses of BCLC stage and TNM stage were applied respectively. As presented in Supplementary Figure 2, both OS and TTR strongly associated with BCLC (0-A) group (*P* = 0.0286 and *P* = 0.0131, respectively; Supplementary Figure 2a and b), TNM (I–II) group (*P* = 0.0482 and *P* = 0.0121, respectively; Supplementary Figure 2c and d). On the other hand, NPM1 failed to predict tumor outcomes in BCLC (B-C) group (*P* > 0.05) and TNM (III) group (*P* > 0.05).

Moreover, to further explore the association between NPM1 expression and HCC patients’ clinical outcome, the TCGA hepatocellular carcinoma cohort was analyzed. HCC patients with higher NPM1 expression had a shorter OS compared to those with lower NPM1 expression (Supplementary Figure 2e; HR = 1.501, 95% CI = 1.024–2.356, *P* = 0.0389). Moreover, HCC patients with higher NPM1 expression had a higher cumulative recurrence rates compared to those with lower NPM1 expression (Supplementary Figure 2f; HR = 1.690, 95% CI = 1.147–2.757, *P* = 0.0104).

### Validation of the expression pattern of NPM1 in HCC tissues and cell lines

**Figure 5:**
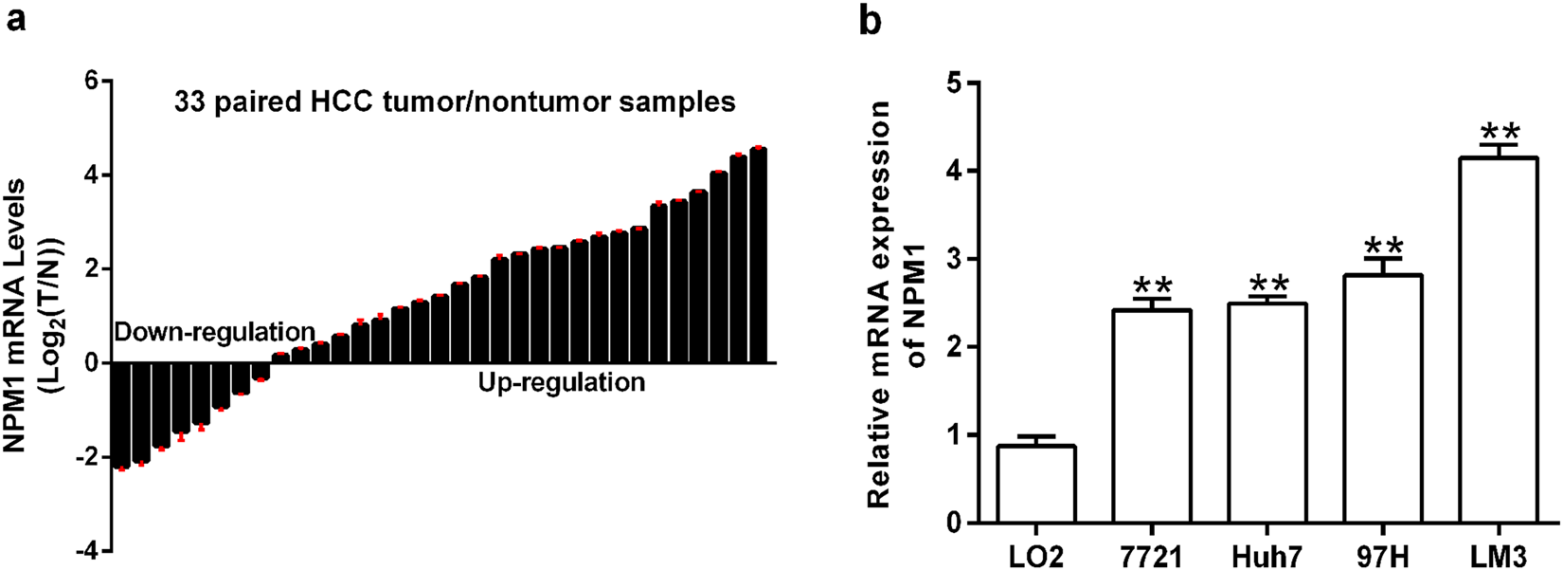
Up-regulation of NPM1 in HCCs. (**a**) NPM1 mRNA expression in 33 paired HCC tumor and adjacent non-tumor tissues. (**b**) mRNA levels of NPM1 were quantified in four HCC cells and a non-malignant liver cell (L02). GAPDH was acted as an internal control. **P < 0.01.

To investigate the expression pattern of NPM1 in HCC tissues, qRT-PCR was utilized to detect the messenger RNA (mRNA) levels of NPM1 in 33-paired HBV related HCC tumor and adjacent non-tumor tissues. Our results showed that an up-regulation of NPM1 was observed in 75.76% (25/33) of HCC patients (Fig. 5a). Moreover, we detected the mRNA levels of NPM1 in human HCC cells. Interestingly, we found that NPM1 expression was remarkably higher in HCC cells compared to nonmalignant liver cells (L02) (Fig. 5b). Our results showed that NPM1 mRNA was frequently overexpressed in human HCC.

Collectively, these data suggested that high expression of NPM1 was a valuable index for dismal prognosis in HBV related HCC patients.

## Discussion

The pathogenesis of HCC is a multi-step process, and molecular alterations at both genetic and epigenetic levels have been shown to drive hepatocarcinogenesis^14^. Therefore, understanding the etiological risk factors and molecular mechanisms of HCC progression is of a critical importance for prevention, diagnosis, and treatment. In the present study, we performed an integrated analysis of CNVs and gene expression profiling to identify the DEGs with alterations in genomic segments. We analyzed the CNVs by using high-resolution SNP 6.0 arrays and gene expression profiling in 33 paired and 30 paired tissues respectively. All of patients selected were hepatitis B virus surface antigen (HBsAg) positive. To improve the accuracy of screening out CNV-driven DEGs, the baseline characteristics of the two cohort patients were similar.

In our study, a total of 138 CNV-driven DEGs were identified with the same expressional tendencies, including 93 CNV-driven DEGs exhibiting increased expression in the duplicated regions and 45 showing decreased expression in the deleted regions. The highest number of duplications and deletions were documented at chromosome 1 and 4, respectively, which is in accordance with previous studies. It was reported that the duplication of chromosome 1q can be detected in 58–78% of HCC patients, and the deletion of chromosome 4q may be involved in hepatitis B virus-related hepatocarcinogenesis and the elevation of serum alpha-fetoprotein^7, 15^. Functional enrichment analysis of the 93 up-regulated CNV-driven DEGs showed that they were mainly involved in positive regulation of translation, cell division and DNA damage response, which were very closely related to tumor, while the 45 down-regulated CNV-driven DEGs were mainly enriched in ethanol oxidation, xenobiotic metabolic process and metabolic process. To our knowledge, defects in asymmetric cell division can lead to the formation of tumors^16^. Meanwhile, it was reported that DNA damage response was a double-edged sword in cancer prevention and cancer therapy^17^. At present, several studies have reported that ethanol oxidation would delay or impair the replication of hepatocytes^18, 19^. Furthermore, pathway enrichment annotation showed that the up-regulated CNV-driven DEGs were mainly involved in cell cycle, DNA replication, and Hippo signaling pathway; and the down-regulated CNV-driven DEGs were involved in chemical carcinogenesis and several metabolic pathways. It was consistent with our knowledge that the main course for cancer development and progression was the defective function of cell cycle and DNA replication^20–22^. The Hippo signaling pathway was reported to be a critical regulator of mammalian liver growth and a potent suppressor of liver tumor formation^23^. What’s more, chemical carcinogenesis and metabolic pathways were also very important in the development of HCC. Therefore, closely monitoring these signaling pathways may help us better diagnosis and predict the progression of HCC.

In this study, PPI network with CNV-driven DEGs were also constructed. EIF3E, PABPC1, EIF3H, and RPL8 were screened from the PPI network as the most significant module. EIF3 is the largest and most complex of the initiation factors, consisting of 13 putative protein subunits. EIF3 and its subunits regulate translation of a subset of mRNAs involved in many cellular processes, such as proliferation, apoptosis, DNA repair, and cell cycle^24^. EIF3E (called *Int-6*) was detected to be at low levels in non-small cell lung carcinomas, which suggested that EIF3E may function as a tumor suppressor^25^. Moreover, previous studies suggested that overexpression of EIF3H was associated with advanced cancer stages and poor prognosis in prostate and liver cancer^26, 27^. Zhang *et al*. reported that PABPC1 was aberrantly expressed in HCC, especially in high grade HCC. And they also found that PABPC1 played a role as an oncogene in HCC, which accelerated the cell proliferation and promoted entry into the S Phase and progression to the G2/M phase^28^. RPL8, a member of the L2P family of ribosomal proteins, is a component of the 60 S ribosomal subunit in eucaryotic cells. The duplication of RPL8 was reported to be associated with the pathogenesis of osteosarcoma^29^, but the biological functions of RPL8 in HCC remains unclear. Moreover, all of these four genes were located at chromosome 8. It was reported that the duplication of chromosome 8 was a frequently detected genomic alteration in HCC (48–66%), and may confer a selective growth advantage on HCC cells^7^. Furthermore, functional and KEGG pathway enrichment analysis revealed that genes in this module were mainly associated with translational initiation, which was closely involved in the progression of HCC.

Additionally, a total of four DEGs were selected as the top degree hub genes from the PPI network, including HSP90AB1, RPL8, NPM1, and MCM3. Through analyzing the clinical relevance of these hub genes in HCC, only NPM1 was found to be significantly correlated with OS and TTR. NPM1, copy number duplication on chromosome 5 in our study, was overexpressed in primary HCC tissues compared to those in non-tumor tissues. To date, NPM1 is an abundant phosphoprotein which moves between the nucleus and the cytoplasm^30^. Previous studies showed that NPM1 is a multifunctional protein involved in many cellular processes such as DNA repair and ribosome biogenesis^31, 32^. To our knowledge, genetic alterations of NPM1 have been found in various hematological malignancies, such as acute myeloid leukemia^33^. Meanwhile, the expression level of NPM1 was reported to be overexpressed in many solid tumors and correlated with the progression and recurrence of tumor^34, 35^. In present study, the clinically relevant data presented showed that patients with HCC and high tumor NPM1 expression levels had decreased OS times and earlier TTR compared to those with low tumor NPM1 expression levels. Those findings are consistent with previous studies. In the meanwhile, NPM1 expression could stratify HCC patients by survival analyses in BCLC stage (0-A) and TNM stage (I-II) subgroups, which might redefine risk stratification of HCC patients. In addition, we found that copy number duplications of NPM1 were presented in four patients whose BCLC stages were A and TNM stages were I. It represented that the genetic changes of NPM1 occurred in the early stages of HCC. It was believed that the genetic changes of early liver cancer were relatively more favorable for early clinical diagnosis and predicting prognosis.

Many target proteins, including ARF, p53, p65, and NF-κB, were thought to be involved in the regulation of NPM1^36, 37^. A study showed that NF-κB required the chaperone-like function of NPM1 for DNA binding. Moreover, NPM1 was required for efficient inflammatory gene expression induced by tumor necrosis factor alpha (TNF-α) and lipopolysaccharide in fibroblasts and macrophages^37^. In addition, it can be seen from the PPI network that FANCC (Fanconi anemia complementation group C), a DNA repair gene^38^, was closely associated with NPM1. FANCC was a down-regulated CNV-driven gene in HCC samples, while NPM1 was the up-regulated CNV-driven gene. This may suggest that DNA repair defects/deficiencies may be important in the generation of CNVs.

In conclusion, this study provided an integrated analysis of CNVs and gene expression profiling to identify CNV-driven DEGs in HBV related HCC. EIF3E, PABPC1, EIF3H, and RPL8 which were all located at chromosome 8 from the module analysis, to the best of our knowledge, were demonstrated to be related to the progression of HCC. Furthermore, NPM1, the hub gene located at chromosome 5, was identified to be associated with the overall and recurrence-free survival time in postoperative patients with HCC, indicating that NPM1 may be a novel marker for predicting prognosis and a therapeutic target for HBV related HCC. However, more in-depth studies are needed to further verify the mechanisms between CNVs and gene expression profiling.

## Methods

### Patient selection

Thirty-three primary HCC tumors and paired adjacent non-tumor tissues stored at −80 °C were obtained from patients who underwent hepatectomy during the period from January to July 2009 at Zhongshan Hospital, Fudan University. The inclusion and exclusion criteria of the patient cohort include (1) no patients have received systemic or local treatment before operation, (2) no cases with extrahepatic metastases, (3) all patients having a distinctive postoperative pathologic diagnosis of HCC, (4) all patients undergoing primary and curative liver resection, (5) HBsAg (+); (6) and with complete clinic-pathologic and follow-up data. This study was approved by the Ethics Committee of Zhongshan Hospital, Fudan University and was conducted in accordance with the relevant guidelines. Written informed consent was obtained from all participants.

The gene expression profiles and clinical follow-up data of GSE14520, as reported in our previous studies, were acquired from the National Center for Biotechnology Information (NCBI) Gene Expression Omnibus (GEO) database. The GSE14520 dataset contained 210 paired HCC and non-tumor samples. In the process of integrating the analysis of CNVs and gene expression profiling, thirty patients, whose baseline characteristics were similar to the thirty-three patients, were selected. And another 180 patients of GSE14520 were selected to validate the importance of CNV-driven genes.

### SNP array analysis and data preparation

DNA was isolated using the QIAamp DNA Mini Kit according to the manufacturer’s protocol. DNA was extracted, amplified and hybridized onto an Affymetrix Genome-Wide human SNP array 6.0 according to the manufacturer’s instructions (Affymetrix Inc.). First, R packages of hapmapsnp6, oligoClasses, crlmm and ff were used to read the primary data (in CEL format) of Affy SNP 6.0 array, in addition to spotting the sites of SNPs. Second, R package of pd.genomewidesnp.6 was used to obtain the annotations of sites of each SNP spotted. Third, affy2sv package along with penncnv software helped to obtain all information regarding CNVs. According to the genetic annotations obtained, we completed the awk procedure and got the genetic information within the CNV domains. Partek Genomics Suite, version 6.4 (Partek Inc., St. Louis, MO, USA) was also used for copy number analysis. Hidden Markov Model (HMM) algorithm was used to detect the regions of CNVs in the standard Partek workflow for paired samples. The overall hybridization quality was estimated by the call rate index obtained from Genotyping Console Software (GTC 3.0, birdseed algorithm using default parameter settings). The criteria to define significant CNVs were as follows: the duplication was set at above 3, while deletion was set at below 1; the gene of copy number duplications or deletions had occurred in at least 10% (4 samples) of the total samples; the overlapping common regions among multiple tumors were calculated.

### Statistical analysis of gene expression profiling

Microarray data from GSE14520 datasets were directly downloaded from GEO. The detailed process of performing gene expression profiling has been published in our previous study^39^. Briefly, raw CEL files were processed for background correction and quantile normalization (median scaling) using the robust multi-array averaging (RMA) method. The Affy package from Bioconductor and Affy probe annotation files (Affymetrix HT Human Genome U133A chips) were applied to preprocess the gene expression profile data. In the end, gene expression matrix was obtained. Thirty paired samples were selected to identify differentially expressed genes (DEGs).

The classical t test was applied in our analysis to identify DEGs between paired HCC and non-tumor samples. Statistically significant expressed genes were identified using the mixed model analysis of variance with a false discovery rate (Benjamini–Hochberg test) adjusted *P* value of < 0.05 and fold-change values of −2 to 2.

### Integration of copy number variation and gene expression profiling

To identify the significant driver genes that exhibited CNV and gene expression alterations, we integrated the data of significant CNVs and DEGs. Hierarchical clustering analysis of the CNV-driven DEGs was performed using the pheatmap package in R^40^.

### Functional and pathway enrichment analysis

The Database for Annotation, Visualization and Integrated Discovery (DAVID, https://david.ncifcrf.gov/) is the most common tool to analyze the functional enrichment of genes^41^. Gene ontology (GO) enrichment analysis was performed for identified CNV-driven DEGs using DAVID online tool^42^. Kyoto Encyclopedia of Genes and Genomes (KEGG, http://www.genome.jp/kegg/) database shows the way that genes or other molecules act^43^. KEGG Orthology Based Annotation System (KOBAS, http://kobas.cbi.pku.edu.cn/index.php) is a web server for annotating and identifying enriched pathways and associated diseases for large gene lists^44^. KEGG pathway enrichment analysis for identified CNV-driven DEGs was conducted using KOBAS 2.0. The threshold for these analyses was set as *P* value < 0.05.

### Protein-protein interaction (PPI) network construction and module screening

Search Tool for the Retrieval of Interacting Genes (STRING, http://www.string-db.org/) is an online tool and biological database that includes comprehensively predicted and known interaction information^45^. To evaluate the functional interactions among CNV-driven DEGs, we mapped the DEGs to STRING. The combined score >0.4 was used as the cut-off criterion to screen out the validated interactions. Then, PPI networks were constructed using the Cytoscape software (version 3.4.0, available at http://www.cytoscape.org/)^46^. Then, the plug-in Molecular Complex Detection (MCODE) was performed to screen modules of PPI network with the criteria as follows: MECODE scores >3 and number of nodes >3^47^. The functional and pathway enrichment analysis of genes in the module were performed by DAVID and KOBAS, respectively. *P* < 0.05 was considered to be statistically significant.

### The Cancer Genome Atlas (TCGA) data acquisition

A dataset including information about mRNA expression and clinical characteristics of HCC patients (n = 424 for TCGA hepatocellular carcinoma, gene expression by RNAseq with IlluminaHiSeq) was acquired from the TCGA website (https://tcga-data.nci.nih.gov/tcga/). The expression levels of NPM1were collected for each case and divided into the high expression and the low expression groups using the mean expression levels of the gene as the cutoff point.

### Cell lines

The human HCC cell lines, SMMC-7721, Huh7, and a normal hepatocyte cell line (L02) were purchased from the cell bank of the Chinese Academy of Sciences (Shanghai, China). MHCC97H and MHCCLM3 were established at the Liver Cancer Institute, Zhongshan Hospital, Fudan University, Shanghai, China. Cell lines were cultured in Dulbecco’s modified Eagle’s medium (DMEM) (HyClone, Logan, UT, USA) and supplemented with 10% fetal bovine serum (Invitrogen, Carlsbad, CA, USA), penicillin (100 unites/ml) and streptomycin (100 ug/ml) at 37 °C humidified incubator under an atmosphere of 5% CO_2_ in air.

### RNA isolation and quantitative reverse transcription PCR (qRT-PCR)

The total RNA was isolated from HCC tissues and cells with TRIzol reagent (Invitrogen, USA) and reverse-transcribed to cDNA using PrimeScript RT reagent kit (Takara, Japan). The qRT-PCR was performed for assessment of mRNA expression according to the manufacturer’s protocols by an ABI Prism 7500 Sequence Detection system (Applied Biosystems, Foster City, CA, USA). Primers were designed as follows: NPM1, forward: 5′-CTCGCGAGATCTTCAGGGTC-3′ and reverse: 5′-CGGCCTTTAGTTCACAACCG-3′; GAPDH, forward: 5′-CTGGGCTACACTGAGCACC-3′ and reverse: 5′-AAGTGGTCGTTGAGGGCAATG-3′. Then, the 2^−ΔΔCT^ method was used to determine the relative gene expression levels. Triplicate qRT-PCR samples were performed in each assay.

### Statistical analysis

Statistical analysis was performed with SPSS software (23.0; IBM, Armonk, NY, USA). Values are expressed as mean ± standard deviation (SD). The Student t test was used for comparisons between groups. Categorical data were analyzed by the chi-square or Fisher’s exact tests. Overall survival (OS) and Time to recurrence (TTR) were analyzed using Kaplan-Meier’s method and the log-rank test. Variables associated with OS and TTR were identified using univariate Cox proportional hazards regression models. Significant factors in univariate analysis were further subjected to a multivariate Cox regression analysis in a stepwise manner. A two-tailed *P* < 0.05 was considered statistically significant.

## Electronic supplementary material

Supplementary Information
