# Supplementary material for: Integrated Analysis of Copy Number Variations and Gene Expression Profiling in Hepatocellular carcinoma
Source: Sci Rep. 2017 Sep 5;7:10570. doi: 10.1038/s41598-017-11029-y (PMC5585301; doi:10.1038/s41598-017-11029-y)
Supplement: Supplementary file 1 — Supplementary Information [file 41598_2017_11029_MOESM1_ESM.pdf]

Supplementary Information

Integrated Analysis of Copy Number Variations and Gene Expression Profiling in Hepatocellular Carcinoma

Chenhao Zhou[1,2*], Wentao Zhang[1,2*], Wanyong Chen[1,2,3*], Yirui Yin[1,2], Manar Atyah[1,2], Shuang Liu[1,2], Lei Guo[1,2], Yi Shi[4], Qinghai Ye[1,2], Qiongzhu Dong[3,5], Ning Ren[1,2,3]

[1]Department of Liver Surgery, Liver Cancer Institute, Zhongshan Hospital, Fudan University, Shanghai, China

[2]Key Laboratory of Carcinogenesis and Cancer Invasion, Ministry of Education, China

[3]Institute of Fudan-Minhang Academic Health System, Minhang Hospital, Zhongshan Hospital, Fudan University, Shanghai, China

[4]Biomedical Research Centre, Zhongshan Hospital, Fudan University, Shanghai, China.

[5]Institutes of Biomedical Sciences, Fudan University, Shanghai, China

*These authors contributed equally to this work.

Supplementary figure 1： Kaplan-Meier curves for overall survival (OS) and time to recurrence (TTR) based on HSP90AB1(a, b), RPL8 (c, d), and MCM3 (e, f) expression in HCC cohort (n=180).

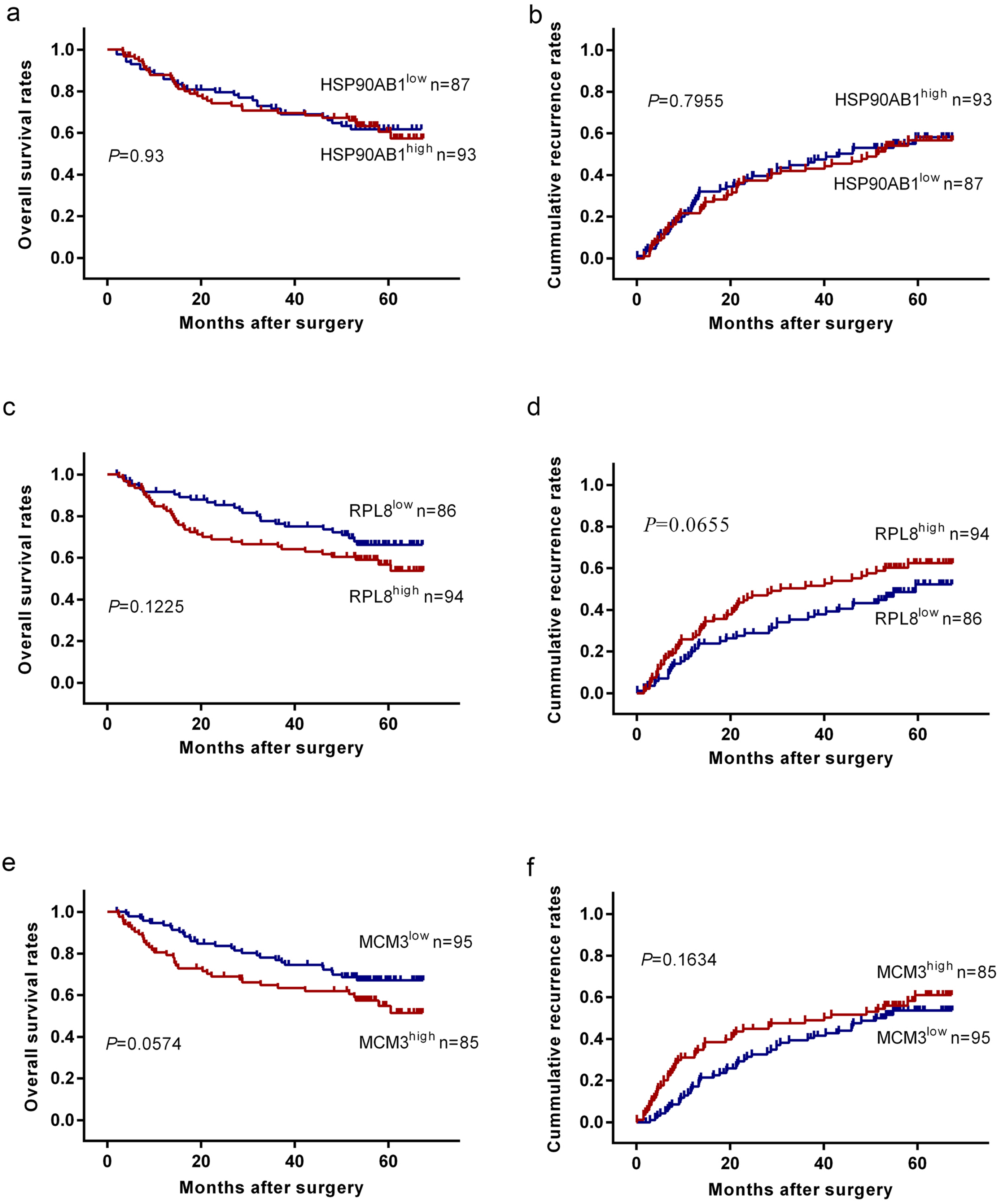

Supplementary figure 2. Kaplan-Meier curves for overall survival (OS) and time to recurrence (TTR) based on NPM1 expression according to the subgroups of BCLC stages (a, b) and TNM stages (c, d) in HCC cohort. Kaplan-Meier curves for overall survival (OS) and time to recurrence (TTR) based on NPM1 expression in TCGA hepatocellular carcinoma cohort (e, f).

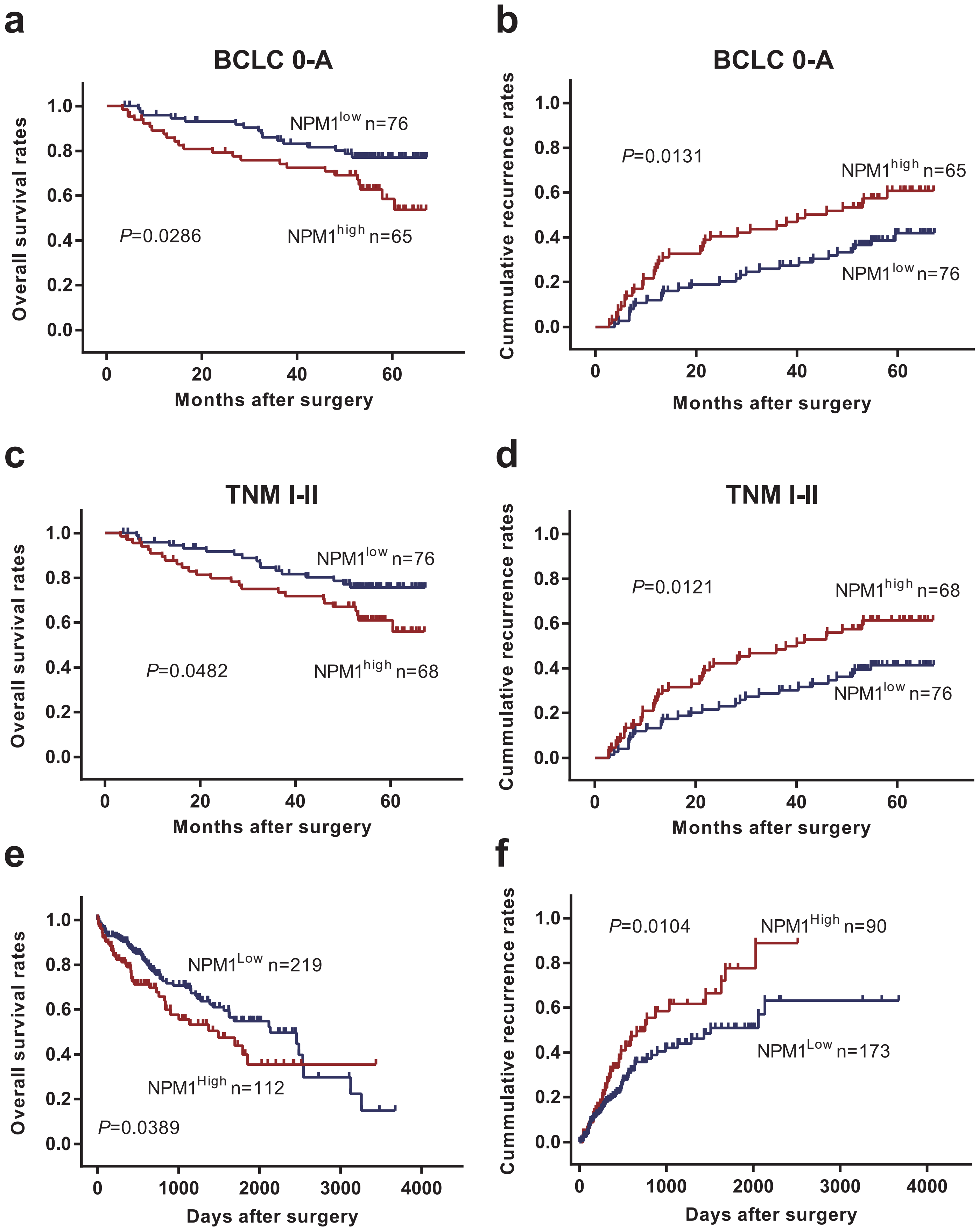

Supplementary Table 1: Distribution of copy number deletions and duplications in 33 paired samples

Samples	Numbers of Del	Mean length of Del (bp)	Numbers of Dup	Mean length of Dup (bp)
ZS-DNA9	44	76657.1	28	52812.5
ZS-DNA28	106	136536	17	76525.2
ZS-DNA27	16	124268	9	44642.1
ZS-DNA26	13	65988.4	25	93937
CN_328	694	152125	196	851896
CN_324	226	186880	300	196423
CN_320	16	111607	379	207391
CN_D306	3	66316	67	148635
CN_D304	35	128866	37	91862.3
ZS-DNA25	23	82877.8	20	25463.2
ZS-DNA24	30	76615.7	25	56478.6
ZS-DNA23	76	117200	4	88565.5
CN_D254	553	336752	281	850931
CN_D302	230	225342	198	437434
CN_D300	218	194509	381	208776
CN_D298	99	133211	686	236122
CN_D296	630	306442	834	354510
CN_D294	59	138647	343	241505
CN_D292	918	394260	216	948183
CN_D252	204	2231410	145	1034580
CN_D251	46	124932	504	217341
CN_330	21	83270.7	168	593386
ZS-DNA12	28	83967.5	12	62224.1
ZS-DNA11	51	130491	16	64030.8
CN_D318	277	2025130	154	1429950
CN_D316	9	56104.9	147	129791
CN_D228	7	127016	30	78169.2
CN_D227	44	84128.7	94	116590
CN_D253	106	110196	1749	191378
CN_D310	101	394630	312	373874
CN_D288	222	766499	244	1142320
CN_D225	188	1825560	62	2787000
CN_D224	164	423287	699	393976
Total	5457	11521722.8	8382	13826702.5
Mean	165.4	349143.1 (349.1kb)	254	418991.0 (419.0kb)

Del, deletions; Dup, duplications

Supplementary Table 2: Significant genes of copy number deletions in at least 10% (4 samples) samples.

Frequency number	Gene symbol	Samples										
11	PTPRD	ZS.DNA9	CN_D298	CN_D292	CN_D253.2	CN_D252	CN_D225	CN_328	CN_324	CN_D292	CN_328	CN_324
9	SGCZ	CN_D318	CN_D292	CN_D288	CN_D252	CN_330	CN_328	CN_324	CN_D292	CN_328		
8	PCDH9	ZS.DNA9	CN_D318	CN_D292	CN_328	CN_324	CN_D298	CN_D292	CN_328			
7	ODZ3	CN_D318	CN_D310	CN_D292	CN_D288	CN_D252	CN_D225	CN_D292				
7	EFHA2	CN_D318	CN_D292	CN_D288	CN_D252	CN_330	CN_328	CN_D292				
7	CSMD1	CN_D318	CN_D292	CN_D288	CN_D251	CN_330	CN_328	CN_D292				
7	CDKN2B-AS1	CN_D292	CN_D252	CN_D251	CN_D225	CN_324	CN_D292	CN_D225				
6	UNC5D	CN_D292	CN_D288	CN_D252	CN_330	CN_328	CN_D292					
6	TNKS	CN_D318	CN_D292	CN_D288	CN_D253.2	CN_D292	CN_D253.2					
6	SNX25	CN_D318	CN_D310	CN_D288	CN_D252	CN_D225	CN_D310					
6	SNORA59B	CN_D318	CN_D296	CN_D292	CN_D252	CN_D225	CN_D224					
6	SNORA59A	CN_D318	CN_D296	CN_D292	CN_D252	CN_D225	CN_D224					
6	SLCO1B7	CN_D318	CN_D288	CN_D224	CN_324	CN_D224	CN_324					
6	SLCO1B1	CN_D318	CN_D288	CN_D224	CN_324	CN_D224	CN_324					
6	PRH1-PRR4	CN_D318	CN_D288	CN_D254	CN_D224	CN_D288	CN_D254					
6	PRDM16	CN_D318	CN_D296	CN_D292	CN_D252	CN_D296	CN_D292					
6	PIK3C2G	CN_D318	CN_D288	CN_D224	CN_328	CN_324	CN_D224					
6	NRG1	CN_D318	CN_D292	CN_D288	CN_D252	CN_328	CN_D292					
6	MIR548O2	CN_D318	CN_D292	CN_D288	CN_D252	CN_D292	CN_D288					
6	MCPH1	CN_D318	CN_D292	CN_D288	CN_D253.2	CN_D292	CN_D253.2					
6	LRP1B	ZS.DNA9	ZS.DNA12	CN_D318	CN_D254	CN_328	CN_D254					
6	LOC728175	CN_D318	CN_D310	CN_D292	CN_D288	CN_D252	CN_D225					
6	LOC401164	CN_D318	CN_D310	CN_D288	CN_D252	CN_D225	CN_D310					
6	LOC339975	CN_D310	CN_D292	CN_D288	CN_D252	CN_D225	CN_D310					
6	LOC285501	CN_D318	CN_D310	CN_D300	CN_D292	CN_D252	CN_D225					
6	LINC00290	CN_D318	CN_D310	CN_D292	CN_D252	CN_D225	CN_D292					
6	IRF2	CN_D318	CN_D310	CN_D292	CN_D288	CN_D252	CN_D225					
6	FAT1	CN_D318	CN_D310	CN_D292	CN_D288	CN_D252	CN_D225					
6	ERBB4	ZS.DNA9	ZS.DNA12	CN_D318	CN_328	CN_324	CN_328					
6	ENPP6	CN_D318	CN_D310	CN_D292	CN_D288	CN_D252	CN_D292					
6	DPYD	CN_D318	CN_D288	CN_D252	CN_328	CN_324	CN_328					
6	DOCK5	CN_D318	CN_D292	CN_D288	CN_D252	CN_D292	CN_D288					
6	CXCL13	CN_D318	CN_D300	CN_D292	CN_D253.2	CN_D252	CN_D225					
6	CDH13	CN_D318	CN_D302.2	CN_D296	CN_D253.2	CN_D252	CN_D252					
6	ANXA3	CN_D318	CN_D300	CN_D292	CN_D252	CN_D225	CN_D292					
6	ANK2	CN_D318	CN_D292	CN_D252	CN_D225	CN_D300	CN_D253.2					
5	ZSWIM5	CN_D318	CN_D292	CN_D288	CN_D252	CN_D292						
5	ZNF827	CN_D318	CN_D292	CN_D252	CN_D225	CN_D292						
5	ZFP42	CN_D318	CN_D292	CN_D252	CN_D225	CN_D288						
5	ZDHHC2	CN_D318	CN_D292	CN_D288	CN_D252	CN_330						
5	WNK1	CN_D288	CN_D254	CN_D253.2	CN_D224	CN_D288						

5	WDR17	CN_D318	CN_D310	CN_D292	CN_D252	CN_D225
5	WDFY3	CN_D318	CN_D292	CN_D252	CN_D225	CN_D292
5	VPS13D	CN_D318	CN_D296	CN_D292	CN_D288	CN_D252
5	UNC5C	CN_D318	CN_D300	CN_D292	CN_D252	CN_D225
5	TRPM3	CN_D302.2	CN_D292	CN_D253.2	CN_D225	CN_D292
5	TRIML2	CN_D318	CN_D292	CN_D252	CN_D225	CN_D288
5	TRIM2	CN_D318	CN_D300	CN_D292	CN_D252	CN_D225
5	TRAPPC11	CN_D318	CN_D310	CN_D288	CN_D252	CN_D225
5	TMEM57	CN_D318	CN_D292	CN_D252	CN_D296	CN_D292
5	TLR3	CN_D318	CN_D310	CN_D288	CN_D252	CN_D225
5	TET2	CN_D300	CN_D292	CN_D252	CN_D225	CN_D225
5	STOX2	CN_D318	CN_D310	CN_D288	CN_D253.2	CN_D252
5	SPCS3	CN_D318	CN_D310	CN_D292	CN_D252	CN_D225
5	SPATA4	CN_D318	CN_D310	CN_D292	CN_D252	CN_D225
5	SOX5	CN_D318	CN_D253.2	CN_D224	CN_324	CN_D224
5	SORBS2	CN_D318	CN_D310	CN_D288	CN_D252	CN_D225
5	SMARCA2	CN_D292	CN_D251	CN_D225	CN_D292	CN_D225
5	SLC25A4	CN_D318	CN_D310	CN_D288	CN_D252	CN_D225
5	SLC24A2	CN_D292	CN_D252	CN_D225	CN_328	CN_D292
5	SH3RF1	CN_D318	CN_D310	CN_D292	CN_D252	CN_D225
5	SH3GL2	CN_D292	CN_D252	CN_D225	CN_328	CN_D292
5	SCOC	CN_D318	CN_D292	CN_D252	CN_D225	CN_D292
5	SCARA5	CN_D318	CN_D292	CN_D288	CN_D252	CN_D292
5	RWDD4	CN_D318	CN_D310	CN_D288	CN_D252	CN_D225
5	RNF150	CN_D318	CN_D292	CN_D252	CN_D225	CN_D292
5	RGS3	CN_D302.2	CN_D292	CN_D225	CN_D302.2	CN_D292
5	RGS1	ZS.DNA27	ZS.DNA26	ZS.DNA25	ZS.DNA24	ZS.DNA11
5	RBFOX1	CN_D318	CN_D300	CN_D298	CN_D252	CN_D300
5	RASGEF1B	CN_D318	CN_D292	CN_D253.2	CN_D252	CN_D225
5	RAPGEF1	CN_D302.2	CN_D292	CN_D225	CN_D302.2	CN_D292
5	RAP1GDS1	CN_D318	CN_D298	CN_D292	CN_D252	CN_D225
5	RALGPS1	CN_D302.2	CN_D292	CN_D225	CN_D292	CN_D225
5	PSD3	CN_D318	CN_D292	CN_D288	CN_D252	CN_D288
5	PRUNE2	CN_D302.2	CN_D292	CN_D225	CN_D302.2	CN_D253.2
5	PIK3R5	CN_D318	CN_D294	CN_D292	CN_D225	CN_D224
5	PEBP4	CN_D318	CN_D292	CN_D288	CN_D252	CN_D288
5	PCM1	CN_D318	CN_D292	CN_D288	CN_D253.2	CN_D252
5	PAX7	CN_D318	CN_D296	CN_D292	CN_D252	CN_D292
5	PALM2-AKAP2	CN_D302.2	CN_D292	CN_D225	CN_D302.2	CN_D292
5	NUP188	CN_D302.2	CN_D292	CN_D253.2	CN_D225	CN_D292
5	NTRK2	CN_D302.2	CN_D292	CN_D225	CN_D302.2	CN_D292
5	NEK1	CN_D318	CN_D310	CN_D292	CN_D252	CN_D225
5	NEGR1	CN_D318	CN_D288	CN_D252	CN_330	CN_328

5	NCAM2	CN_D300	CN_D254	CN_328	CN_D300	CN_328
5	MYOCD	CN_D318	CN_D296	CN_D292	CN_D224	CN_D224
5	MUTYH	CN_D318	CN_D310	CN_D292	CN_D288	CN_D252
5	MTNR1A	CN_D318	CN_D310	CN_D288	CN_D252	CN_D225
5	MPRIP	CN_D318	CN_D225	CN_D224	CN_D292	CN_D224
5	MIR1305	CN_D318	CN_D310	CN_D288	CN_D252	CN_D225
5	MIR1243	CN_D318	CN_D252	CN_D225	CN_D300	CN_D253.2
5	MGST1	CN_D318	CN_D288	CN_D254	CN_D253.2	CN_D224
5	MGC45800	CN_D318	CN_D310	CN_D288	CN_D252	CN_D225
5	MFAP3L	CN_D318	CN_D310	CN_D292	CN_D252	CN_D225
5	MARCH1	CN_D318	CN_D292	CN_D252	CN_D225	CN_D292
5	MAF	CN_D318	CN_D296	CN_D252	CN_D302.2	CN_D253.2
5	LRBA	CN_D318	CN_D292	CN_D252	CN_D225	CN_D253.2
5	LPHN3	CN_D292	CN_D252	CN_D225	CN_328	CN_D225
5	LOC389247	CN_D318	CN_D310	CN_D288	CN_D225	CN_D252
5	LOC285441	CN_D318	CN_D310	CN_D288	CN_D252	CN_D225
5	LOC100506085	CN_D318	CN_D310	CN_D292	CN_D252	CN_D225
5	LOC100129858	CN_D318	CN_D292	CN_D252	CN_D225	CN_D292
5	LIN54	CN_D318	CN_D292	CN_D252	CN_D225	CN_D292
5	KLRC4-KLRK1	CN_D318	CN_D288	CN_D254	CN_D224	CN_D288
5	KLKB1	CN_D318	CN_D310	CN_D288	CN_D252	CN_D225
5	KIF1B	CN_D318	CN_D292	CN_D252	CN_D296	CN_D292
5	KIAA1430	CN_D318	CN_D310	CN_D288	CN_D252	CN_D225
5	KIAA0922	CN_D318	CN_D292	CN_D252	CN_D225	CN_D292
5	KDM4C	CN_D316	CN_D298	CN_D292	CN_D227	CN_D225
5	KCTD9	CN_D318	CN_D292	CN_D288	CN_D252	CN_D292
5	INPP4B	CN_D318	CN_D292	CN_D252	CN_D225	CN_328
5	ING2	CN_D318	CN_D310	CN_D288	CN_D225	CN_D252
5	IL8	CN_D318	CN_D300	CN_D292	CN_D252	CN_D225
5	HPDL	CN_D318	CN_D310	CN_D292	CN_D288	CN_D252
5	GRIN2B	CN_D318	CN_D288	CN_D254	CN_D224	CN_D224
5	GRIK1	CN_D300	CN_D254	CN_328	CN_D300	CN_D254
5	GPM6A	CN_D318	CN_D310	CN_D292	CN_D252	CN_D225
5	GC	CN_D292	CN_D252	CN_D225	CN_328	CN_328
5	GAS7	CN_D318	CN_D292	CN_D225	CN_D224	CN_D224
5	GALNTL6	CN_D310	CN_D292	CN_D252	CN_D225	CN_D225
5	FRAS1	CN_D318	CN_D300	CN_D292	CN_D252	CN_D225
5	FOPNL	ZS.DNA28	CN_D318	CN_D298	CN_D294	CN_D252
5	FLJ38576	CN_D318	CN_D310	CN_D288	CN_D252	CN_D225
5	FHDC1	CN_D318	CN_D298	CN_D292	CN_D252	CN_D225
5	FAM92A3	CN_D318	CN_D310	CN_D288	CN_D252	CN_D225
5	FAM47E	CN_D318	CN_D292	CN_D252	CN_D225	CN_D292
5	FAM190A	CN_D318	CN_D292	CN_D252	CN_D225	CN_D292

5	FAM149A	CN_D318	CN_D310	CN_D288	CN_D252	CN_D225
5	F11	CN_D318	CN_D310	CN_D288	CN_D252	CN_D225
5	ETV6	CN_D318	CN_D288	CN_D254	CN_D224	CN_D224
5	ERC1	CN_D288	CN_D254	CN_D253.2	CN_D224	CN_D254
5	EPHB2	CN_D318	CN_D292	CN_D288	CN_D252	CN_D292
5	EPB41L4B	CN_D302.2	CN_D292	CN_D253.2	CN_D225	CN_D292
5	ELP3	CN_D318	CN_D288	CN_D252	CN_D292	CN_D252
5	ELOVL6	CN_D318	CN_D292	CN_D252	CN_D225	CN_D292
5	EIF4E	CN_D318	CN_D298	CN_D292	CN_D252	CN_D225
5	DPYSL2	CN_D318	CN_D292	CN_D288	CN_D252	CN_D288
5	DLGAP2	CN_D318	CN_D292	CN_D288	CN_D292	CN_D288
5	DLC1	CN_D318	CN_D292	CN_D288	CN_D292	CN_D288
5	DCTD	CN_D318	CN_D288	CN_D252	CN_D225	CN_D292
5	DCHS2	ZS.DNA23_	CN_D318	CN_D292	CN_D252	CN_D225
5	CYP4V2	CN_D318	CN_D310	CN_D288	CN_D252	CN_D225
5	CSGALNACT1	CN_D318	CN_D292	CN_D288	CN_D252	CN_328
5	CNOT6L	CN_D318	CN_D300	CN_D292	CN_D252	CN_D225
5	CLCN3	CN_D318	CN_D310	CN_D292	CN_D252	CN_D225
5	CDK8	CN_D318	CN_D292	CN_D224	CN_D292	CN_D224
5	CBR4	CN_D318	CN_D310	CN_D292	CN_D252	CN_D225
5	C4orf38	CN_D318	CN_D310	CN_D288	CN_D252	CN_D225
5	C4orf27	CN_D318	CN_D310	CN_D292	CN_D252	CN_D225
5	C4orf21	CN_D318	CN_D292	CN_D252	CN_D225	CN_D292
5	BNC2	CN_D298	CN_D292	CN_D252	CN_D225	CN_324
5	BMPR1B	CN_D318	CN_D292	CN_D252	CN_D225	CN_328
5	BICD1	CN_D318	CN_D253.2	CN_D224	CN_D253.2	CN_D224
5	ATP8A2	CN_D318	CN_D292	CN_D224	CN_D292	CN_D224
5	ASTN2	CN_D292	CN_D225	CN_328	CN_D292	CN_328
5	ASB5	CN_D318	CN_D310	CN_D292	CN_D252	CN_D225
5	ART4	CN_D318	CN_D288	CN_D254	CN_D253.2	CN_D224
5	ARHGEF11	ZS.DNA25	ZS.DNA24	ZS.DNA11	CN_328	CN_324
5	ARHGAP24	CN_D318	CN_D292	CN_D252	CN_D225	CN_D292
5	ARHGAP10	CN_D318	CN_D292	CN_D253.2	CN_D252	CN_D225
5	ARFIP1	CN_D318	CN_D298	CN_D292	CN_D252	CN_D225
5	ANXA10	CN_D318	CN_D292	CN_D252	CN_D225	CN_328
5	ANGPT2	CN_D318	CN_D292	CN_D288	CN_D253.2	CN_D253.2
5	AKR7L	CN_D318	CN_D296	CN_D292	CN_D252	CN_D292
5	AGA	CN_D318	CN_D310	CN_D292	CN_D252	CN_D225
5	ABCG2	CN_D318	CN_D300	CN_D252	CN_D225	CN_D292
5	AADAT	CN_D318	CN_D310	CN_D292	CN_D252	CN_D225
4	ZSWIM7	CN_D318	CN_D225	CN_D224	CN_D224	
4	ZNF48	CN_D318	CN_D304	CN_D294	CN_D252	
4	ZNF330	CN_D318	CN_D292	CN_D252	CN_D225	

4	ZNF200	CN_D318	CN_D300	CN_D298	CN_D252
4	ZMYM6NB	CN_D318	CN_D292	CN_D288	CN_D252
4	ZMYM6	CN_D318	CN_D292	CN_D288	CN_D252
4	ZMYM5	CN_D298	CN_D224	CN_D292	CN_D224
4	ZMYM4	CN_D318	CN_D292	CN_D288	CN_D252
4	ZMYM2	CN_D318	CN_D292	CN_D224	CN_D224
4	ZMYM1	CN_D318	CN_D292	CN_D288	CN_D252
4	ZBTB40	CN_D318	CN_D292	CN_D252	CN_D292
4	ZBTB16	CN_D318	CN_D300	CN_D254	CN_D300
4	YTHDF2	CN_D318	CN_D296	CN_D292	CN_D252
4	YBX2	CN_D318	CN_D225	CN_D224	CN_D292
4	XPO7	CN_D318	CN_D292	CN_D288	CN_D252
4	XPO6	CN_D318	CN_D298	CN_D252	CN_D318
4	XKR5	CN_D318	CN_D292	CN_D253.2	CN_D288
4	WWOX	CN_D318	CN_D296	CN_D252	CN_328
4	WWC2	CN_D318	CN_D288	CN_D252	CN_D225
4	WDTC1	CN_D318	CN_D292	CN_D252	CN_D292
4	WDFY3-AS2	CN_D318	CN_D292	CN_D252	CN_D225
4	WBP11	CN_D318	CN_D288	CN_D254	CN_D224
4	VRK1	CN_D292	CN_D254	CN_D252	CN_D254
4	VPS37A	CN_D318	CN_D292	CN_D288	CN_D252
4	UTRN	CN_D298	CN_D292	CN_D298	CN_D292
4	USP43	CN_D318	CN_D225	CN_D224	CN_D225
4	USO1	CN_D318	CN_D292	CN_D252	CN_D225
4	UROD	CN_D318	CN_D292	CN_D288	CN_D252
4	UNC79	CN_D300	CN_D254	CN_D252	CN_D254
4	UCP1	CN_D318	CN_D252	CN_D225	CN_D292
4	UBR4	CN_D318	CN_D292	CN_D252	CN_D296
4	UAP1	ZS.DNA24	ZS.DNA23_	ZS.DNA12	CN_328
4	TYRP1	CN_D292	CN_D252	CN_D225	CN_324
4	TYK2	ZS.DNA28	CN_D310	CN_D302.2	CN_D302.2
4	TXLNA	CN_D318	CN_D292	CN_D288	CN_D252
4	TUSC3	CN_D318	CN_D292	CN_D288	CN_D252
4	TTF1	CN_D302.2	CN_D292	CN_D225	CN_D225
4	TSPAN9	CN_D288	CN_D254	CN_D224	CN_D224
4	TSPAN5	CN_D318	CN_D292	CN_D252	CN_D225
4	TRPM6	CN_D292	CN_D225	CN_328	CN_328
4	TRPM1	CN_D302.2	CN_D300	CN_D252	CN_D302.2
4	TNK1	CN_D318	CN_D225	CN_D224	CN_D292
4	TNFRSF8	CN_D318	CN_D292	CN_D288	CN_D252
4	TNFRSF1B	CN_D318	CN_D292	CN_D288	CN_D252
4	TNFRSF13B	CN_D318	CN_D292	CN_D225	CN_D224
4	TNFRSF10C	CN_D318	CN_D292	CN_D288	CN_D252

4	TNFRSF10B	CN_D318	CN_D288	CN_D252	CN_D292
4	TMEM95	CN_D318	CN_D225	CN_D224	CN_D292
4	TMEM2	CN_D302.2	CN_D292	CN_D225	CN_D225
4	TMEM234	CN_D318	CN_D292	CN_D288	CN_D252
4	TMEM229B	CN_D292	CN_D252	CN_D292	CN_D254
4	TMEM200B	CN_D318	CN_D296	CN_D292	CN_D252
4	TMEM184C	CN_D318	CN_D292	CN_D252	CN_D225
4	TMEM170A	CN_D318	CN_D300	CN_D252	CN_328
4	TMEM150C	CN_D318	CN_D292	CN_D252	CN_D225
4	TMEM144	CN_D300	CN_D292	CN_D252	CN_D225
4	TMCO4	CN_D318	CN_D296	CN_D292	CN_D252
4	TLR2	CN_D318	CN_D292	CN_D252	CN_D225
4	TLL1	CN_D318	CN_D252	CN_D225	CN_D292
4	TKTL2	CN_D318	CN_D292	CN_D252	CN_D225
4	TIGD4	CN_D318	CN_D292	CN_D252	CN_D225
4	TIGD2	CN_D318	CN_D292	CN_D252	CN_D225
4	TIFA	CN_D318	CN_D292	CN_D252	CN_D225
4	THAP6	CN_D318	CN_D252	CN_D225	CN_D292
4	TFAP2E	CN_D318	CN_D292	CN_D288	CN_D252
4	TEKT3	CN_D318	CN_D292	CN_D225	CN_D224
4	TDO2	CN_D318	CN_D292	CN_D252	CN_D225
4	TBC1D9	CN_D318	CN_D252	CN_D225	CN_D292
4	TAS2R9	CN_D318	CN_D288	CN_D254	CN_D224
4	TAS2R8	CN_D318	CN_D288	CN_D254	CN_D224
4	TAS2R7	CN_D318	CN_D288	CN_D254	CN_D224
4	TAS2R13	CN_D318	CN_D288	CN_D254	CN_D224
4	TAS2R10	CN_D318	CN_D288	CN_D254	CN_D224
4	TACR3	CN_D292	CN_D225	CN_D224	CN_D292
4	SYT16	CN_D292	CN_D254	CN_D252	CN_D254
4	SYNPO2	CN_D318	CN_D292	CN_D252	CN_D225
4	SYNE2	CN_D292	CN_D254	CN_D252	CN_D254
4	SVEP1	CN_D302.2	CN_D292	CN_D225	CN_D292
4	STYK1	CN_D318	CN_D288	CN_D254	CN_D224
4	STX8	CN_D318	CN_D225	CN_D224	CN_D225
4	STRN3	CN_D292	CN_D254	CN_D292	CN_D254
4	STRBP	CN_D302.2	CN_D225	CN_D302.2	CN_D292
4	STBD1	CN_D318	CN_D292	CN_D252	CN_D225
4	ST3GAL3	CN_D318	CN_D292	CN_D252	CN_D292
4	SPRY1	CN_D318	CN_D292	CN_D252	CN_D225
4	SPOCK3	CN_D318	CN_D310	CN_D252	CN_D225
4	SPATA5	CN_D318	CN_D292	CN_D252	CN_D225
4	SPARCL1	CN_D318	CN_D292	CN_D252	CN_D225
4	SNRPD3	CN_D296	CN_D288	CN_D296	CN_D288

4	SNORD73A	CN_D318	CN_D292	CN_D252	CN_D225
4	SNORD65	CN_D318	CN_D292	CN_D225	CN_D224
4	SNORD49B	CN_D318	CN_D292	CN_D225	CN_D224
4	SNORD49A	CN_D318	CN_D292	CN_D225	CN_D224
4	SNCA	CN_D318	CN_D292	CN_D252	CN_D225
4	SMARCA5	CN_D318	CN_D252	CN_D225	CN_D292
4	SMAP2	CN_D318	CN_D292	CN_D288	CN_D252
4	SMAD1	CN_D318	CN_D292	CN_D252	CN_D225
4	SLFNL1	CN_D318	CN_D292	CN_D288	CN_D252
4	SLCO1C1	CN_D318	CN_D288	CN_D224	CN_D224
4	SLCO1A2	CN_D318	CN_D288	CN_D224	CN_324
4	SLC7A2	CN_D318	CN_D292	CN_D288	CN_D252
4	SLC7A11	CN_D318	CN_D292	CN_D252	CN_D225
4	SLC39A8	CN_D318	CN_D292	CN_D252	CN_D225
4	SLC39A11	ZS.DNA28	CN_D302.2	CN_D225	CN_D225
4	SLC24A4	CN_D254	CN_D252	CN_D292	CN_D252
4	SLC15A5	CN_D318	CN_D288	CN_D254	CN_D224
4	SLC10A7	CN_D318	CN_D292	CN_D252	CN_D225
4	SLC10A6	CN_D318	CN_D252	CN_D225	CN_D292
4	SHROOM3	CN_D318	CN_D292	CN_D252	CN_D225
4	SH3GLB2	CN_D302.2	CN_D292	CN_D253.2	CN_D225
4	SH3D19	CN_D318	CN_D292	CN_D252	CN_D225
4	SH2D4A	CN_D318	CN_D292	CN_D288	CN_D252
4	SGK3	ZS.DNA28	CN_D304	CN_D302.2	CN_D225
4	SGK223	CN_D318	CN_D292	CN_D288	CN_D253.2
4	SGK196	CN_D298	CN_D292	CN_D288	CN_D292
4	SFRP2	CN_D318	CN_D252	CN_D225	CN_D292
4	SFPQ	CN_D318	CN_D292	CN_D288	CN_D252
4	SETX	CN_D302.2	CN_D292	CN_D225	CN_D225
4	SETD7	CN_D318	CN_D292	CN_D225	CN_D252
4	SESN3	CN_D300	CN_D254	CN_328	CN_D254
4	SEPT1	CN_D318	CN_D304	CN_D294	CN_D252
4	SEC31A	CN_D318	CN_D292	CN_D252	CN_D225
4	SEC24B	CN_D318	CN_D292	CN_D252	CN_D225
4	SDAD1	CN_D318	CN_D292	CN_D252	CN_D225
4	SCMH1	CN_D318	CN_D292	CN_D288	CN_D252
4	SCLT1	CN_D318	CN_D292	CN_D252	CN_D225
4	SCD5	CN_D318	CN_D292	CN_D252	CN_D225
4	SCARNA8	CN_D292	CN_D253.2	CN_D252	CN_D225
4	SCARB2	CN_D318	CN_D252	CN_D225	CN_D292
4	SAMD4A	CN_D254	CN_D252	CN_D292	CN_D254
4	RXFP1	CN_D318	CN_D292	CN_D252	CN_D225
4	RUNX3	CN_D318	CN_D296	CN_D292	CN_D252

4	RRH	CN_D318	CN_D292	CN_D252	CN_D225
4	RRAGA	CN_D292	CN_D253.2	CN_D252	CN_D225
4	RPSAP9	CN_D302.2	CN_D292	CN_D253.2	CN_D225
4	RPS6KA2	CN_D292	CN_D254	CN_D292	CN_D254
4	RPS3A	CN_D318	CN_D292	CN_D252	CN_D225
4	RPL34	CN_D318	CN_D292	CN_D252	CN_D225
4	RPL13AP20	CN_D318	CN_D288	CN_D254	CN_D224
4	ROR2	CN_D302.2	CN_D292	CN_D225	CN_D302.2
4	RNF6	CN_D318	CN_D292	CN_D224	CN_D224
4	RNF186	CN_D318	CN_D296	CN_D292	CN_D252
4	RNF175	CN_D318	CN_D292	CN_D252	CN_D225
4	RNF112	CN_D318	CN_D292	CN_D225	CN_D224
4	RIN3	CN_D292	CN_D254	CN_D252	CN_D254
4	RHOBTB2	CN_D318	CN_D288	CN_D252	CN_D292
4	RHBDL2	CN_D318	CN_D292	CN_D288	CN_D252
4	RG9MTD2	CN_D318	CN_D292	CN_D252	CN_D225
4	RFK	CN_D302.2	CN_D292	CN_D253.2	CN_D225
4	RERG	CN_D318	CN_D288	CN_D254	CN_D224
4	RERE	CN_D318	CN_D292	CN_D252	CN_D292
4	RCVRN	CN_D318	CN_D292	CN_D225	CN_D224
4	RCHY1	CN_D318	CN_D252	CN_D225	CN_D292
4	RBM46	CN_D318	CN_D252	CN_D225	CN_D292
4	RANBP17	CN_D318	CN_D296	CN_324	CN_320
4	RABGAP1L	ZS.DNA9	ZS.DNA25	ZS.DNA24	ZS.DNA11
4	RABGAP1	CN_D302.2	CN_D292	CN_D225	CN_D292
4	RABEP1	CN_D318	CN_D292	CN_D225	CN_D224
4	PTPRU	CN_D318	CN_D296	CN_D292	CN_D252
4	PTPRO	CN_D318	CN_D288	CN_D254	CN_D224
4	PTPRK	ZS.DNA23_	CN_328	CN_324	CN_328
4	PTPN13	CN_D318	CN_D252	CN_D225	CN_D292
4	PTPLAD2	CN_D292	CN_D252	CN_D225	CN_D292
4	PTCH1	CN_D302.2	CN_D292	CN_D253.2	CN_D225
4	PSMB2	CN_D318	CN_D292	CN_D288	CN_D252
4	PSG9	ZS.DNA28	CN_D302.2	CN_D227	CN_D225
4	PSG5	ZS.DNA28	CN_D302.2	CN_D227	CN_D225
4	PSG4	ZS.DNA28	CN_D302.2	CN_D227	CN_D225
4	PRSS48	CN_D318	CN_D292	CN_D252	CN_D225
4	PRSS12	CN_D292	CN_D252	CN_D225	CN_D292
4	PRRX1	ZS.DNA26	ZS.DNA24	ZS.DNA23_	ZS.DNA11
4	PRRC2B	CN_D302.2	CN_D292	CN_D225	CN_D292
4	PRR4	CN_D318	CN_D288	CN_D254	CN_D224
4	PRMT8	CN_D288	CN_D254	CN_D224	CN_D224
4	PRMT10	CN_D318	CN_D292	CN_D252	CN_D225

4	PRKG2	CN_D318	CN_D292	CN_D252	CN_D225
4	PRKCH	CN_D292	CN_D254	CN_D252	CN_328
4	PRH1	CN_D318	CN_D288	CN_D254	CN_D224
4	PRDM5	CN_D318	CN_D292	CN_D252	CN_D225
4	PRDM2	CN_D318	CN_D296	CN_D292	CN_D252
4	PRB2	CN_D318	CN_D288	CN_D254	CN_D224
4	PQLC2	CN_D318	CN_D296	CN_D292	CN_D252
4	PPP3CA	CN_D318	CN_D292	CN_D252	CN_D225
4	PPP2R2A	CN_D318	CN_D292	CN_D288	CN_D252
4	PPP1R3B	CN_D318	CN_D292	CN_D253.2	CN_D288
4	PPP1R1A	ZS.DNA28	CN_D318	CN_D288	CN_D224
4	PPM1K	CN_D318	CN_D292	CN_D252	CN_D225
4	PPIE	CN_D318	CN_D292	CN_D288	CN_D252
4	PPID	CN_D318	CN_D292	CN_D252	CN_D225
4	PPEF2	CN_D318	CN_D292	CN_D252	CN_D225
4	PPBPL2	CN_D318	CN_D292	CN_D252	CN_D225
4	PPBP	CN_D318	CN_D252	CN_D225	CN_D292
4	PNMA2	CN_D318	CN_D292	CN_D288	CN_D252
4	PMFBP1	CN_D318	CN_D296	CN_D252	CN_328
4	PLIN2	CN_D292	CN_D253.2	CN_D252	CN_D225
4	PLCZ1	CN_D318	CN_D288	CN_324	CN_D224
4	PLCG2	CN_D318	CN_D300	CN_D296	CN_D252
4	PLBD1	CN_D318	CN_D288	CN_D254	CN_D224
4	PLAC8	CN_D318	CN_D292	CN_D252	CN_D225
4	PLA2G15	CN_D318	CN_D302.2	CN_D252	CN_D302.2
4	PLA2G12A	CN_D318	CN_D292	CN_D252	CN_D225
4	PKD2	CN_D318	CN_D252	CN_D225	CN_D292
4	PITX2	CN_D318	CN_D292	CN_D252	CN_D225
4	PINX1	CN_D318	CN_D292	CN_D288	CN_D292
4	PIGY	CN_D318	CN_D292	CN_D252	CN_D225
4	PHF17	CN_D318	CN_D292	CN_D252	CN_D225
4	PGRMC2	CN_D318	CN_D252	CN_D225	CN_D292
4	PGM5	CN_D302.2	CN_D292	CN_D225	CN_D292
4	PGD	CN_D318	CN_D252	CN_D296	CN_D292
4	PF4V1	CN_D318	CN_D292	CN_D252	CN_D225
4	PF4	CN_D318	CN_D252	CN_D225	CN_D292
4	PDPN	CN_D318	CN_D296	CN_D292	CN_D252
4	PDGFRL	CN_D318	CN_D292	CN_D288	CN_D252
4	PDE6H	CN_D318	CN_D288	CN_D254	CN_D224
4	PDE1B	ZS.DNA28	CN_D318	CN_D288	CN_D224
4	PDE10A	CN_D292	CN_D254	CN_D292	CN_D254
4	PCSK5	CN_D302.2	CN_D292	CN_D253.2	CN_D225
4	PCNX	CN_D292	CN_D254	CN_D252	CN_D254

4	PCDH18	CN_D318	CN_D292	CN_D252	CN_D225
4	PCDH15	CN_D296	CN_D225	CN_D296	CN_D225
4	PBX3	CN_D302.2	CN_D292	CN_D225	CN_D302.2
4	PARM1	CN_D318	CN_D292	CN_D252	CN_D225
4	PARK2	CN_D292	CN_D254	CN_D292	CN_D254
4	PALLD	CN_D318	CN_D310	CN_D252	CN_D225
4	PACRG	CN_D292	CN_D254	CN_D292	CN_D254
4	OXCT2	CN_D318	CN_D292	CN_D288	CN_D252
4	OTUD3	CN_D318	CN_D296	CN_D292	CN_D252
4	OSTC	CN_D318	CN_D292	CN_D252	CN_D225
4	OPRD1	CN_D318	CN_D296	CN_D292	CN_D252
4	OPCML	CN_D318	CN_D300	CN_D254	CN_D254
4	OLR1	CN_D318	CN_D288	CN_D254	CN_D224
4	OLFM3	CN_D318	CN_D292	CN_D252	CN_D292
4	ODZ2	CN_D318	CN_D296	CN_328	CN_324
4	NXPH1	ZS.DNA9	CN_328	CN_324	CN_328
4	NUP54	CN_D318	CN_D252	CN_D225	CN_D292
4	NUP214	CN_D302.2	CN_D292	CN_D225	CN_D292
4	NUDT9	CN_D318	CN_D292	CN_D252	CN_D225
4	NUDT6	CN_D318	CN_D292	CN_D252	CN_D225
4	NTN1	CN_D318	CN_D292	CN_D225	CN_D224
4	NTM	CN_D318	CN_D300	CN_D254	CN_D254
4	NT5C1A	CN_D318	CN_D292	CN_D288	CN_D252
4	NRXN3	CN_D254	CN_D252	CN_328	CN_D254
4	NR6A1	CN_D302.2	CN_D292	CN_D225	CN_D292
4	NR3C2	CN_D318	CN_D292	CN_D252	CN_D225
4	NPY5R	CN_D318	CN_D292	CN_D252	CN_D225
4	NPY1R	CN_D318	CN_D292	CN_D252	CN_D225
4	NPNT	CN_D318	CN_D292	CN_D252	CN_D225
4	NPM2	CN_D318	CN_D292	CN_D288	CN_D252
4	NPAS3	CN_D292	CN_D254	CN_D224	CN_D254
4	NOX3	CN_D292	CN_D254	CN_328	CN_D254
4	NINJ2	CN_D288	CN_D254	CN_D224	CN_D224
4	NIN	CN_D254	CN_D252	CN_D251	CN_D254
4	NFYC	CN_D318	CN_D292	CN_D288	CN_D252
4	NFKB1	CN_D318	CN_D252	CN_D225	CN_D224
4	NEUROG2	CN_D318	CN_D252	CN_D225	CN_D292
4	NEURL4	CN_D318	CN_D225	CN_D224	CN_D292
4	NELL1	CN_D318	CN_328	CN_324	CN_328
4	NEIL3	CN_D318	CN_D310	CN_D252	CN_D225
4	NEGR1-IT1	CN_D318	CN_D288	CN_D252	CN_328
4	NDUFS5	CN_D318	CN_D292	CN_D288	CN_D252
4	NDUFC1	CN_D318	CN_D292	CN_D252	CN_D225

4	NCKAP1L	ZS.DNA28	CN_D318	CN_D288	CN_D224
4	NCDN	CN_D318	CN_D292	CN_D288	CN_D252
4	NCAPD3	CN_D318	CN_D300	CN_D254	CN_D254
4	NBPF3	CN_D318	CN_D296	CN_D292	CN_D252
4	NBLA00301	CN_D318	CN_D310	CN_D252	CN_D225
4	NAT2	CN_D318	CN_D292	CN_D288	CN_D252
4	NAT1	CN_D318	CN_D292	CN_D288	CN_D252
4	NAP1L5	CN_D318	CN_D292	CN_D252	CN_D225
4	NAAA	CN_D318	CN_D292	CN_D252	CN_D225
4	NAA15	CN_D318	CN_D292	CN_D252	CN_D225
4	MYOZ2	CN_D318	CN_D252	CN_D225	CN_D292
4	MYOM3	CN_D318	CN_D292	CN_D252	CN_D292
4	MYO18B	CN_D296	CN_D288	CN_D296	CN_D288
4	MYH13	CN_D318	CN_D292	CN_D225	CN_D224
4	MYH11	ZS.DNA28	CN_D318	CN_D298	CN_D252
4	MYH10	CN_D318	CN_D225	CN_D224	CN_D224
4	MTUS1	CN_D318	CN_D292	CN_D288	CN_D252
4	MTTP	CN_D318	CN_D292	CN_D252	CN_D225
4	MTMR9LP	CN_D318	CN_D292	CN_D288	CN_D252
4	MTMR7	CN_D318	CN_D292	CN_D288	CN_D252
4	MTHFD2L	CN_D318	CN_D292	CN_D252	CN_D225
4	MTAP	CN_D292	CN_D252	CN_D225	CN_D225
4	MSRA	CN_D318	CN_D292	CN_D288	CN_D253.2
4	MSMO1	CN_D318	CN_D292	CN_D252	CN_D225
4	MRTO4	CN_D318	CN_D296	CN_D292	CN_D252
4	MRPS18C	CN_D318	CN_D292	CN_D252	CN_D225
4	MRPS15	CN_D318	CN_D292	CN_D288	CN_D252
4	MPZL1	ZS.DNA23_	ZS.DNA11	CN_328	CN_324
4	MPDZ	CN_D252	CN_D225	CN_324	CN_D225
4	MND1	CN_D318	CN_D292	CN_D252	CN_D225
4	MMRN1	CN_D318	CN_D292	CN_D252	CN_D225
4	MMAA	CN_D318	CN_D292	CN_D252	CN_D225
4	MIR614	CN_D318	CN_D288	CN_D254	CN_D224
4	MIR578	CN_D318	CN_D292	CN_D252	CN_D225
4	MIR575	CN_D318	CN_D292	CN_D252	CN_D225
4	MIR548H3	CN_D302.2	CN_D292	CN_D253.2	CN_D225
4	MIR4799	CN_D318	CN_D292	CN_D252	CN_D225
4	MIR4708	CN_D254	CN_D252	CN_328	CN_D292
4	MIR4660	CN_D318	CN_D292	CN_D288	CN_D253.2
4	MIR4659B	CN_D318	CN_D292	CN_D288	CN_D253.2
4	MIR4659A	CN_D318	CN_D292	CN_D288	CN_D253.2
4	MIR4632	CN_D318	CN_D292	CN_D288	CN_D252
4	MIR4287	CN_D318	CN_D292	CN_D288	CN_D252

4	MIR4253	CN_D318	CN_D292	CN_D288	CN_D252
4	MIR4251	CN_D318	CN_D292	CN_D252	CN_D296
4	MIR367	CN_D318	CN_D292	CN_D252	CN_D225
4	MIR31HG	CN_D292	CN_D252	CN_D251	CN_D225
4	MIR3134	CN_D302.2	CN_D292	CN_D225	CN_D292
4	MIR3115	CN_D318	CN_D292	CN_D288	CN_D252
4	MIR30E	CN_D318	CN_D292	CN_D288	CN_D252
4	MIR30C1	CN_D318	CN_D292	CN_D288	CN_D252
4	MIR302D	CN_D318	CN_D292	CN_D252	CN_D225
4	MIR302C	CN_D318	CN_D292	CN_D252	CN_D225
4	MIR302B	CN_D318	CN_D292	CN_D252	CN_D225
4	MIR302A	CN_D318	CN_D292	CN_D252	CN_D225
4	MIR204	CN_D302.2	CN_D292	CN_D253.2	CN_D225
4	MIR1244-3	CN_D318	CN_D288	CN_D224	CN_D254
4	MIR1244-2	CN_D318	CN_D288	CN_D224	CN_D254
4	MIR1244-1	CN_D318	CN_D288	CN_D224	CN_D254
4	MIR1180	CN_D318	CN_D225	CN_D224	CN_D292
4	MIPOL1	CN_D292	CN_D254	CN_D252	CN_D292
4	MGST2	CN_D318	CN_D292	CN_D252	CN_D225
4	MGP	CN_D318	CN_D288	CN_D254	CN_D224
4	MGC12916	CN_D318	CN_D292	CN_D225	CN_D224
4	MFHAS1	CN_D318	CN_D292	CN_D288	CN_D253.2
4	MFAP4	CN_D318	CN_D292	CN_D225	CN_D224
4	MEFV	CN_D318	CN_D300	CN_D298	CN_D252
4	MECR	CN_D318	CN_D296	CN_D292	CN_D252
4	MBOAT4	CN_D318	CN_D292	CN_D288	CN_D252
4	MAP9	CN_D318	CN_D292	CN_D252	CN_D225
4	MAP3K9	CN_D292	CN_D254	CN_D252	CN_D292
4	MAP2K4	CN_D318	CN_D292	CN_D225	CN_D224
4	MANBA	CN_D318	CN_D252	CN_D225	CN_D224
4	MAN1C1	CN_D318	CN_D292	CN_D252	CN_D292
4	MAML3	CN_D318	CN_D292	CN_D252	CN_D225
4	MAD2L2	CN_D318	CN_D292	CN_D288	CN_D252
4	MACF1	CN_D318	CN_D292	CN_D288	CN_D252
4	MAB21L2	CN_D318	CN_D292	CN_D252	CN_D225
4	LZTS1	CN_D318	CN_D292	CN_D288	CN_D252
4	LUZP1	CN_D318	CN_D292	CN_D288	CN_D252
4	LURAP1L	CN_D292	CN_D252	CN_D225	CN_324
4	LTBP2	CN_D254	CN_D252	CN_D251	CN_D254
4	LSM6	CN_D318	CN_D292	CN_D252	CN_D225
4	LRP6	CN_D318	CN_D288	CN_D224	CN_D254
4	LRIT3	CN_D318	CN_D292	CN_D252	CN_D225
4	LRAT	CN_D318	CN_D252	CN_D225	CN_D292

4	LPPR1	CN_D292	CN_D225	CN_328	CN_D292
4	LPA	CN_D292	CN_D254	CN_328	CN_D254
4	LOXHD1	CN_D318	CN_D298	CN_D288	CN_D288
4	LOH12CR1	CN_D318	CN_D288	CN_D254	CN_D224
4	LOC729059	CN_D318	CN_D292	CN_D288	CN_D252
4	LOC653160	CN_D318	CN_D292	CN_D288	CN_D252
4	LOC647946	CN_D318	CN_D288	CN_328	CN_328
4	LOC645431	CN_D254	CN_D252	CN_328	CN_D292
4	LOC644248	CN_D318	CN_D292	CN_D252	CN_D225
4	LOC641518	CN_D318	CN_D292	CN_D252	CN_D225
4	LOC641365	CN_D318	CN_D292	CN_D252	CN_D225
4	LOC641364	CN_D318	CN_D292	CN_D252	CN_D225
4	LOC441025	CN_D318	CN_D292	CN_D252	CN_D225
4	LOC400752	CN_D318	CN_D292	CN_D288	CN_D252
4	LOC339166	CN_D318	CN_D292	CN_D225	CN_D224
4	LOC338817	CN_D318	CN_D288	CN_D254	CN_D224
4	LOC286059	CN_D318	CN_D292	CN_D288	CN_D252
4	LOC285456	CN_D318	CN_D292	CN_D252	CN_D225
4	LOC285419	CN_D318	CN_D292	CN_D252	CN_D225
4	LOC284344	ZS.DNA28	CN_D302.2	CN_D227	CN_D225
4	LOC256880	CN_D318	CN_D292	CN_D252	CN_D225
4	LOC254896	CN_D318	CN_D292	CN_D288	CN_D252
4	LOC157273	CN_D318	CN_D292	CN_D288	CN_D253.2
4	LOC100652791	CN_D318	CN_D292	CN_D288	CN_D253.2
4	LOC100507178	CN_D318	CN_D292	CN_D288	CN_D252
4	LOC100507053	CN_D318	CN_D292	CN_D252	CN_D225
4	LOC100506746	CN_D318	CN_D292	CN_D252	CN_D225
4	LOC100506730	CN_D318	CN_D296	CN_D252	CN_D292
4	LOC100506314	CN_D318	CN_D288	CN_D254	CN_D224
4	LOC100506229	CN_D318	CN_D310	CN_D288	CN_D252
4	LOC100506190	CN_D292	CN_D225	CN_D302.2	CN_D225
4	LOC100506172	CN_D318	CN_D302.2	CN_D252	CN_D253.2
4	LOC100506035	CN_D318	CN_D292	CN_D252	CN_D225
4	LOC100505989	CN_D318	CN_D252	CN_D225	CN_D292
4	LOC100505702	CN_D318	CN_D252	CN_D225	CN_D292
4	LOC100505545	CN_D318	CN_D292	CN_D252	CN_D225
4	LOC100499177	CN_D318	CN_D252	CN_D225	CN_D292
4	LOC100292680	CN_D253.2	CN_D224	CN_D288	CN_D254
4	LOC100287015	CN_D318	CN_D292	CN_D288	CN_D253.2
4	LOC100128993	CN_D318	CN_D292	CN_D288	CN_D252
4	LOC100128787	CN_D318	CN_D292	CN_D252	CN_328
4	LMX1B	CN_D302.2	CN_D292	CN_D225	CN_D292
4	LMO3	CN_D318	CN_D288	CN_D254	CN_D224

4	LINC00575	CN_D318	CN_D292	CN_D225	CN_D252
4	LINC00339	CN_D318	CN_D296	CN_D292	CN_D252
4	LEPROTL1	CN_D318	CN_D292	CN_D288	CN_D252
4	LEF1	CN_D318	CN_D292	CN_D252	CN_D225
4	LDLRAP1	CN_D318	CN_D252	CN_D296	CN_D292
4	LARP7	CN_D318	CN_D292	CN_D252	CN_D225
4	LARP1B	CN_D318	CN_D292	CN_D225	CN_D292
4	LAMTOR3	CN_D318	CN_D292	CN_D252	CN_D225
4	KRT16P2	CN_D318	CN_D292	CN_D225	CN_D224
4	KPNA6	CN_D318	CN_D292	CN_D288	CN_D252
4	KLRK1	CN_D318	CN_D254	CN_D224	CN_D288
4	KLRD1	CN_D318	CN_D254	CN_D224	CN_D288
4	KLRC4	CN_D318	CN_D288	CN_D254	CN_D224
4	KLRC3	CN_D318	CN_D288	CN_D254	CN_D224
4	KLRC2	CN_D318	CN_D288	CN_D254	CN_D224
4	KLRC1	CN_D318	CN_D288	CN_D254	CN_D224
4	KLRAP1	CN_D318	CN_D254	CN_D224	CN_D288
4	KLHL9	CN_D292	CN_D252	CN_D251	CN_D225
4	KLHL8	CN_D318	CN_D292	CN_D252	CN_D225
4	KLHL2	CN_D318	CN_D292	CN_D252	CN_D225
4	KLHDC7A	CN_D318	CN_D296	CN_D292	CN_D252
4	KIRREL3	CN_D318	CN_D300	CN_D254	CN_328
4	KIF13B	CN_D318	CN_D292	CN_D288	CN_D252
4	KIAA1958	CN_D302.2	CN_D292	CN_D225	CN_D292
4	KIAA1467	CN_D318	CN_D288	CN_D254	CN_D224
4	KIAA1456	CN_D318	CN_D292	CN_D288	CN_D292
4	KIAA1324	CN_D318	CN_D292	CN_D252	CN_D318
4	KIAA0825	CN_D318	CN_D296	CN_328	CN_324
4	KIAA0319L	CN_D318	CN_D292	CN_D288	CN_D252
4	KIAA0090	CN_D318	CN_D292	CN_D252	CN_D296
4	KDM5A	CN_D288	CN_D254	CN_D224	CN_D224
4	KDM1A	CN_D318	CN_D292	CN_D288	CN_D252
4	KCTD11	CN_D318	CN_D225	CN_D224	CN_D292
4	KCNU1	CN_D318	CN_D292	CN_D288	CN_328
4	KCNQ4	CN_D318	CN_D292	CN_D288	CN_D252
4	KAZN	CN_D318	CN_D296	CN_D292	CN_D252
4	KANSL1	CN_D304	CN_D225	CN_D224	CN_D225
4	KANK1	CN_D298	CN_D292	CN_D225	CN_D225
4	JAM3	CN_D318	CN_D300	CN_D254	CN_D254
4	IQSEC3	CN_D288	CN_D254	CN_D224	CN_D288
4	IQCC	CN_D318	CN_D292	CN_D288	CN_D252
4	IPCEF1	CN_D292	CN_D254	CN_D224	CN_D254
4	IL15	CN_D318	CN_D292	CN_D252	CN_D225

4	IKBKAP	CN_D302.2	CN_D292	CN_D225	CN_D292
4	IGSF21	CN_D318	CN_D296	CN_D292	CN_D252
4	IGFBP7	CN_D300	CN_D292	CN_D225	CN_D292
4	IFNE	CN_D292	CN_D252	CN_D251	CN_D225
4	IFNA8	CN_D292	CN_D252	CN_D251	CN_D225
4	IFNA6	CN_D292	CN_D252	CN_D251	CN_D225
4	IFNA2	CN_D292	CN_D252	CN_D251	CN_D225
4	IFNA1	CN_D292	CN_D252	CN_D251	CN_D225
4	IFNA13	CN_D292	CN_D252	CN_D251	CN_D225
4	IFFO2	CN_D318	CN_D296	CN_D292	CN_D252
4	HTR7P1	CN_D318	CN_D288	CN_D254	CN_D224
4	HTR1D	CN_D318	CN_D292	CN_D288	CN_D252
4	HSPG2	CN_D318	CN_D292	CN_D252	CN_D292
4	HSP90AA4P	CN_D288	CN_D252	CN_D225	CN_D292
4	HSDL2	CN_D302.2	CN_D292	CN_D225	CN_D292
4	HSD17B13	CN_D318	CN_D292	CN_D252	CN_D225
4	HSD17B11	CN_D318	CN_D292	CN_D252	CN_D225
4	HS3ST3B1	CN_D318	CN_D292	CN_D225	CN_D224
4	HS3ST3A1	CN_D318	CN_D292	CN_D225	CN_D224
4	HPSE	CN_D318	CN_D292	CN_D252	CN_D225
4	HPR	CN_D318	CN_D300	CN_D296	CN_D252
4	HP	CN_D318	CN_D300	CN_D296	CN_D252
4	HPCAL4	CN_D318	CN_D292	CN_D288	CN_D252
4	HOOK3	CN_D298	CN_D292	CN_D288	CN_D292
4	HNRPDL	CN_D318	CN_D292	CN_D252	CN_D225
4	HNRNPD	CN_D318	CN_D292	CN_D252	CN_D225
4	HIVEP3	CN_D318	CN_D292	CN_D252	CN_D292
4	HEYL	CN_D318	CN_D292	CN_D288	CN_D252
4	HERC6	CN_D318	CN_D292	CN_D252	CN_D225
4	HERC5	CN_D318	CN_D292	CN_D252	CN_D225
4	HERC3	CN_D318	CN_D292	CN_D252	CN_D225
4	HELT	CN_D318	CN_D310	CN_D288	CN_D252
4	HELQ	CN_D318	CN_D292	CN_D252	CN_D225
4	HECTD3	CN_D318	CN_D292	CN_D288	CN_D252
4	HEBP1	CN_D318	CN_D288	CN_D254	CN_D224
4	HAUS6	CN_D292	CN_D253.2	CN_D252	CN_D225
4	HAND2	CN_D318	CN_D310	CN_D252	CN_D225
4	HADH	CN_D318	CN_D292	CN_D252	CN_D225
4	H2AFZ	CN_D318	CN_D292	CN_D252	CN_D225
4	GUCY2C	CN_D318	CN_D288	CN_D224	CN_D224
4	GUCY1B3	CN_D318	CN_D292	CN_D252	CN_D225
4	GUCY1A3	CN_D318	CN_D292	CN_D252	CN_D225
4	GTSF1	ZS.DNA28	CN_D318	CN_D288	CN_D224

4	GSTCD	CN_D318	CN_D292	CN_D252	CN_D225
4	GSG1	CN_D318	CN_D288	CN_D254	CN_D224
4	GRID2	CN_D318	CN_D300	CN_D252	CN_D225
4	GRAMD1B	CN_D318	CN_D300	CN_D254	CN_D254
4	GPS2	CN_D318	CN_D225	CN_D224	CN_D292
4	GPRIN3	CN_D318	CN_D292	CN_D252	CN_D225
4	GPRC5D	CN_D318	CN_D288	CN_D254	CN_D224
4	GPRC5A	CN_D318	CN_D288	CN_D254	CN_D224
4	GPR88	CN_D318	CN_D292	CN_D252	CN_328
4	GPR19	CN_D318	CN_D288	CN_D224	CN_D254
4	GNRH1	CN_D318	CN_D292	CN_D288	CN_D252
4	GNAI3	CN_D318	CN_D292	CN_D252	CN_D292
4	GLTSCR1	ZS.DNA28	CN_D306	CN_D292	CN_320
4	GLRA3	CN_D310	CN_D292	CN_D252	CN_D225
4	GGT1	CN_D296	CN_D288	CN_D296	CN_D288
4	GFRA2	CN_D318	CN_D292	CN_D288	CN_D252
4	GCNT1	CN_D302.2	CN_D253.2	CN_D225	CN_D292
4	GAR1	CN_D318	CN_D292	CN_D252	CN_D225
4	GAN	CN_D300	CN_D296	CN_D252	CN_328
4	GALNT7	CN_D318	CN_D310	CN_D292	CN_D225
4	GABRB2	CN_D318	CN_D296	CN_328	CN_324
4	GABARAPL1	CN_D318	CN_D288	CN_D254	CN_D224
4	GAB1	CN_D318	CN_D252	CN_D225	CN_D292
4	G3BP2	CN_D318	CN_D252	CN_D225	CN_D292
4	FUT8	CN_D254	CN_D252	CN_328	CN_D292
4	FUT10	CN_D292	CN_D288	CN_D252	CN_328
4	FSTL5	CN_D318	CN_D292	CN_D252	CN_D225
4	FRMD3	CN_D302.2	CN_D292	CN_D225	CN_D302.2
4	FREM3	CN_D318	CN_D292	CN_D252	CN_D225
4	FNIP2	CN_D318	CN_D292	CN_D252	CN_D225
4	FNBP1	CN_D302.2	CN_D292	CN_D225	CN_D292
4	FLRT2	CN_D254	CN_D252	CN_D254	CN_328
4	FLJ41200	CN_D292	CN_D252	CN_D225	CN_324
4	FLJ39639	CN_D318	CN_D300	CN_D298	CN_D252
4	FLJ34690	CN_D318	CN_D296	CN_D292	CN_D224
4	FHAD1	CN_D318	CN_D302.2	CN_D292	CN_D252
4	FGL1	CN_D318	CN_D292	CN_D288	CN_D252
4	FGF2	CN_D318	CN_D292	CN_D252	CN_D225
4	FGF20	CN_D318	CN_D292	CN_D288	CN_D252
4	FGF17	CN_D318	CN_D292	CN_D288	CN_D252
4	FGF14	CN_D310	CN_D298	CN_D292	CN_328
4	FER	CN_D318	CN_D296	CN_324	CN_324
4	FDCSP	CN_D292	CN_D252	CN_D225	CN_328

4	FBXO6	CN_D318	CN_D292	CN_D288	CN_D252
4	FBXO44	CN_D292	CN_D288	CN_D252	CN_D318
4	FBXL14	CN_D288	CN_D253.2	CN_D224	CN_D254
4	FANCC	CN_D302.2	CN_D292	CN_D225	CN_D292
4	FAM73B	CN_D302.2	CN_D292	CN_D253.2	CN_D225
4	FAM47E-STBD1	CN_D318	CN_D292	CN_D252	CN_D225
4	FAM211A	CN_D318	CN_D292	CN_D225	CN_D224
4	FAM198B	CN_D300	CN_D292	CN_D252	CN_D225
4	FAM175A	CN_D318	CN_D292	CN_D252	CN_D225
4	FAM167B	CN_D318	CN_D292	CN_D288	CN_D252
4	FAM154A	CN_D292	CN_D253.2	CN_D252	CN_D225
4	FAM13A	CN_D318	CN_D292	CN_D252	CN_D225
4	FAM13A-AS1	CN_D318	CN_D292	CN_D252	CN_D225
4	FAM129B	CN_D292	CN_D225	CN_D302.2	CN_D292
4	FAM108B1	CN_D302.2	CN_D292	CN_D225	CN_328
4	ETFDH	CN_D318	CN_D292	CN_D252	CN_D225
4	ERP27	CN_D318	CN_D288	CN_D254	CN_D224
4	EREG	CN_D318	CN_D292	CN_D252	CN_D225
4	EPS8	CN_D318	CN_D288	CN_D254	CN_D224
4	EPN2	CN_D318	CN_D225	CN_D224	CN_D292
4	EPHX2	CN_D292	CN_D288	CN_D252	CN_D292
4	EPGN	CN_D318	CN_D292	CN_D252	CN_D225
4	EPB49	CN_D318	CN_D292	CN_D288	CN_D252
4	EPB41	CN_D318	CN_D296	CN_D292	CN_D252
4	ENTPD4	CN_D318	CN_D292	CN_D288	CN_D252
4	ENPEP	CN_D318	CN_D292	CN_D252	CN_D225
4	ENOX1	CN_D318	CN_D298	CN_D292	CN_D292
4	ENOPH1	CN_D318	CN_D292	CN_D252	CN_D225
4	EMP1	CN_D318	CN_D288	CN_D224	CN_D224
4	ELMOD2	CN_D318	CN_D252	CN_D225	CN_D292
4	ELF2	CN_D318	CN_D292	CN_D252	CN_D225
4	EIF5A	CN_D318	CN_D225	CN_D224	CN_D292
4	EIF3I	CN_D318	CN_D292	CN_D288	CN_D252
4	EIF2B3	CN_D318	CN_D292	CN_D288	CN_D252
4	EGF	CN_D318	CN_D292	CN_D252	CN_D225
4	EFCAB4B	CN_D288	CN_D254	CN_D224	CN_D224
4	EDIL3	CN_D296	CN_328	CN_324	CN_328
4	EBF2	CN_D318	CN_D292	CN_D288	CN_D252
4	DUSP4	CN_D318	CN_D292	CN_D252	CN_D288
4	DUSP16	CN_D318	CN_D288	CN_D254	CN_D224
4	DSPP	CN_D318	CN_D292	CN_D252	CN_D225
4	DPP10	CN_D318	CN_D254	CN_328	CN_D254
4	DOLPP1	CN_D302.2	CN_D292	CN_D253.2	CN_D225

4	DOK2	CN_D318	CN_D292	CN_D288	CN_D252
4	DOCK8	CN_D292	CN_D225	CN_D292	CN_D253.2
4	DNAJB14	CN_D318	CN_D292	CN_D252	CN_D225
4	DNAH2	CN_D318	CN_D225	CN_D224	CN_D224
4	DMP1	CN_D318	CN_D252	CN_D225	CN_D292
4	DLGAP3	CN_D318	CN_D292	CN_D288	CN_D252
4	DLG2	ZS.DNA9	CN_D300	CN_328	CN_320
4	DKK2	CN_D318	CN_D292	CN_D252	CN_D225
4	DHRS3	CN_D318	CN_D296	CN_D292	CN_D252
4	DERA	CN_D318	CN_D288	CN_D254	CN_D224
4	DENND1B	ZS.DNA25	ZS.DNA11	CN_328	CN_324
4	DEFA6	CN_D318	CN_D292	CN_D288	CN_D253.2
4	DEFA4	CN_D318	CN_D292	CN_D288	CN_D253.2
4	DEFA3	CN_D318	CN_D288	CN_D292	CN_D253.2
4	DEFA1	CN_D318	CN_D288	CN_D292	CN_D253.2
4	DEFA1B	CN_D318	CN_D288	CN_D292	CN_D253.2
4	DCTN6	CN_D318	CN_D292	CN_D288	CN_D252
4	DCLK2	CN_D292	CN_D252	CN_D225	CN_D225
4	DCDC2B	CN_D318	CN_D292	CN_D288	CN_D252
4	DAPP1	CN_D318	CN_D292	CN_D252	CN_D225
4	DAPK1	CN_D302.2	CN_D292	CN_D225	CN_D292
4	CYP2U1	CN_D318	CN_D292	CN_D252	CN_D225
4	CXCL9	CN_D318	CN_D292	CN_D252	CN_D225
4	CXCL6	CN_D318	CN_D292	CN_D252	CN_D225
4	CXCL5	CN_D318	CN_D252	CN_D225	CN_D292
4	CXCL2	CN_D318	CN_D292	CN_D252	CN_D225
4	CXCL1	CN_D318	CN_D292	CN_D252	CN_D225
4	CXCL11	CN_D318	CN_D292	CN_D252	CN_D225
4	CXCL10	CN_D318	CN_D292	CN_D252	CN_D225
4	CUX2	CN_D318	CN_D294	CN_D288	CN_D252
4	CTSO	CN_D318	CN_D292	CN_D252	CN_D225
4	CTPS	CN_D318	CN_D292	CN_D288	CN_D252
4	CTNNAL1	CN_D302.2	CN_D292	CN_D225	CN_D292
4	CSMD2	CN_D318	CN_D292	CN_D288	CN_D252
4	CSF3R	CN_D318	CN_D292	CN_D288	CN_D252
4	CSDA	CN_D318	CN_D288	CN_D254	CN_D224
4	CREBL2	CN_D318	CN_D288	CN_D254	CN_D224
4	CPE	CN_D318	CN_D292	CN_D252	CN_D225
4	COQ2	CN_D318	CN_D292	CN_D252	CN_D225
4	COPS4	CN_D318	CN_D292	CN_D252	CN_D225
4	COL9A2	CN_D318	CN_D292	CN_D288	CN_D252
4	CNTNAP5	CN_D318	CN_D254	CN_328	CN_D254
4	CNOT7	CN_D318	CN_D292	CN_D288	CN_D252

4	CMIP	CN_D318	CN_D296	CN_D252	CN_328
4	CLSPN	CN_D318	CN_D292	CN_D288	CN_D252
4	CLGN	CN_D318	CN_D252	CN_D225	CN_D292
4	CLEC7A	CN_D318	CN_D288	CN_D254	CN_D224
4	CLEC2A	CN_D288	CN_D254	CN_D224	CN_D254
4	CLEC1A	CN_D318	CN_D288	CN_D254	CN_D224
4	CLEC12B	CN_D318	CN_D288	CN_D254	CN_D224
4	CITED4	CN_D318	CN_D292	CN_D288	CN_D252
4	CHRNA2	CN_D292	CN_D288	CN_D252	CN_D292
4	CFI	CN_D318	CN_D292	CN_D252	CN_D225
4	CFDP1	CN_D318	CN_D300	CN_D252	CN_328
4	CELA3B	CN_D318	CN_D296	CN_D292	CN_D252
4	CELA3A	CN_D318	CN_D296	CN_D292	CN_D252
4	CDYL2	CN_D318	CN_D302.2	CN_D252	CN_328
4	CDS1	CN_D318	CN_D292	CN_D252	CN_D225
4	CDRT15P1	CN_D296	CN_D292	CN_D225	CN_D224
4	CDRT15	CN_D318	CN_D292	CN_D225	CN_D224
4	CDKN2A	CN_D252	CN_324	CN_D292	CN_D225
4	CDKN1B	CN_D318	CN_D288	CN_D254	CN_D224
4	CDKL2	CN_D318	CN_D252	CN_D225	CN_D292
4	CDCA2	CN_D318	CN_D288	CN_D252	CN_D292
4	CDC42	CN_D318	CN_D296	CN_D292	CN_D252
4	CDC14A	CN_D318	CN_D292	CN_D252	CN_328
4	CCRN4L	CN_D318	CN_D292	CN_D252	CN_D225
4	CCNG2	CN_D318	CN_D292	CN_D252	CN_D225
4	CCDC85C	CN_D292	CN_D254	CN_D252	CN_D254
4	CCDC28B	CN_D318	CN_D292	CN_D288	CN_D252
4	CCDC158	CN_D318	CN_D292	CN_D252	CN_D225
4	CCDC109B	CN_D318	CN_D292	CN_D252	CN_D225
4	CASP6	CN_D318	CN_D292	CN_D252	CN_D225
4	CAMK2D	CN_D318	CN_D292	CN_D252	CN_D225
4	CADM1	CN_D318	CN_D300	CN_D254	CN_D254
4	C9orf93	CN_D292	CN_D252	CN_D225	CN_324
4	C9orf53	CN_D252	CN_324	CN_D292	CN_D225
4	C9orf4	CN_D302.2	CN_D292	CN_D225	CN_D292
4	C8orf75	CN_D318	CN_D292	CN_D288	CN_D252
4	C8orf48	CN_D318	CN_D292	CN_D288	CN_328
4	C8orf45	ZS.DNA28	CN_D304	CN_D302.2	CN_D225
4	C8orf44-SGK3	ZS.DNA28	CN_D304	CN_D302.2	CN_D225
4	C4orf49	CN_D318	CN_D292	CN_D252	CN_D225
4	C4orf46	CN_D318	CN_D292	CN_D252	CN_D225
4	C4orf43	CN_D318	CN_D292	CN_D252	CN_D225
4	C4orf36	CN_D318	CN_D292	CN_D252	CN_D225

4	C4orf26	CN_D318	CN_D252	CN_D225	CN_D292
4	C4orf22	CN_D318	CN_D292	CN_D252	CN_D225
4	C4orf17	CN_D318	CN_D292	CN_D252	CN_D225
4	C21orf7	CN_D300	CN_D254	CN_D300	CN_328
4	C1orf94	CN_D318	CN_D292	CN_D252	CN_D292
4	C1orf216	CN_D318	CN_D292	CN_D288	CN_D252
4	C1orf187	CN_D318	CN_D292	CN_D288	CN_D252
4	C17orf76-AS1	CN_D318	CN_D292	CN_D225	CN_D224
4	C12orf69	CN_D318	CN_D288	CN_D254	CN_D224
4	C12orf60	CN_D318	CN_D288	CN_D254	CN_D224
4	C12orf59	CN_D318	CN_D288	CN_D254	CN_D224
4	C12orf36	CN_D318	CN_D288	CN_D254	CN_D224
4	BNIP3L	CN_D318	CN_D292	CN_D288	CN_D252
4	BMP8B	CN_D318	CN_D292	CN_D288	CN_D252
4	BMP3	CN_D318	CN_D292	CN_D252	CN_D225
4	BCMO1	CN_D300	CN_D296	CN_D252	CN_328
4	BCL2L14	CN_D318	CN_D288	CN_D224	CN_D254
4	BANK1	CN_D318	CN_D292	CN_D252	CN_D225
4	B9D1	CN_D318	CN_D225	CN_D224	CN_D292
4	ATF7IP	CN_D318	CN_D288	CN_D254	CN_D224
4	ASAH1	CN_D318	CN_D292	CN_D288	CN_D252
4	ART3	CN_D318	CN_D292	CN_D252	CN_D225
4	ARSJ	CN_D318	CN_D292	CN_D252	CN_D225
4	ARHGEF10	CN_D318	CN_D292	CN_D288	CN_D288
4	ARHGDIB	CN_D318	CN_D288	CN_D254	CN_D224
4	ARHGAP44	CN_D318	CN_D292	CN_D224	CN_D292
4	AREG	CN_D318	CN_D292	CN_D252	CN_D225
4	AQR	CN_D300	CN_D252	CN_324	CN_D300
4	APOLD1	CN_D318	CN_D288	CN_D254	CN_D224
4	APITD1-CORT	CN_D318	CN_D252	CN_D296	CN_D292
4	APITD1	CN_D318	CN_D252	CN_D296	CN_D292
4	AP1AR	CN_D318	CN_D292	CN_D252	CN_D225
4	ANO2	CN_D288	CN_D254	CN_D224	CN_D254
4	ANKRD20A11P	CN_D300	CN_D254	CN_330	CN_D300
4	ANKRD17	CN_D292	CN_D252	CN_D225	CN_D292
4	ALPK1	CN_D318	CN_D292	CN_D252	CN_D225
4	ALG9	CN_D318	CN_D300	CN_D254	CN_D300
4	AKR7A3	CN_D318	CN_D296	CN_D252	CN_D292
4	AKR7A2	CN_D318	CN_D296	CN_D292	CN_D252
4	AKIRIN1	CN_D318	CN_D292	CN_D288	CN_D252
4	AKAP2	CN_D292	CN_D225	CN_D302.2	CN_D292
4	AHDC1	CN_D318	CN_D292	CN_D252	CN_D252
4	AGXT2L1	CN_D318	CN_D292	CN_D252	CN_D225

4	AGTRAP	CN_D318	CN_D292	CN_D288	CN_D252
4	AGPAT9	CN_D318	CN_D292	CN_D252	CN_D225
4	AGPAT5	CN_D318	CN_D292	CN_D288	CN_D253.2
4	AFF1	CN_D318	CN_D292	CN_D252	CN_D225
4	ADRA1A	CN_D318	CN_D292	CN_D288	CN_D252
4	ADK	CN_D296	CN_D225	CN_D296	CN_D225
4	ADH7	CN_D318	CN_D292	CN_D252	CN_D225
4	ADH6	CN_D318	CN_D292	CN_D252	CN_D225
4	ADH1C	CN_D318	CN_D292	CN_D252	CN_D225
4	ADH1B	CN_D318	CN_D292	CN_D252	CN_D225
4	ADCY10	ZS.DNA23_	ZS.DNA11	CN_328	CN_324
4	ADAMTSL1	CN_D292	CN_D252	CN_D225	CN_D292
4	ADAMTS3	CN_D292	CN_D252	CN_D225	CN_D292
4	ACCN5	CN_D318	CN_D292	CN_D252	CN_D225
4	ACAP1	CN_D318	CN_D225	CN_D224	CN_D292
4	ABL1	CN_D302.2	CN_D292	CN_D225	CN_D292
4	AADACL4	CN_D318	CN_D296	CN_D292	CN_D252

Supplementary Table 3: Significant genes of copy number duplications in at least 10% (4 samples) samples.

Frequency number	Gene symbol	Samples										
11	RIMS2	CN_328	CN_D318	CN_D310	CN_D296	CN_D288	CN_D254	CN_D253.2	CN_D228	CN_D224	CN_330	CN_328
11	PBX1	CN_D310	CN_D302.2	CN_D300	CN_D294	CN_D292	CN_D288	CN_D253.2	CN_D252	CN_D251	CN_D224	CN_320
11	DPYS	CN_328	CN_D318	CN_D310	CN_D296	CN_D288	CN_D254	CN_D253.2	CN_D251	CN_D228	CN_D224	CN_330
10	SAMD12	CN_328	CN_D318	CN_D310	CN_D296	CN_D288	CN_D254	CN_D251	CN_D224	CN_330	CN_328	
10	PTPRD	CN_D302.2	CN_D298	CN_D296	CN_D292	CN_D253.2	CN_D251	CN_D228	CN_D227	CN_D224	CN_324	
10	LRP12	CN_328	CN_D318	CN_D310	CN_D288	CN_D254	CN_D253.2	CN_D251	CN_D228	CN_D224	CN_330	
10	KCNK9	CN_D224	CN_328	CN_D318	CN_D310	CN_D296	CN_D288	CN_D254	CN_D253.2	CN_D251	CN_330	
10	KCNH1	CN_D316	CN_D310	CN_D300	CN_D298	CN_D294	CN_D292	CN_D288	CN_D253.2	CN_D252	CN_320	
9	ZFPM2	CN_D318	CN_D310	CN_D288	CN_D254	CN_D253.2	CN_D251	CN_D224	CN_330	CN_328		
9	ZFAT	CN_D318	CN_D310	CN_D296	CN_D288	CN_D254	CN_D253.2	CN_D224	CN_330	CN_328		
9	YWHAZ	CN_328	CN_D318	CN_D296	CN_D288	CN_D254	CN_D251	CN_D228	CN_D224	CN_330		
9	USH2A	CN_D310	CN_D300	CN_D298	CN_D294	CN_D292	CN_D288	CN_D253.2	CN_D252	CN_320		
9	TRAPPC9	CN_D224	CN_328	CN_D318	CN_D310	CN_D296	CN_D288	CN_D254	CN_D251	CN_330		
9	TNR	CN_D310	CN_D300	CN_D294	CN_D292	CN_D288	CN_D253.2	CN_D252	CN_D251	CN_D224		
9	TMEM64	CN_D224	CN_D318	CN_D310	CN_D296	CN_D288	CN_D254	CN_D251	CN_330	CN_328		
9	TM7SF4	CN_328	CN_D318	CN_D310	CN_D296	CN_D288	CN_D254	CN_D228	CN_D224	CN_330		
9	SLC7A13	CN_D318	CN_D296	CN_D288	CN_D254	CN_D253.2	CN_D251	CN_D224	CN_330	CN_328		
9	SH2D2A	CN_D310	CN_D302.2	CN_D296	CN_D294	CN_D292	CN_D288	CN_D252	CN_D251	CN_D224		
9	RSPO2	CN_328	CN_D318	CN_D310	CN_D288	CN_D254	CN_D251	CN_D224	CN_330	CN_328		
9	RASSF5	CN_D318	CN_D310	CN_D302.2	CN_D300	CN_D294	CN_D292	CN_D288	CN_D252	CN_320		
9	RALYL	CN_D224	CN_D318	CN_D310	CN_D296	CN_D288	CN_D254	CN_D224	CN_330	CN_328		
9	PVT1	CN_D224	CN_328	CN_D318	CN_D310	CN_D288	CN_D254	CN_D251	CN_D224	CN_330		
9	PROX1-AS1	CN_D310	CN_D300	CN_D298	CN_D296	CN_D294	CN_D292	CN_D288	CN_D252	CN_320		
9	PRCC	CN_D310	CN_D302.2	CN_D296	CN_D294	CN_D292	CN_D288	CN_D252	CN_D251	CN_D224		
9	NTRK1	CN_D310	CN_D302.2	CN_D296	CN_D294	CN_D292	CN_D288	CN_D252	CN_D251	CN_D224		
9	NECAB1	CN_D224	CN_D318	CN_D310	CN_D296	CN_D288	CN_D254	CN_D251	CN_330	CN_328		
9	LYST	CN_D302.2	CN_D300	CN_D298	CN_D296	CN_D294	CN_D292	CN_D288	CN_D252	CN_320		
9	LOC339529	CN_D310	CN_D302.2	CN_D300	CN_D296	CN_D294	CN_D292	CN_D288	CN_D252	CN_320		
9	LOC100616530	CN_D318	CN_D310	CN_D296	CN_D288	CN_D254	CN_D251	CN_D224	CN_330	CN_328		
9	LGALS8	CN_D310	CN_D300	CN_D298	CN_D296	CN_D294	CN_D292	CN_D288	CN_D252	CN_320		
9	KCNQ3	CN_D318	CN_D310	CN_D296	CN_D288	CN_D254	CN_D251	CN_D224	CN_330	CN_328		
9	KCNK2	CN_D310	CN_D302.2	CN_D300	CN_D294	CN_D292	CN_D288	CN_D253.2	CN_D252	CN_320		
9	INTS7	CN_D318	CN_D310	CN_D302.2	CN_D300	CN_D294	CN_D292	CN_D288	CN_D252	CN_320		
9	INSRR	CN_D310	CN_D302.2	CN_D296	CN_D294	CN_D292	CN_D288	CN_D252	CN_D251	CN_D224		
9	HNF4G	CN_D224	CN_D318	CN_D310	CN_D296	CN_D288	CN_D254	CN_D253.2	CN_330	CN_328		
9	HHAT	CN_D310	CN_D300	CN_D294	CN_D292	CN_D288	CN_D253.2	CN_D252	CN_328	CN_320		
9	GPATCH2	CN_D310	CN_D302.2	CN_D300	CN_D296	CN_D294	CN_D292	CN_D288	CN_D252	CN_320		
9	GALNT2	CN_D310	CN_D302.2	CN_D300	CN_D298	CN_D296	CN_D294	CN_D292	CN_D288	CN_D252		
9	FAM49B	CN_D318	CN_D310	CN_D296	CN_D288	CN_D254	CN_D251	CN_D224	CN_330	CN_328		
9	ESRRG	CN_D310	CN_D302.2	CN_D300	CN_D298	CN_D294	CN_D292	CN_D288	CN_D252	CN_320		
9	DTL	CN_D318	CN_D310	CN_D302.2	CN_D296	CN_D294	CN_D292	CN_D288	CN_D252	CN_320		

9	CSMD3	CN_D318	CN_D310	CN_D288	CN_D254	CN_D251	CN_D227	CN_D224	CN_330	CN_328
9	CEP350	CN_D310	CN_D302.2	CN_D300	CN_D294	CN_D292	CN_D253.2	CN_D252	CN_D224	CN_320
9	C8orf34	CN_D254	CN_D318	CN_D310	CN_D296	CN_D288	CN_D254	CN_D224	CN_330	CN_328
9	ACTN2	CN_D302.2	CN_D300	CN_D298	CN_D296	CN_D294	CN_D292	CN_D288	CN_D252	CN_320
8	ZHX2	CN_328	CN_D318	CN_D296	CN_D254	CN_D251	CN_D224	CN_330	CN_328	
8	YY1AP1	CN_D302.2	CN_D296	CN_D294	CN_D252	CN_D251	CN_D227	CN_D224	CN_320	
8	WWP1	CN_D318	CN_D310	CN_D296	CN_D288	CN_D254	CN_D224	CN_330	CN_328	
8	VPS13B	CN_D318	CN_D310	CN_D296	CN_D254	CN_D253.2	CN_D224	CN_330	CN_328	
8	TG	CN_328	CN_D318	CN_D310	CN_D296	CN_D288	CN_D254	CN_D224	CN_330	
8	TAP1	CN_D296	CN_D294	CN_D292	CN_D288	CN_D253.2	CN_D252	CN_D224	CN_328	
8	STK3	CN_D318	CN_D316	CN_D310	CN_D296	CN_D254	CN_D224	CN_330	CN_328	
8	ST3GAL1	CN_328	CN_D318	CN_D310	CN_D296	CN_D254	CN_D251	CN_D224	CN_330	
8	SPAG1	CN_D318	CN_D310	CN_D254	CN_D253.2	CN_D251	CN_D224	CN_330	CN_328	
8	SNTB1	CN_D318	CN_D310	CN_D296	CN_D288	CN_D254	CN_D224	CN_330	CN_328	
8	SMYD3	CN_D302.2	CN_D300	CN_D298	CN_D294	CN_D292	CN_D288	CN_D252	CN_330	
8	SLC26A7	CN_D224	CN_D318	CN_D310	CN_D296	CN_D254	CN_D251	CN_330	CN_328	
8	RYR2	CN_D310	CN_D300	CN_D298	CN_D294	CN_D292	CN_D288	CN_D253.2	CN_D252	
8	RNF19A	CN_D318	CN_D288	CN_D254	CN_D253.2	CN_D251	CN_D224	CN_330	CN_328	
8	RING1	CN_D316	CN_D296	CN_D292	CN_D288	CN_D253.2	CN_D252	CN_D224	CN_328	
8	RGS22	CN_D318	CN_D310	CN_D254	CN_D253.2	CN_D251	CN_D224	CN_330	CN_328	
8	RABGAP1L	CN_D310	CN_D302.2	CN_D294	CN_D292	CN_D288	CN_D252	CN_D224	CN_320	
8	PSMB9	CN_D296	CN_D294	CN_D292	CN_D288	CN_D253.2	CN_D252	CN_D224	CN_328	
8	PSKH2	CN_D318	CN_D296	CN_D288	CN_D254	CN_D251	CN_D224	CN_330	CN_328	
8	POLR2K	CN_D318	CN_D310	CN_D254	CN_D253.2	CN_D251	CN_D224	CN_330	CN_328	
8	PEAR1	CN_D310	CN_D302.2	CN_D294	CN_D292	CN_D288	CN_D252	CN_D251	CN_D224	
8	PABPC1	CN_328	CN_D318	CN_D310	CN_D296	CN_D288	CN_D254	CN_D224	CN_330	
8	NOS1AP	CN_D310	CN_D294	CN_D292	CN_D288	CN_D252	CN_D251	CN_D224	CN_320	
8	NKAIN3	CN_D224	CN_D318	CN_D310	CN_D296	CN_D288	CN_D254	CN_330	CN_328	
8	NCALD	CN_328	CN_D318	CN_D310	CN_D254	CN_D253.2	CN_D228	CN_D224	CN_330	
8	MIR765	CN_D310	CN_D302.2	CN_D294	CN_D292	CN_D288	CN_D252	CN_D251	CN_D224	
8	MIR4661	CN_D224	CN_D318	CN_D310	CN_D296	CN_D254	CN_D251	CN_330	CN_328	
8	MIR3122	CN_D318	CN_D310	CN_D302.2	CN_D294	CN_D292	CN_D288	CN_D252	CN_320	
8	MIR1208	CN_328	CN_D318	CN_D310	CN_D288	CN_D254	CN_D251	CN_D224	CN_330	
8	LRRC71	CN_D310	CN_D302.2	CN_D294	CN_D292	CN_D288	CN_D252	CN_D251	CN_D224	
8	LRRC69	CN_D224	CN_D318	CN_D310	CN_D296	CN_D254	CN_D251	CN_330	CN_328	
8	LOC286190	CN_328	CN_D318	CN_D310	CN_D296	CN_D288	CN_D254	CN_D224	CN_330	
8	LOC100294145	CN_D296	CN_D294	CN_D292	CN_D288	CN_D253.2	CN_D252	CN_D224	CN_328	
8	LOC100132891	CN_D224	CN_D318	CN_D310	CN_D296	CN_D288	CN_D254	CN_330	CN_328	
8	LINC00536	CN_D318	CN_D310	CN_D288	CN_D254	CN_D251	CN_D224	CN_330	CN_328	
8	LGALS8-AS1	CN_D310	CN_D300	CN_D296	CN_D294	CN_D292	CN_D288	CN_D252	CN_320	
8	LAMC1	CN_D302.2	CN_D296	CN_D294	CN_D292	CN_D288	CN_D253.2	CN_D252	CN_D224	
8	LACTB2	CN_328	CN_D318	CN_D310	CN_D296	CN_D288	CN_D254	CN_D224	CN_330	
8	KIF26B	CN_D310	CN_D302.2	CN_D300	CN_D296	CN_D294	CN_D292	CN_D252	CN_320	

8	IKBKE	CN_D318	CN_D310	CN_D302.2	CN_D294	CN_D292	CN_D288	CN_D252	CN_320
8	HMCN1	CN_D302.2	CN_D300	CN_D296	CN_D294	CN_D292	CN_D288	CN_D252	CN_D224
8	HLA-DRB1	ZS.DNA24	CN_D316	CN_D296	CN_D292	CN_D288	CN_D252	CN_D224	CN_328
8	HLA-DOA	CN_D316	CN_D296	CN_D292	CN_D288	CN_D253.2	CN_D252	CN_D224	CN_328
8	HLA-DMB	CN_D296	CN_D294	CN_D292	CN_D288	CN_D253.2	CN_D252	CN_D224	CN_328
8	HIST2H2BF	CN_D300	CN_D292	CN_D288	CN_D251	CN_D227	CN_D224	CN_328	CN_320
8	HHLA1	CN_D318	CN_D310	CN_D288	CN_D254	CN_D251	CN_D224	CN_330	CN_328
8	HEATR1	CN_D310	CN_D298	CN_D296	CN_D294	CN_D292	CN_D288	CN_D252	CN_320
8	HDGF	CN_D302.2	CN_D296	CN_D294	CN_D292	CN_D288	CN_D252	CN_D251	CN_D224
8	GSDMC	CN_D318	CN_D310	CN_D288	CN_D254	CN_D251	CN_D224	CN_330	CN_328
8	GEM	CN_D224	CN_D318	CN_D310	CN_D288	CN_D254	CN_D251	CN_330	CN_328
8	FER1L6	CN_D224	CN_328	CN_D318	CN_D310	CN_D288	CN_D254	CN_D251	CN_330
8	FER1L6-AS1	CN_D224	CN_328	CN_D318	CN_D310	CN_D288	CN_D254	CN_D251	CN_330
8	FCGR1A	CN_D300	CN_D292	CN_D288	CN_D251	CN_D227	CN_D224	CN_328	CN_320
8	FBXO43	CN_D318	CN_D310	CN_D254	CN_D253.2	CN_D251	CN_D224	CN_330	CN_328
8	FAM5C	CN_D310	CN_D302.2	CN_D294	CN_D292	CN_D288	CN_D252	CN_D224	CN_320
8	EXT1	CN_328	CN_D318	CN_D310	CN_D296	CN_D254	CN_D251	CN_D224	CN_330
8	EIF2D	CN_D318	CN_D310	CN_D302.2	CN_D300	CN_D294	CN_D292	CN_D252	CN_320
8	DEPTOR	CN_D318	CN_D310	CN_D288	CN_D254	CN_D251	CN_D224	CN_330	CN_328
8	DENND1B	ZS.DNA25	CN_D310	CN_D296	CN_D294	CN_D292	CN_D252	CN_D224	CN_320
8	CSMD1	CN_D302.2	CN_D298	CN_D294	CN_D292	CN_D253.2	CN_D251	CN_D228	CN_324
8	CR1L	CN_D310	CN_D302.2	CN_D300	CN_D294	CN_D292	CN_D288	CN_D252	CN_320
8	COX6C	CN_D318	CN_D310	CN_D296	CN_D254	CN_D253.2	CN_D224	CN_330	CN_328
8	COL22A1	CN_D224	CN_D318	CN_D310	CN_D288	CN_D254	CN_D251	CN_330	CN_328
8	COL14A1	CN_D318	CN_D310	CN_D296	CN_D288	CN_D254	CN_D224	CN_330	CN_328
8	CNIH3	CN_D310	CN_D302.2	CN_D300	CN_D296	CN_D294	CN_D292	CN_D252	CN_320
8	CNBD1	CN_D296	CN_D318	CN_D310	CN_D288	CN_D254	CN_D224	CN_330	CN_328
8	CLVS1	CN_D224	CN_D318	CN_D310	CN_D288	CN_D254	CN_D224	CN_330	CN_328
8	CDH13	CN_D302.2	CN_D300	CN_D298	CN_D296	CN_D252	CN_328	CN_324	CN_320
8	CDC73	CN_D310	CN_D302.2	CN_D296	CN_D294	CN_D292	CN_D288	CN_D252	CN_D224
8	CAMSAP2	CN_D310	CN_D302.2	CN_D294	CN_D292	CN_D253.2	CN_D252	CN_D224	CN_320
8	C8orf87	CN_D224	CN_D318	CN_D310	CN_D296	CN_D288	CN_D254	CN_330	CN_328
8	BRD2	CN_D296	CN_D294	CN_D292	CN_D288	CN_D253.2	CN_D252	CN_D224	CN_328
8	ATP6V0D2	CN_D318	CN_D296	CN_D288	CN_D254	CN_D251	CN_D224	CN_330	CN_328
8	ASAP1	CN_D318	CN_D296	CN_D288	CN_D254	CN_D251	CN_D224	CN_330	CN_328
8	ARHGEF11	CN_D310	CN_D302.2	CN_D294	CN_D292	CN_D288	CN_D252	CN_D251	CN_D224
8	AKT3	CN_D310	CN_D302.2	CN_D300	CN_D296	CN_D294	CN_D292	CN_D252	CN_320
8	ADCY8	CN_D318	CN_D310	CN_D288	CN_D254	CN_D251	CN_D224	CN_330	CN_328
7	ZFHX4	CN_D224	CN_D318	CN_D310	CN_D296	CN_D254	CN_330	CN_328	
7	XKR9	CN_328	CN_D310	CN_D296	CN_D288	CN_D254	CN_D224	CN_330	
7	XKR4	CN_D224	CN_328	CN_D318	CN_D310	CN_D288	CN_D253.2	CN_330	
7	WDR67	CN_328	CN_D318	CN_D296	CN_D288	CN_D254	CN_D224	CN_330	
7	VPS52	CN_D316	CN_D296	CN_D292	CN_D288	CN_D253.2	CN_D252	CN_328	

7	UHMK1	CN_D294	CN_D292	CN_D288	CN_D252	CN_D251	CN_D224	CN_320
7	UG0898H09	CN_D224	CN_D318	CN_D310	CN_D288	CN_D254	CN_330	CN_328
7	UCHL5	CN_D310	CN_D294	CN_D292	CN_D288	CN_D252	CN_D224	CN_320
7	UBXN2B	CN_D224	CN_328	CN_D318	CN_D310	CN_D288	CN_D254	CN_D253.2
7	UAP1	CN_D294	CN_D292	CN_D288	CN_D252	CN_D251	CN_D224	CN_320
7	TSNAX-DISC1	CN_D310	CN_D300	CN_D294	CN_D292	CN_D288	CN_D252	CN_320
7	TRPS1	CN_D318	CN_D310	CN_D296	CN_D254	CN_D224	CN_330	CN_328
7	TRAM1	CN_328	CN_D318	CN_D310	CN_D288	CN_D254	CN_D224	CN_330
7	TNFRSF11B	CN_D318	CN_D310	CN_D288	CN_D254	CN_D224	CN_330	CN_328
7	TMEM65	CN_D224	CN_328	CN_D318	CN_D310	CN_D288	CN_D254	CN_330
7	TMEM55A	CN_D224	CN_D318	CN_D310	CN_D296	CN_D254	CN_330	CN_328
7	TEDDM1	CN_D310	CN_D302.2	CN_D294	CN_D292	CN_D288	CN_D252	CN_D224
7	TBX19	CN_D310	CN_D302.2	CN_D294	CN_D292	CN_D288	CN_D252	CN_D224
7	TBCE	CN_D310	CN_D302.2	CN_D300	CN_D294	CN_D292	CN_D252	CN_320
7	TARS2	CN_D300	CN_D296	CN_D292	CN_D288	CN_D227	CN_D224	CN_320
7	TAF2	CN_D310	CN_D288	CN_D254	CN_D251	CN_D224	CN_330	CN_328
7	SUPT3H	CN_D296	CN_D292	CN_D288	CN_D253.2	CN_D252	CN_D224	CN_328
7	SRGAP2	CN_D318	CN_D310	CN_D294	CN_D292	CN_D288	CN_D252	CN_320
7	SPATA17	CN_D310	CN_D302.2	CN_D296	CN_D294	CN_D292	CN_D288	CN_D252
7	SNX31	CN_328	CN_D318	CN_D310	CN_D296	CN_D254	CN_D224	CN_330
7	SMYD2	CN_D310	CN_D300	CN_D296	CN_D294	CN_D292	CN_D288	CN_D252
7	SLCO5A1	CN_D254	CN_328	CN_D318	CN_D310	CN_D288	CN_D224	CN_330
7	SLC39A7	CN_D296	CN_D292	CN_D288	CN_D253.2	CN_D252	CN_D224	CN_328
7	SLC25A32	CN_D318	CN_D288	CN_D254	CN_D228	CN_D224	CN_330	CN_328
7	SLC19A2	CN_D310	CN_D294	CN_D292	CN_D288	CN_D252	CN_D224	CN_320
7	SLA	CN_328	CN_D318	CN_D310	CN_D296	CN_D254	CN_D224	CN_330
7	SH2D1B	CN_D310	CN_D294	CN_D292	CN_D288	CN_D252	CN_D251	CN_D224
7	SELP	CN_D310	CN_D294	CN_D292	CN_D288	CN_D253.2	CN_D252	CN_D224
7	SDCBP	CN_328	CN_D318	CN_D310	CN_D288	CN_D254	CN_D253.2	CN_D224
7	SAMD12-AS1	CN_D318	CN_D288	CN_D254	CN_D251	CN_D224	CN_330	CN_328
7	RUNX1T1	CN_D224	CN_D318	CN_D310	CN_D288	CN_D254	CN_330	CN_328
7	RRNAD1	CN_D296	CN_D294	CN_D292	CN_D288	CN_D252	CN_D251	CN_D224
7	RRM2B	CN_328	CN_D318	CN_D254	CN_D253.2	CN_D251	CN_D224	CN_330
7	RPS6KC1	CN_D310	CN_D300	CN_D298	CN_D294	CN_D292	CN_D288	CN_D252
7	RPRD2	CN_D300	CN_D296	CN_D292	CN_D288	CN_D227	CN_D224	CN_320
7	RHOU	CN_D310	CN_D298	CN_D296	CN_D292	CN_D288	CN_D252	CN_320
7	RGSL1	CN_D310	CN_D294	CN_D292	CN_D288	CN_D253.2	CN_D252	CN_D224
7	RGS8	CN_D310	CN_D300	CN_D298	CN_D292	CN_D288	CN_D252	CN_D224
7	RGS21	CN_D302.2	CN_D296	CN_D294	CN_D292	CN_D288	CN_D253.2	CN_D252
7	RFWD2	CN_D310	CN_D294	CN_D292	CN_D288	CN_D252	CN_D251	CN_D224
7	RAD54B	CN_D224	CN_D318	CN_D288	CN_D254	CN_D224	CN_330	CN_328
7	QSOX1	CN_D310	CN_D300	CN_D294	CN_D292	CN_D252	CN_D224	CN_320
7	PSMB8	CN_D296	CN_D292	CN_D288	CN_D253.2	CN_D252	CN_D224	CN_328

7	PRRX1	CN_D310	CN_D302.2	CN_D294	CN_D292	CN_D288	CN_D252	CN_320
7	PLA2G4A	CN_D310	CN_D294	CN_D292	CN_D288	CN_D252	CN_D224	CN_320
7	PI15	CN_328	CN_D318	CN_D310	CN_D288	CN_D254	CN_D224	CN_330
7	PARK2	CN_D298	CN_D296	CN_D288	CN_D254	CN_D253.2	CN_D251	CN_D224
7	PAPPA2	CN_D310	CN_D294	CN_D292	CN_D288	CN_D252	CN_D224	CN_320
7	PAG1	CN_328	CN_D318	CN_D310	CN_D296	CN_D254	CN_D224	CN_330
7	OXR1	CN_D318	CN_D310	CN_D288	CN_D254	CN_D224	CN_330	CN_328
7	OTUD6B	CN_D224	CN_D318	CN_D310	CN_D296	CN_D254	CN_330	CN_328
7	OSGIN2	CN_D224	CN_D318	CN_D296	CN_D288	CN_D254	CN_330	CN_328
7	OC90	CN_D318	CN_D310	CN_D288	CN_D254	CN_D224	CN_330	CN_328
7	NSMCE2	CN_D224	CN_328	CN_D318	CN_D310	CN_D288	CN_D254	CN_330
7	NBN	CN_D224	CN_D318	CN_D296	CN_D288	CN_D254	CN_330	CN_328
7	MTSS1	CN_D224	CN_328	CN_D318	CN_D310	CN_D288	CN_D254	CN_330
7	MTFR1	CN_D224	CN_328	CN_D318	CN_D310	CN_D288	CN_D254	CN_330
7	MTDH	CN_D318	CN_D288	CN_D254	CN_D251	CN_D224	CN_330	CN_328
7	MTBP	CN_D318	CN_D296	CN_D288	CN_D254	CN_D224	CN_330	CN_328
7	MRPL24	CN_D296	CN_D294	CN_D292	CN_D288	CN_D252	CN_D251	CN_D224
7	MRPL13	CN_D318	CN_D296	CN_D288	CN_D254	CN_D224	CN_330	CN_328
7	MMP16	CN_D224	CN_D318	CN_D296	CN_D288	CN_D254	CN_330	CN_328
7	MIR875	CN_D318	CN_D310	CN_D296	CN_D254	CN_D224	CN_330	CN_328
7	MIR599	CN_D318	CN_D310	CN_D296	CN_D254	CN_D224	CN_330	CN_328
7	MIR4470	CN_D224	CN_D318	CN_D310	CN_D288	CN_D254	CN_330	CN_328
7	MIR30D	CN_D318	CN_D296	CN_D288	CN_D254	CN_D224	CN_330	CN_328
7	MIR30B	CN_D318	CN_D296	CN_D288	CN_D254	CN_D224	CN_330	CN_328
7	MIR29C	CN_D310	CN_D302.2	CN_D294	CN_D292	CN_D288	CN_D252	CN_320
7	MIR29B2	CN_D310	CN_D302.2	CN_D294	CN_D292	CN_D288	CN_D252	CN_320
7	MIR219-1	CN_D296	CN_D292	CN_D288	CN_D253.2	CN_D252	CN_D224	CN_328
7	MIR2052	CN_328	CN_D318	CN_D310	CN_D288	CN_D254	CN_D224	CN_330
7	MIR124-2	CN_D224	CN_D318	CN_D310	CN_D296	CN_D288	CN_D254	CN_330
7	MIR1207	CN_328	CN_D318	CN_D288	CN_D254	CN_D251	CN_D224	CN_330
7	MIR1206	CN_328	CN_D318	CN_D288	CN_D254	CN_D251	CN_D224	CN_330
7	LY6H	CN_328	CN_D318	CN_D310	CN_D288	CN_D254	CN_D224	CN_330
7	LRRC6	CN_D318	CN_D310	CN_D254	CN_D253.2	CN_D224	CN_330	CN_328
7	LOC440704	CN_D310	CN_D294	CN_D292	CN_D288	CN_D252	CN_D224	CN_320
7	LOC401463	CN_D224	CN_328	CN_D318	CN_D310	CN_D288	CN_D254	CN_330
7	LOC286189	CN_D318	CN_D310	CN_D296	CN_D254	CN_D224	CN_330	CN_328
7	LOC286186	CN_D224	CN_328	CN_D318	CN_D310	CN_D288	CN_D254	CN_330
7	LOC284648	CN_D310	CN_D300	CN_D294	CN_D292	CN_D288	CN_D252	CN_D224
7	LOC100527964	CN_D310	CN_D300	CN_D294	CN_D292	CN_D252	CN_D224	CN_320
7	LOC100507463	CN_D296	CN_D292	CN_D288	CN_D253.2	CN_D252	CN_D224	CN_328
7	LOC100507117	CN_D318	CN_D296	CN_D288	CN_D254	CN_D224	CN_330	CN_328
7	LOC100506810	CN_D310	CN_D300	CN_D294	CN_D292	CN_D288	CN_D252	CN_320
7	LOC100505718	CN_D254	CN_D318	CN_D310	CN_D288	CN_D224	CN_330	CN_328

7	LOC100192378	CN_D224	CN_D318	CN_D310	CN_D296	CN_D254	CN_330	CN_328
7	LOC100130298	CN_D318	CN_D310	CN_D296	CN_D254	CN_D224	CN_330	CN_328
7	LOC100130155	CN_D224	CN_D318	CN_D310	CN_D296	CN_D288	CN_D254	CN_330
7	LOC100127983	CN_D224	CN_D318	CN_D310	CN_D296	CN_D254	CN_330	CN_328
7	LINC00535	CN_D224	CN_D318	CN_D310	CN_D254	CN_D251	CN_330	CN_328
7	LINC00272	CN_D310	CN_D302.2	CN_D294	CN_D292	CN_D288	CN_D252	CN_D224
7	LINC00251	CN_D224	CN_328	CN_D318	CN_D310	CN_D288	CN_D254	CN_330
7	LHX4	CN_D310	CN_D300	CN_D294	CN_D292	CN_D252	CN_D224	CN_320
7	LAMC2	CN_D310	CN_D302.2	CN_D294	CN_D292	CN_D288	CN_D252	CN_D224
7	KLF10	CN_328	CN_D318	CN_D296	CN_D254	CN_D228	CN_D224	CN_330
7	KIAA0196	CN_D224	CN_328	CN_D318	CN_D310	CN_D288	CN_D254	CN_330
7	KCNV1	CN_D318	CN_D310	CN_D296	CN_D254	CN_D224	CN_330	CN_328
7	KCNT2	CN_D310	CN_D302.2	CN_D300	CN_D292	CN_D288	CN_D252	CN_D224
7	HSD17B8	CN_D296	CN_D292	CN_D288	CN_D253.2	CN_D252	CN_D224	CN_328
7	HLA-DQB2	CN_D296	CN_D294	CN_D292	CN_D288	CN_D253.2	CN_D252	CN_328
7	HLA-DQA2	CN_D296	CN_D294	CN_D292	CN_D288	CN_D253.2	CN_D252	CN_328
7	HLA-DPB1	CN_D296	CN_D292	CN_D288	CN_D253.2	CN_D252	CN_D224	CN_328
7	HLA-DPA1	CN_D296	CN_D292	CN_D288	CN_D253.2	CN_D252	CN_D224	CN_328
7	HLA-DMA	CN_D296	CN_D292	CN_D288	CN_D253.2	CN_D252	CN_D224	CN_328
7	HIST2H4B	CN_D300	CN_D292	CN_D288	CN_D251	CN_D227	CN_D224	CN_328
7	HIST2H4A	CN_D300	CN_D292	CN_D288	CN_D251	CN_D227	CN_D224	CN_328
7	HIST2H3D	CN_D300	CN_D292	CN_D288	CN_D251	CN_D227	CN_D224	CN_328
7	HIST2H3C	CN_D300	CN_D292	CN_D288	CN_D251	CN_D227	CN_D224	CN_328
7	HIST2H3A	CN_D300	CN_D292	CN_D288	CN_D251	CN_D227	CN_D224	CN_328
7	HIST2H2AA4	CN_D300	CN_D292	CN_D288	CN_D251	CN_D227	CN_D224	CN_328
7	HIST2H2AA3	CN_D300	CN_D292	CN_D288	CN_D251	CN_D227	CN_D224	CN_328
7	GPR161	CN_D310	CN_D296	CN_D294	CN_D292	CN_D288	CN_D252	CN_D224
7	GLUL	CN_D310	CN_D302.2	CN_D294	CN_D292	CN_D288	CN_D252	CN_D224
7	GGH	CN_D224	CN_D318	CN_D310	CN_D288	CN_D254	CN_330	CN_328
7	GDF6	CN_D318	CN_D310	CN_D254	CN_D251	CN_D224	CN_330	CN_328
7	FZD6	CN_D318	CN_D288	CN_D254	CN_D228	CN_D224	CN_330	CN_328
7	FLJ46284	CN_D224	CN_D318	CN_D310	CN_D296	CN_D254	CN_330	CN_328
7	FLJ42969	CN_328	CN_D318	CN_D288	CN_D254	CN_D251	CN_D224	CN_330
7	FLJ39080	CN_328	CN_D318	CN_D310	CN_D288	CN_D254	CN_D224	CN_330
7	FLJ23867	CN_D310	CN_D300	CN_D294	CN_D292	CN_D252	CN_D224	CN_320
7	FAM82B	CN_D318	CN_D310	CN_D288	CN_D254	CN_D224	CN_330	CN_328
7	FAM163A	CN_D310	CN_D300	CN_D296	CN_D294	CN_D292	CN_D252	CN_D224
7	F5	CN_D310	CN_D294	CN_D292	CN_D288	CN_D252	CN_D224	CN_320
7	F13A1	CN_D296	CN_D294	CN_D292	CN_D288	CN_D253.2	CN_D252	CN_328
7	EYA1	CN_D318	CN_D310	CN_D288	CN_D254	CN_D224	CN_330	CN_328
7	ENPP2	CN_D318	CN_D288	CN_D254	CN_D251	CN_D224	CN_330	CN_328
7	EFR3A	CN_D318	CN_D310	CN_D288	CN_D254	CN_D224	CN_330	CN_328
7	ECM1	CN_D300	CN_D296	CN_D292	CN_D288	CN_D227	CN_D224	CN_320

7	DYRK3	CN_D318	CN_D310	CN_D302.2	CN_D300	CN_D294	CN_D292	CN_D252
7	DSCC1	CN_D310	CN_D288	CN_D254	CN_D251	CN_D224	CN_330	CN_328
7	DNM3	CN_D300	CN_D296	CN_D294	CN_D292	CN_D252	CN_D224	CN_320
7	DISC1	CN_D310	CN_D300	CN_D294	CN_D292	CN_D288	CN_D252	CN_320
7	DECR1	CN_D224	CN_D318	CN_D296	CN_D288	CN_D254	CN_330	CN_328
7	DCAF13	CN_D318	CN_D288	CN_D254	CN_D228	CN_D224	CN_330	CN_328
7	CYP7B1	CN_D224	CN_328	CN_D318	CN_D310	CN_D288	CN_D254	CN_330
7	CYP7A1	CN_D224	CN_328	CN_D318	CN_D310	CN_D288	CN_D254	CN_D253.2
7	CYP11B2	CN_328	CN_D318	CN_D288	CN_D254	CN_D251	CN_D224	CN_330
7	CYP11B1	CN_328	CN_D318	CN_D288	CN_D254	CN_D251	CN_D224	CN_330
7	CTHRC1	CN_D318	CN_D288	CN_D254	CN_D228	CN_D224	CN_330	CN_328
7	CRHR1	ZS.DNA26	ZS.DNA11	CN_D306	CN_D296	CN_D224	CN_328	CN_324
7	CRB1	CN_D310	CN_D294	CN_D292	CN_D288	CN_D252	CN_D224	CN_320
7	CPQ	CN_D318	CN_D310	CN_D296	CN_D254	CN_D224	CN_330	CN_328
7	CPNE3	CN_D318	CN_D310	CN_D288	CN_D254	CN_D224	CN_330	CN_328
7	COLEC10	CN_D318	CN_D310	CN_D288	CN_D254	CN_D224	CN_330	CN_328
7	CNGB3	CN_D296	CN_D318	CN_D288	CN_D254	CN_D224	CN_330	CN_328
7	CHRM3	CN_D310	CN_D300	CN_D296	CN_D294	CN_D292	CN_D288	CN_D252
7	CFHR5	CN_D310	CN_D294	CN_D292	CN_D288	CN_D252	CN_D224	CN_320
7	CFHR2	CN_D310	CN_D294	CN_D292	CN_D288	CN_D252	CN_D224	CN_320
7	CENPF	CN_D310	CN_D302.2	CN_D300	CN_D294	CN_D292	CN_D288	CN_D252
7	CDKAL1	CN_D296	CN_D294	CN_D292	CN_D288	CN_D253.2	CN_D252	CN_D224
7	CDH6	CN_D224	CN_D296	CN_D288	CN_D253.2	CN_D252	CN_D225	CN_328
7	CD84	CN_D302.2	CN_D294	CN_D292	CN_D288	CN_D252	CN_D251	CN_D224
7	CD46	CN_D310	CN_D302.2	CN_D294	CN_D292	CN_D288	CN_D252	CN_320
7	CA3	CN_D224	CN_D318	CN_D296	CN_D254	CN_D251	CN_330	CN_328
7	CA2	CN_D224	CN_D318	CN_D296	CN_D254	CN_D251	CN_330	CN_328
7	C8orf37	CN_D318	CN_D310	CN_D288	CN_D254	CN_D224	CN_330	CN_328
7	C6orf10	CN_D296	CN_D292	CN_D288	CN_D253.2	CN_D252	CN_D224	CN_328
7	C1orf226	CN_D310	CN_D294	CN_D292	CN_D288	CN_D252	CN_D251	CN_D224
7	C1orf21	CN_D296	CN_D294	CN_D292	CN_D288	CN_D252	CN_D224	CN_320
7	BHLHE22	CN_D224	CN_328	CN_D318	CN_D310	CN_D288	CN_D254	CN_330
7	BAALC	CN_328	CN_D318	CN_D296	CN_D254	CN_D228	CN_D224	CN_330
7	ASTN1	CN_D296	CN_D294	CN_D292	CN_D288	CN_D252	CN_D224	CN_320
7	ASPH	CN_D224	CN_D318	CN_D310	CN_D288	CN_D254	CN_330	CN_328
7	ARMC1	CN_D224	CN_328	CN_D318	CN_D310	CN_D288	CN_D254	CN_330
7	ARL17A	CN_D316	CN_D306	CN_D296	CN_D251	CN_328	CN_324	CN_320
7	ARID4B	CN_D310	CN_D302.2	CN_D294	CN_D292	CN_D288	CN_D252	CN_320
7	ANGPT1	CN_D318	CN_D310	CN_D296	CN_D254	CN_D224	CN_330	CN_328
7	AKAP6	CN_D300	CN_D298	CN_D294	CN_D254	CN_D253.2	CN_D251	CN_320
7	ADAMTS4	CN_D310	CN_D300	CN_D294	CN_D292	CN_D252	CN_D251	CN_D224
7	ADAMTS2	ZS.DNA12	CN_D316	CN_D288	CN_D254	CN_D253.2	CN_D224	CN_328
7	ACBD6	CN_D310	CN_D300	CN_D294	CN_D292	CN_D252	CN_D224	CN_320

6	ZNRD1	CN_D296	CN_D292	CN_D288	CN_D253.2	CN_D252	CN_328
6	ZNRD1-AS1	CN_D296	CN_D292	CN_D288	CN_D253.2	CN_D252	CN_328
6	ZNF7	CN_328	CN_D288	CN_D254	CN_D251	CN_D224	CN_330
6	ZNF706	CN_328	CN_D318	CN_D254	CN_D251	CN_D224	CN_330
6	ZNF572	CN_D224	CN_328	CN_D318	CN_D288	CN_D254	CN_330
6	ZNF517	CN_328	CN_D288	CN_D254	CN_D251	CN_D224	CN_330
6	ZNF384	CN_D300	CN_D298	CN_D254	CN_D252	CN_328	CN_324
6	ZNF34	CN_328	CN_D288	CN_D254	CN_D251	CN_D224	CN_330
6	ZNF251	CN_328	CN_D288	CN_D254	CN_D251	CN_D224	CN_330
6	ZNF250	CN_328	CN_D288	CN_D254	CN_D251	CN_D224	CN_330
6	ZFP41	CN_328	CN_D318	CN_D310	CN_D254	CN_D224	CN_330
6	ZFAT-AS1	CN_D318	CN_D288	CN_D254	CN_D224	CN_330	CN_328
6	WWOX	CN_D300	CN_D296	CN_D294	CN_D253.2	CN_D251	CN_320
6	WRNIP1	CN_D296	CN_D292	CN_D288	CN_D253.2	CN_D252	CN_328
6	WISP1	CN_328	CN_D318	CN_D296	CN_D254	CN_D224	CN_330
6	VPS28	CN_328	CN_D288	CN_D254	CN_D251	CN_D224	CN_330
6	VCPIP1	CN_328	CN_D318	CN_D310	CN_D254	CN_D224	CN_330
6	VASH2	CN_D300	CN_D298	CN_D294	CN_D292	CN_D288	CN_D252
6	UTP23	CN_D318	CN_D288	CN_D254	CN_D224	CN_330	CN_328
6	USP49	CN_D296	CN_D292	CN_D288	CN_D253.2	CN_D252	CN_328
6	UQCRB	CN_D318	CN_D254	CN_D251	CN_D224	CN_330	CN_328
6	UCK2	CN_D300	CN_D296	CN_D294	CN_D292	CN_D252	CN_320
6	UBR5	CN_328	CN_D318	CN_D254	CN_D251	CN_D224	CN_330
6	UBQLN4	CN_D294	CN_D292	CN_D252	CN_D251	CN_D227	CN_D224
6	TTPA	CN_D224	CN_D310	CN_D288	CN_D254	CN_330	CN_328
6	TTC35	CN_328	CN_D310	CN_D288	CN_D254	CN_D224	CN_330
6	TTC24	CN_D294	CN_D292	CN_D288	CN_D253.2	CN_D252	CN_D224
6	TSNARE1	CN_328	CN_D318	CN_D296	CN_D254	CN_D224	CN_330
6	TRPA1	CN_D224	CN_D318	CN_D310	CN_D254	CN_330	CN_328
6	TROVE2	CN_D310	CN_D294	CN_D292	CN_D288	CN_D252	CN_D224
6	TRIM40	CN_D296	CN_D292	CN_D288	CN_D253.2	CN_D252	CN_328
6	TRIM31	CN_D296	CN_D292	CN_D288	CN_D253.2	CN_D252	CN_328
6	TRIM26	CN_D296	CN_D292	CN_D288	CN_D253.2	CN_D252	CN_328
6	TRIM15	CN_D296	CN_D292	CN_D288	CN_D253.2	CN_D252	CN_328
6	TRIM10	CN_D296	CN_D292	CN_D288	CN_D253.2	CN_D252	CN_328
6	TRHR	CN_D288	CN_D254	CN_D253.2	CN_D224	CN_330	CN_328
6	TRAM2	CN_D296	CN_D292	CN_D288	CN_D253.2	CN_D252	CN_328
6	TPD52	CN_328	CN_D318	CN_D288	CN_D254	CN_D224	CN_330
6	TP53INP1	CN_D318	CN_D288	CN_D254	CN_D224	CN_330	CN_328
6	TOX	CN_328	CN_D318	CN_D310	CN_D296	CN_D254	CN_D224
6	TONSL	CN_328	CN_D288	CN_D254	CN_D251	CN_D224	CN_330
6	TOMM6	CN_D296	CN_D292	CN_D288	CN_D253.2	CN_D252	CN_328
6	TNN	CN_D310	CN_D294	CN_D292	CN_D288	CN_D252	CN_D224

6	TNF	CN_D296	CN_D292	CN_D288	CN_D253.2	CN_D252	CN_328
6	TMEM67	CN_D224	CN_D254	CN_D253.2	CN_D251	CN_330	CN_328
6	TIPRL	CN_D310	CN_D302.2	CN_D292	CN_D288	CN_D252	CN_D224
6	TGFB2	CN_D298	CN_D296	CN_D294	CN_D292	CN_D288	CN_D252
6	TATDN3	CN_D298	CN_D294	CN_D292	CN_D288	CN_D252	CN_320
6	TAP2	CN_D296	CN_D292	CN_D288	CN_D253.2	CN_D252	CN_328
6	SULF1	CN_D254	CN_328	CN_D318	CN_D288	CN_D224	CN_330
6	STMN2	CN_328	CN_D318	CN_D310	CN_D254	CN_D224	CN_330
6	STK38	CN_D296	CN_D292	CN_D288	CN_D253.2	CN_D252	CN_328
6	STAU2	CN_328	CN_D318	CN_D296	CN_D254	CN_D224	CN_330
6	SSR2	CN_D294	CN_D292	CN_D252	CN_D251	CN_D227	CN_D224
6	SRSF3	CN_D296	CN_D292	CN_D288	CN_D253.2	CN_D252	CN_328
6	SQLE	CN_D224	CN_328	CN_D318	CN_D288	CN_D254	CN_330
6	SPATS1	CN_D296	CN_D292	CN_D288	CN_D253.2	CN_D252	CN_328
6	SNTB2	CN_D302.2	CN_D300	CN_D296	CN_328	CN_324	CN_320
6	SNORD84	CN_D296	CN_D292	CN_D288	CN_D253.2	CN_D252	CN_328
6	SNORD117	CN_D296	CN_D292	CN_D288	CN_D253.2	CN_D252	CN_328
6	SLC45A4	CN_328	CN_D310	CN_D254	CN_D253.2	CN_D224	CN_330
6	SLC39A4	CN_328	CN_D310	CN_D288	CN_D254	CN_D224	CN_330
6	SLC30A8	CN_D318	CN_D288	CN_D254	CN_D224	CN_330	CN_328
6	SLC26A9	CN_D310	CN_D294	CN_D292	CN_D288	CN_D252	CN_328
6	SLC10A5	CN_328	CN_D318	CN_D310	CN_D254	CN_D224	CN_330
6	SLAMF6	CN_D294	CN_D292	CN_D288	CN_D252	CN_D251	CN_D224
6	SHCBP1L	CN_D294	CN_D292	CN_D288	CN_D253.2	CN_D252	CN_D224
6	SFTA2	CN_D292	CN_D288	CN_D253.2	CN_D252	CN_D224	CN_328
6	SFT2D2	CN_D310	CN_D302.2	CN_D292	CN_D288	CN_D252	CN_D224
6	SERPINB1	CN_D296	CN_D292	CN_D288	CN_D253.2	CN_D252	CN_328
6	SEMA4A	CN_D294	CN_D292	CN_D252	CN_D251	CN_D227	CN_D224
6	SDHC	CN_D310	CN_D292	CN_D288	CN_D253.2	CN_D252	CN_D224
6	SDCCAG8	CN_D300	CN_D296	CN_D294	CN_D253.2	CN_D252	CN_330
6	SDC2	CN_D318	CN_D310	CN_D254	CN_D224	CN_330	CN_328
6	SCARNA3	CN_D310	CN_D294	CN_D288	CN_D252	CN_D251	CN_D224
6	RXRB	CN_D292	CN_D288	CN_D253.2	CN_D252	CN_D224	CN_328
6	RUNX2	CN_D296	CN_D292	CN_D288	CN_D253.2	CN_D252	CN_328
6	RREB1	CN_D292	CN_D288	CN_D253.2	CN_D252	CN_D224	CN_328
6	RPL8	CN_328	CN_D288	CN_D254	CN_D251	CN_D224	CN_330
6	RPL7	CN_328	CN_D318	CN_D288	CN_D254	CN_D224	CN_330
6	RNF5P1	CN_D318	CN_D296	CN_D292	CN_D288	CN_D252	CN_328
6	RNF39	CN_D296	CN_D292	CN_D288	CN_D253.2	CN_D252	CN_328
6	RNF144B	CN_D316	CN_D296	CN_D292	CN_D288	CN_D252	CN_328
6	RNASEL	CN_D310	CN_D294	CN_D292	CN_D288	CN_D252	CN_D224
6	RIPK2	CN_D224	CN_D318	CN_D288	CN_D254	CN_330	CN_328
6	RIOK1	CN_D296	CN_D292	CN_D288	CN_D253.2	CN_D252	CN_328

6	RGS7	CN_D310	CN_D294	CN_D292	CN_D288	CN_D252	CN_320
6	RGS2	CN_D300	CN_D294	CN_D292	CN_D288	CN_D252	CN_D224
6	RGS18	CN_D300	CN_D294	CN_D292	CN_D288	CN_D252	CN_D224
6	RGS16	CN_D310	CN_D294	CN_D292	CN_D288	CN_D252	CN_D224
6	RECQL4	CN_328	CN_D288	CN_D254	CN_D251	CN_D224	CN_330
6	RDH10	CN_328	CN_D318	CN_D288	CN_D254	CN_D224	CN_330
6	RBM24	CN_D296	CN_D292	CN_D288	CN_D253.2	CN_D252	CN_328
6	RBM12B	CN_D224	CN_D254	CN_D253.2	CN_D251	CN_330	CN_328
6	RAD21	CN_D318	CN_D288	CN_D254	CN_D224	CN_330	CN_328
6	RAD21-AS1	CN_D318	CN_D288	CN_D254	CN_D224	CN_330	CN_328
6	RAB2A	CN_D318	CN_D310	CN_D254	CN_D224	CN_330	CN_328
6	RAB25	CN_D294	CN_D292	CN_D252	CN_D251	CN_D227	CN_D224
6	PYCR2	CN_D302.2	CN_D300	CN_D292	CN_D288	CN_D253.2	CN_D252
6	PTK2	CN_D224	CN_328	CN_D254	CN_D253.2	CN_D251	CN_330
6	PTDSS1	CN_D318	CN_D254	CN_D251	CN_D224	CN_330	CN_328
6	PRICKLE4	CN_D296	CN_D292	CN_D288	CN_D253.2	CN_D252	CN_328
6	PRDM14	CN_D254	CN_328	CN_D318	CN_D288	CN_D224	CN_330
6	PPP1R16A	CN_328	CN_D288	CN_D254	CN_D251	CN_D224	CN_330
6	PPP1R11	CN_D296	CN_D292	CN_D288	CN_D253.2	CN_D252	CN_328
6	PPOX	CN_D310	CN_D300	CN_D294	CN_D292	CN_D252	CN_D251
6	PLXNA2	CN_D302.2	CN_D300	CN_D294	CN_D292	CN_D288	CN_D252
6	PLD5	CN_D316	CN_D310	CN_D294	CN_D288	CN_D252	CN_320
6	PGBD5	CN_D310	CN_D302.2	CN_D296	CN_D294	CN_D292	CN_D252
6	PEX2	CN_D224	CN_D318	CN_D310	CN_D254	CN_330	CN_328
6	PDP1	CN_D224	CN_D316	CN_D254	CN_D253.2	CN_330	CN_328
6	PDE7A	CN_D224	CN_328	CN_D318	CN_D288	CN_D254	CN_330
6	PCNXL2	CN_D302.2	CN_D296	CN_D292	CN_D252	CN_D251	CN_320
6	PCAT1	CN_D224	CN_D318	CN_D254	CN_D253.2	CN_330	CN_328
6	PACRG	CN_D298	CN_D296	CN_D292	CN_D288	CN_D253.2	CN_D224
6	OSR2	CN_D318	CN_D310	CN_D254	CN_D224	CN_330	CN_328
6	OLFML2B	CN_D310	CN_D292	CN_D288	CN_D252	CN_D251	CN_D224
6	ODF1	CN_328	CN_D318	CN_D254	CN_D228	CN_D224	CN_330
6	OCLM	CN_D300	CN_D294	CN_D292	CN_D288	CN_D252	CN_D224
6	NUDCD1	CN_D310	CN_D288	CN_D254	CN_D224	CN_330	CN_328
6	NSMAF	CN_328	CN_D318	CN_D310	CN_D288	CN_D254	CN_D224
6	NSL1	CN_D298	CN_D294	CN_D292	CN_D288	CN_D252	CN_320
6	NPR3	CN_D224	CN_D298	CN_D296	CN_D288	CN_D252	CN_D225
6	NOTCH4	CN_D296	CN_D292	CN_D288	CN_D252	CN_D224	CN_328
6	NMNAT2	CN_D302.2	CN_D300	CN_D292	CN_D288	CN_D252	CN_D224
6	NME7	CN_D310	CN_D300	CN_D294	CN_D252	CN_D224	CN_320
6	NIT1	CN_D310	CN_D302.2	CN_D300	CN_D292	CN_D252	CN_D251
6	NIPAL2	CN_D318	CN_D310	CN_D254	CN_D224	CN_330	CN_328
6	NFKBIL1	CN_D296	CN_D292	CN_D288	CN_D253.2	CN_D252	CN_328

6	NDUFS2	CN_D310	CN_D300	CN_D294	CN_D292	CN_D252	CN_D224
6	NDRG1	CN_328	CN_D318	CN_D310	CN_D254	CN_D224	CN_330
6	NCR3	CN_D296	CN_D292	CN_D288	CN_D253.2	CN_D252	CN_328
6	NCOA2	CN_D254	CN_328	CN_D318	CN_D288	CN_D224	CN_330
6	NBPF10	CN_D310	CN_D296	CN_D288	CN_D251	CN_D227	CN_D224
6	NAV1	CN_D300	CN_D296	CN_D294	CN_D292	CN_D252	CN_D224
6	NACAP1	CN_328	CN_D318	CN_D254	CN_D251	CN_D224	CN_330
6	MYC	CN_D224	CN_D318	CN_D288	CN_D254	CN_330	CN_328
6	MYBL1	CN_328	CN_D318	CN_D310	CN_D254	CN_D224	CN_330
6	MUC22	CN_D292	CN_D288	CN_D253.2	CN_D252	CN_D224	CN_328
6	MUC21	CN_D292	CN_D288	CN_D253.2	CN_D252	CN_D224	CN_328
6	MTR	CN_D298	CN_D294	CN_D292	CN_D288	CN_D252	CN_320
6	MTERFD1	CN_D318	CN_D254	CN_D251	CN_D224	CN_330	CN_328
6	MSH5-SAPCD1	CN_D296	CN_D292	CN_D288	CN_D253.2	CN_D252	CN_328
6	MSH5	CN_D296	CN_D292	CN_D288	CN_D253.2	CN_D252	CN_328
6	MSC	CN_D224	CN_D318	CN_D310	CN_D288	CN_D254	CN_330
6	MRPS28	CN_328	CN_D318	CN_D310	CN_D254	CN_D224	CN_330
6	MRPS14	CN_D310	CN_D294	CN_D292	CN_D288	CN_D252	CN_D224
6	MPZ	CN_D310	CN_D292	CN_D288	CN_D253.2	CN_D252	CN_D224
6	MOCS1	CN_D292	CN_D288	CN_D253.2	CN_D252	CN_D224	CN_328
6	MIR9-1	CN_D292	CN_D288	CN_D253.2	CN_D252	CN_D251	CN_D224
6	MIR586	CN_D296	CN_D292	CN_D288	CN_D253.2	CN_D252	CN_328
6	MIR557	CN_D310	CN_D294	CN_D292	CN_D288	CN_D252	CN_D224
6	MIR548F1	CN_D300	CN_D294	CN_D292	CN_D288	CN_D252	CN_D224
6	MIR4753	CN_D310	CN_D294	CN_D292	CN_D288	CN_D252	CN_320
6	MIR4472-1	CN_328	CN_D318	CN_D296	CN_D254	CN_D224	CN_330
6	MIR4471	CN_D318	CN_D310	CN_D288	CN_D254	CN_D224	CN_330
6	MIR3925	CN_D296	CN_D292	CN_D288	CN_D253.2	CN_D252	CN_328
6	MIR378D2	CN_D224	CN_D316	CN_D254	CN_D253.2	CN_330	CN_328
6	MIR3610	CN_D318	CN_D288	CN_D254	CN_D224	CN_330	CN_328
6	MIR3151	CN_328	CN_D318	CN_D254	CN_D228	CN_D224	CN_330
6	MIR1278	CN_D310	CN_D294	CN_D292	CN_D288	CN_D252	CN_D224
6	MIR1234	CN_328	CN_D310	CN_D288	CN_D254	CN_D224	CN_330
6	MIR1205	CN_328	CN_D288	CN_D254	CN_D251	CN_D224	CN_330
6	MIR1204	CN_D224	CN_328	CN_D318	CN_D288	CN_D254	CN_330
6	MICB	CN_D296	CN_D292	CN_D288	CN_D253.2	CN_D252	CN_328
6	MICA	CN_D296	CN_D292	CN_D288	CN_D253.2	CN_D252	CN_328
6	MGC57346	ZS.DNA26	ZS.DNA11	CN_D306	CN_D296	CN_328	CN_324
6	MFSD3	CN_328	CN_D288	CN_D254	CN_D251	CN_D224	CN_330
6	MEX3A	CN_D294	CN_D292	CN_D252	CN_D251	CN_D227	CN_D224
6	MEF2D	CN_D294	CN_D292	CN_D288	CN_D253.2	CN_D252	CN_D224
6	MCCD1	CN_D296	CN_D292	CN_D288	CN_D253.2	CN_D252	CN_328
6	MAPT	ZS.DNA9	ZS.DNA25	CN_D306	CN_D296	CN_328	CN_324

6	MAL2	CN_D318	CN_D310	CN_D254	CN_D224	CN_330	CN_328
6	LY6E	CN_328	CN_D318	CN_D288	CN_D254	CN_D224	CN_330
6	LTB	CN_D296	CN_D292	CN_D288	CN_D253.2	CN_D252	CN_328
6	LTA	CN_D296	CN_D292	CN_D288	CN_D253.2	CN_D252	CN_328
6	LST1	CN_D296	CN_D292	CN_D288	CN_D253.2	CN_D252	CN_328
6	LRRC24	CN_328	CN_D288	CN_D254	CN_D251	CN_D224	CN_330
6	LRRC14	CN_328	CN_D288	CN_D254	CN_D251	CN_D224	CN_330
6	LRFN2	CN_D296	CN_D292	CN_D288	CN_D253.2	CN_D252	CN_328
6	LOC728724	CN_D318	CN_D310	CN_D254	CN_D224	CN_330	CN_328
6	LOC727677	CN_D224	CN_D318	CN_D288	CN_D254	CN_330	CN_328
6	LOC644172	ZS.DNA26	CN_D306	CN_D300	CN_D296	CN_328	CN_324
6	LOC392232	CN_D224	CN_D318	CN_D310	CN_D254	CN_330	CN_328
6	LOC339535	CN_D310	CN_D300	CN_D294	CN_D292	CN_D252	CN_320
6	LOC286184	CN_D224	CN_D318	CN_D310	CN_D288	CN_D254	CN_330
6	LOC255654	CN_D302.2	CN_D300	CN_D292	CN_D288	CN_D252	CN_330
6	LOC157381	CN_D224	CN_328	CN_D318	CN_D288	CN_D254	CN_330
6	LOC148696	CN_D302.2	CN_D294	CN_D292	CN_D288	CN_D252	CN_320
6	LOC100505918	CN_D310	CN_D294	CN_D292	CN_D288	CN_D252	CN_D224
6	LOC100500773	CN_D318	CN_D310	CN_D254	CN_D224	CN_330	CN_328
6	LOC100499183	CN_328	CN_D318	CN_D254	CN_D228	CN_D224	CN_330
6	LOC100133669	CN_328	CN_D318	CN_D288	CN_D254	CN_D224	CN_330
6	LOC100132354	CN_D296	CN_D292	CN_D288	CN_D253.2	CN_D252	CN_328
6	LOC100130331	CN_D300	CN_D294	CN_D288	CN_D253.2	CN_D252	CN_320
6	LOC100130301	CN_328	CN_D318	CN_D288	CN_D254	CN_D224	CN_330
6	LOC100130231	CN_D224	CN_D318	CN_D310	CN_D288	CN_D254	CN_330
6	LMNA	CN_D294	CN_D292	CN_D252	CN_D251	CN_D227	CN_D224
6	LINC00538	CN_D310	CN_D300	CN_D294	CN_D292	CN_D288	CN_D252
6	LINC00184	CN_D310	CN_D300	CN_D294	CN_D292	CN_D288	CN_D252
6	LINC00051	CN_328	CN_D318	CN_D296	CN_D254	CN_D224	CN_330
6	LAPTM4B	CN_D318	CN_D288	CN_D254	CN_D224	CN_330	CN_328
6	LAMTOR2	CN_D294	CN_D292	CN_D252	CN_D251	CN_D227	CN_D224
6	KIFC2	CN_328	CN_D288	CN_D254	CN_D251	CN_D224	CN_330
6	KCTD20	CN_D296	CN_D292	CN_D288	CN_D253.2	CN_D252	CN_328
6	KCNS2	CN_D318	CN_D310	CN_D254	CN_D224	CN_330	CN_328
6	KCNB2	CN_D224	CN_D318	CN_D310	CN_D254	CN_330	CN_328
6	ISG20L2	CN_D294	CN_D292	CN_D288	CN_D252	CN_D251	CN_D224
6	IQGAP3	CN_D294	CN_D292	CN_D288	CN_D253.2	CN_D252	CN_D224
6	INTS8	CN_D318	CN_D288	CN_D254	CN_D224	CN_330	CN_328
6	INPP4B	CN_D318	CN_D316	CN_D300	CN_D294	CN_D253.2	CN_D251
6	IMPA1	CN_328	CN_D318	CN_D310	CN_D254	CN_D224	CN_330
6	IL7	CN_D224	CN_D318	CN_D310	CN_D254	CN_330	CN_328
6	HSPA7	CN_D300	CN_D294	CN_D292	CN_D252	CN_D251	CN_D224
6	HSPA6	CN_D300	CN_D294	CN_D288	CN_D252	CN_D251	CN_D224

6	HPYR1	CN_D318	CN_D310	CN_D254	CN_D224	CN_330	CN_328
6	HLA-H	CN_D296	CN_D292	CN_D288	CN_D253.2	CN_D252	CN_328
6	HLA-DRB4	CN_D316	CN_D296	CN_D292	CN_D288	CN_D252	CN_328
6	HLA-DRA	CN_D296	CN_D292	CN_D288	CN_D252	CN_D224	CN_328
6	HLA-DPB2	CN_D292	CN_D288	CN_D253.2	CN_D252	CN_D224	CN_328
6	HLA-DOB	CN_D296	CN_D292	CN_D288	CN_D253.2	CN_D252	CN_328
6	HLA-C	CN_D296	CN_D292	CN_D288	CN_D253.2	CN_D252	CN_328
6	HLA-B	CN_D296	CN_D292	CN_D288	CN_D253.2	CN_D252	CN_328
6	HIVEP1	CN_D296	CN_D292	CN_D288	CN_D253.2	CN_D252	CN_328
6	HFE2	CN_D310	CN_D296	CN_D292	CN_D251	CN_D227	CN_D224
6	HCP5	CN_D296	CN_D292	CN_D288	CN_D253.2	CN_D252	CN_328
6	HCG4B	CN_D296	CN_D292	CN_D288	CN_D253.2	CN_D252	CN_328
6	HCG26	CN_D296	CN_D292	CN_D288	CN_D253.2	CN_D252	CN_328
6	HCG25	CN_D296	CN_D292	CN_D288	CN_D253.2	CN_D252	CN_328
6	HCG23	CN_D296	CN_D292	CN_D288	CN_D252	CN_D224	CN_328
6	HAPLN2	CN_D294	CN_D292	CN_D288	CN_D253.2	CN_D252	CN_D224
6	GRHL2	CN_328	CN_D318	CN_D310	CN_D254	CN_D224	CN_330
6	GPT	CN_328	CN_D288	CN_D254	CN_D251	CN_D224	CN_330
6	GPR20	CN_328	CN_D318	CN_D288	CN_D254	CN_D224	CN_330
6	GPR137B	CN_D300	CN_D294	CN_D292	CN_D288	CN_D252	CN_320
6	GPIHBP1	CN_328	CN_D318	CN_D310	CN_D254	CN_D224	CN_330
6	GPATCH4	CN_D294	CN_D292	CN_D288	CN_D253.2	CN_D252	CN_D224
6	GML	CN_328	CN_D318	CN_D254	CN_D251	CN_D224	CN_330
6	GLRX2	CN_D310	CN_D294	CN_D292	CN_D288	CN_D252	CN_D224
6	GGPS1	CN_D310	CN_D294	CN_D292	CN_D288	CN_D252	CN_320
6	FSTL4	CN_D300	CN_D254	CN_D253.2	CN_D224	CN_328	CN_324
6	FRS3	CN_D296	CN_D292	CN_D288	CN_D253.2	CN_D252	CN_328
6	FOXH1	CN_328	CN_D288	CN_D254	CN_D251	CN_D224	CN_330
6	FOXF2	CN_D296	CN_D292	CN_D288	CN_D253.2	CN_D252	CN_328
6	FMN2	CN_D310	CN_D302.2	CN_D294	CN_D292	CN_D288	CN_D252
6	FLVCR1	CN_D298	CN_D294	CN_D292	CN_D288	CN_D252	CN_320
6	FLVCR1-AS1	CN_D298	CN_D294	CN_D292	CN_D288	CN_D252	CN_320
6	FLJ43860	CN_328	CN_D318	CN_D288	CN_D254	CN_D224	CN_330
6	FGF14	CN_D318	CN_D302.2	CN_D298	CN_D296	CN_D253.2	CN_D251
6	FCGR2C	CN_D300	CN_D294	CN_D292	CN_D252	CN_D251	CN_D224
6	FCGR2A	CN_D300	CN_D294	CN_D288	CN_D252	CN_D251	CN_D224
6	FCER1G	CN_D310	CN_D300	CN_D294	CN_D292	CN_D252	CN_D224
6	FAM92A1	CN_D224	CN_D254	CN_D253.2	CN_D251	CN_330	CN_328
6	FAM71A	CN_D298	CN_D294	CN_D292	CN_D288	CN_D252	CN_320
6	FAM190A	CN_D300	CN_D253.2	CN_D251	CN_D224	CN_330	CN_320
6	FAM135B	CN_D224	CN_D318	CN_D310	CN_D254	CN_330	CN_328
6	FAM110B	CN_D224	CN_328	CN_D318	CN_D310	CN_D288	CN_D254
6	EIF3H	CN_D318	CN_D288	CN_D254	CN_D224	CN_330	CN_328

6	EIF3E	CN_328	CN_D310	CN_D288	CN_D254	CN_D224	CN_330
6	EFHC1	CN_D296	CN_D292	CN_D288	CN_D253.2	CN_D252	CN_328
6	EDARADD	CN_D310	CN_D294	CN_D292	CN_D288	CN_D252	CN_320
6	DSCAM	CN_D318	CN_D300	CN_D298	CN_D254	CN_D251	CN_328
6	DNAH14	CN_D310	CN_D300	CN_D294	CN_D292	CN_D252	CN_320
6	DDX39B	CN_D296	CN_D292	CN_D288	CN_D253.2	CN_D252	CN_328
6	DDAH2	CN_D296	CN_D292	CN_D288	CN_D253.2	CN_D252	CN_328
6	DCDC2	CN_D296	CN_D292	CN_D288	CN_D253.2	CN_D252	CN_D224
6	DCAF8	CN_D310	CN_D302.2	CN_D292	CN_D251	CN_D224	CN_320
6	DCAF6	CN_D296	CN_D294	CN_D292	CN_D288	CN_D252	CN_D224
6	DCAF4L2	CN_D224	CN_D318	CN_D288	CN_D254	CN_330	CN_328
6	DARS2	CN_D302.2	CN_D300	CN_D294	CN_D292	CN_D252	CN_D224
6	CYHR1	CN_328	CN_D288	CN_D254	CN_D251	CN_D224	CN_330
6	CPSF1	CN_328	CN_D310	CN_D288	CN_D254	CN_D224	CN_330
6	CPNE5	CN_D296	CN_D292	CN_D288	CN_D253.2	CN_D252	CN_328
6	CPA6	CN_328	CN_D318	CN_D296	CN_D254	CN_D224	CN_330
6	COMMD5	CN_328	CN_D288	CN_D254	CN_D251	CN_D224	CN_330
6	COL11A2	CN_D292	CN_D288	CN_D253.2	CN_D252	CN_D224	CN_328
6	CLIC1	CN_D296	CN_D292	CN_D288	CN_D253.2	CN_D252	CN_328
6	CHST8	CN_D316	CN_D310	CN_D298	CN_D294	CN_D253.2	CN_D251
6	CHD7	CN_D318	CN_D288	CN_D254	CN_D224	CN_330	CN_328
6	CFHR4	CN_D294	CN_D292	CN_D288	CN_D252	CN_D224	CN_320
6	CFHR3	CN_D294	CN_D292	CN_D288	CN_D252	CN_D224	CN_320
6	CFHR1	CN_D294	CN_D292	CN_D288	CN_D252	CN_D224	CN_320
6	CFH	CN_D310	CN_D294	CN_D292	CN_D288	CN_D252	CN_D224
6	CDKN1A	CN_D296	CN_D292	CN_D288	CN_D253.2	CN_D252	CN_328
6	CDH17	CN_D224	CN_D318	CN_D254	CN_D251	CN_330	CN_328
6	CDH12	CN_D224	CN_D298	CN_D296	CN_D294	CN_D288	CN_D225
6	CDC5L	CN_D296	CN_D292	CN_D288	CN_D253.2	CN_D252	CN_328
6	CDC42BPA	CN_D300	CN_D296	CN_D294	CN_D292	CN_D252	CN_320
6	CD34	CN_D302.2	CN_D294	CN_D292	CN_D288	CN_D252	CN_320
6	CCNE2	CN_D318	CN_D288	CN_D254	CN_D224	CN_330	CN_328
6	CAP2	CN_D296	CN_D292	CN_D288	CN_D253.2	CN_D252	CN_328
6	CALB1	CN_D224	CN_D318	CN_D288	CN_D254	CN_330	CN_328
6	CAGE1	CN_D296	CN_D292	CN_D288	CN_D253.2	CN_D252	CN_328
6	CACYBP	CN_D310	CN_D294	CN_D292	CN_D288	CN_D252	CN_D224
6	CA8	CN_D318	CN_D310	CN_D254	CN_D224	CN_330	CN_328
6	CA1	CN_D224	CN_D318	CN_D296	CN_D254	CN_330	CN_328
6	C8orf85	CN_D318	CN_D288	CN_D254	CN_D224	CN_330	CN_328
6	C8orf84	CN_D224	CN_328	CN_D318	CN_D310	CN_D254	CN_330
6	C8orf83	CN_D224	CN_D318	CN_D296	CN_D254	CN_330	CN_328
6	C8orf82	CN_328	CN_D288	CN_D254	CN_D251	CN_D224	CN_330
6	C8orf56	CN_328	CN_D318	CN_D254	CN_D228	CN_D224	CN_330

6	C8orf46	CN_328	CN_D318	CN_D310	CN_D254	CN_D224	CN_330
6	C8orf39	CN_D224	CN_D254	CN_D253.2	CN_D251	CN_330	CN_328
6	C8orf38	CN_D318	CN_D288	CN_D254	CN_D224	CN_330	CN_328
6	C8orf31	CN_328	CN_D318	CN_D288	CN_D254	CN_D224	CN_330
6	C6orf25	CN_D296	CN_D292	CN_D288	CN_D253.2	CN_D252	CN_328
6	C1orf61	CN_D292	CN_D288	CN_D253.2	CN_D252	CN_D251	CN_D224
6	C1orf27	CN_D300	CN_D294	CN_D292	CN_D288	CN_D252	CN_D224
6	C1orf227	CN_D298	CN_D294	CN_D292	CN_D288	CN_D252	CN_320
6	C1orf192	CN_D310	CN_D292	CN_D288	CN_D253.2	CN_D252	CN_D224
6	C1orf129	CN_D310	CN_D294	CN_D292	CN_D252	CN_D224	CN_320
6	C1orf111	CN_D310	CN_D294	CN_D292	CN_D288	CN_D252	CN_D224
6	C1orf110	CN_D294	CN_D292	CN_D288	CN_D252	CN_D251	CN_D224
6	C1orf101	CN_D310	CN_D302.2	CN_D300	CN_D292	CN_D252	CN_320
6	C17orf69	ZS.DNA26	ZS.DNA11	CN_D306	CN_D296	CN_328	CN_324
6	BTNL2	CN_D296	CN_D292	CN_D288	CN_D252	CN_D224	CN_328
6	BATF3	CN_D298	CN_D294	CN_D292	CN_D288	CN_D252	CN_320
6	BANP	CN_D306	CN_D253.2	CN_D225	CN_328	CN_324	CN_320
6	B4GALT3	CN_D310	CN_D300	CN_D294	CN_D292	CN_D252	CN_D251
6	B3GALT2	CN_D310	CN_D294	CN_D292	CN_D288	CN_D252	CN_D224
6	B3GALNT2	CN_D310	CN_D302.2	CN_D300	CN_D294	CN_D292	CN_D252
6	ATP6V1G2-DD	CN_D296	CN_D292	CN_D288	CN_D253.2	CN_D252	CN_328
6	ATP6V1G2	CN_D296	CN_D292	CN_D288	CN_D253.2	CN_D252	CN_328
6	ATP6V1C1	CN_328	CN_D318	CN_D296	CN_D254	CN_D224	CN_330
6	ATF6	CN_D310	CN_D292	CN_D288	CN_D252	CN_D251	CN_D224
6	ATF3	CN_D298	CN_D294	CN_D292	CN_D288	CN_D252	CN_320
6	ASH1L	CN_D310	CN_D294	CN_D252	CN_D251	CN_D227	CN_D224
6	ASAP1-IT1	CN_D318	CN_D296	CN_D254	CN_D224	CN_330	CN_328
6	ARL17B	CN_D306	CN_D296	CN_D251	CN_328	CN_324	CN_320
6	ARHGEF2	CN_D294	CN_D292	CN_D252	CN_D251	CN_D227	CN_D224
6	ARHGAP39	CN_328	CN_D288	CN_D254	CN_D251	CN_D224	CN_330
6	APOA2	CN_D310	CN_D300	CN_D294	CN_D292	CN_D252	CN_D224
6	APOA1BP	CN_D294	CN_D292	CN_D288	CN_D253.2	CN_D252	CN_D224
6	ANXA13	CN_D224	CN_328	CN_D318	CN_D296	CN_D254	CN_330
6	ANKRD36BP1	CN_D310	CN_D302.2	CN_D292	CN_D288	CN_D252	CN_D224
6	ADHFE1	CN_328	CN_D318	CN_D310	CN_D254	CN_D224	CN_330
6	ADCY10	CN_D302.2	CN_D298	CN_D294	CN_D292	CN_D252	CN_D224
6	ADAMTS12	CN_D224	CN_D298	CN_D296	CN_D288	CN_D253.2	CN_D225
6	ACCN1	ZS.DNA25	ZS.DNA24	CN_D304	CN_D296	CN_D251	CN_D224
5	ZP4	CN_D294	CN_D288	CN_D253.2	CN_D252	CN_320	
5	ZNF704	CN_328	CN_D318	CN_D254	CN_D224	CN_330	
5	ZNF696	CN_328	CN_D318	CN_D254	CN_D224	CN_330	
5	ZNF623	CN_328	CN_D318	CN_D254	CN_D224	CN_330	
5	ZNF311	CN_D292	CN_D288	CN_D253.2	CN_D252	CN_328	

5	ZNF238	CN_D294	CN_D292	CN_D288	CN_D252	CN_320
5	ZNF187	CN_D292	CN_D288	CN_D252	CN_D224	CN_328
5	ZNF16	CN_D288	CN_D254	CN_D251	CN_D224	CN_330
5	ZKSCAN4	CN_D292	CN_D288	CN_D252	CN_D224	CN_328
5	ZHX1	CN_328	CN_D288	CN_D254	CN_D224	CN_330
5	ZHX1-C8ORF76	CN_328	CN_D288	CN_D254	CN_D224	CN_330
5	ZFR	CN_D224	CN_D298	CN_D296	CN_D288	CN_D225
5	ZFAND1	CN_328	CN_D318	CN_D254	CN_D224	CN_330
5	ZDHHC14	CN_D298	CN_D296	CN_D288	CN_D254	CN_D251
5	ZC3H3	CN_328	CN_D318	CN_D254	CN_D224	CN_330
5	ZC2HC1A	CN_D224	CN_D318	CN_D310	CN_D254	CN_328
5	ZBTB41	CN_D310	CN_D294	CN_D292	CN_D288	CN_D252
5	ZBTB37	CN_D302.2	CN_D300	CN_D292	CN_D252	CN_D224
5	ZBTB22	CN_D292	CN_D288	CN_D253.2	CN_D252	CN_328
5	ZBTB12	CN_D292	CN_D288	CN_D253.2	CN_D252	CN_328
5	YTHDF3	CN_D224	CN_D310	CN_D254	CN_330	CN_328
5	XPR1	CN_D298	CN_D294	CN_D292	CN_D252	CN_D224
5	XPO7	CN_D298	CN_D253.2	CN_D227	CN_328	CN_324
5	XCL2	CN_D294	CN_D292	CN_D288	CN_D252	CN_D224
5	XCL1	CN_D294	CN_D292	CN_D288	CN_D252	CN_D224
5	WDR70	CN_D224	CN_D298	CN_D296	CN_D253.2	CN_D225
5	WDR46	CN_D292	CN_D288	CN_D253.2	CN_D252	CN_328
5	VWA7	CN_D292	CN_D288	CN_D253.2	CN_D252	CN_328
5	VARS	CN_D292	CN_D288	CN_D253.2	CN_D252	CN_328
5	VARS2	CN_D292	CN_D288	CN_D253.2	CN_D252	CN_328
5	VAMP4	CN_D296	CN_D292	CN_D252	CN_D224	CN_320
5	USP21	CN_D310	CN_D300	CN_D292	CN_D252	CN_D251
5	USF2	CN_D316	CN_D310	CN_D298	CN_D294	CN_D251
5	UGT3A2	CN_D224	CN_D298	CN_D288	CN_D252	CN_D225
5	UFC1	CN_D310	CN_D300	CN_D292	CN_D252	CN_D251
5	UBE2Q1	CN_D300	CN_D294	CN_D251	CN_D227	CN_328
5	TUSC5	ZS.DNA26	CN_D306	CN_D300	CN_328	CN_324
5	TUBB2B	CN_D292	CN_D288	CN_D253.2	CN_D252	CN_328
5	TSTA3	CN_328	CN_D318	CN_D254	CN_D224	CN_330
5	TSPYL5	CN_D318	CN_D254	CN_D224	CN_330	CN_328
5	TRMT1L	CN_D294	CN_D292	CN_D288	CN_D252	CN_D224
5	TRMT12	CN_D224	CN_328	CN_D318	CN_D254	CN_330
5	TRIM55	CN_328	CN_D318	CN_D254	CN_D224	CN_330
5	TRIM39-RPP21	CN_D292	CN_D288	CN_D253.2	CN_D252	CN_328
5	TRIM39	CN_D292	CN_D288	CN_D253.2	CN_D252	CN_328
5	TRIM27	CN_D292	CN_D288	CN_D253.2	CN_D252	CN_328
5	TRIM17	CN_D310	CN_D292	CN_D288	CN_D252	CN_320
5	TRIM11	CN_D310	CN_D292	CN_D288	CN_D252	CN_320

5	TRIB1	CN_D224	CN_328	CN_D318	CN_D254	CN_330
5	TRAF5	CN_D310	CN_D302.2	CN_D298	CN_D292	CN_D252
5	TPR	CN_D294	CN_D292	CN_D288	CN_D252	CN_D224
5	TPK1	CN_D225	CN_D316	CN_D292	CN_D253.2	CN_D224
5	TOR1AIP2	CN_D310	CN_D296	CN_D292	CN_D252	CN_D224
5	TOP1MT	CN_328	CN_D318	CN_D254	CN_D224	CN_330
5	TOMM40L	CN_D310	CN_D300	CN_D292	CN_D252	CN_D224
5	TOMM20	CN_D294	CN_D292	CN_D288	CN_D252	CN_320
5	TNXB	CN_D292	CN_D288	CN_D253.2	CN_D252	CN_328
5	TNXA	CN_D292	CN_D288	CN_D253.2	CN_D252	CN_328
5	TMTC4	CN_D318	CN_D302.2	CN_D296	CN_D253.2	CN_D251
5	TMEM74	CN_D310	CN_D254	CN_D251	CN_D224	CN_330
5	TMEM71	CN_328	CN_D318	CN_D254	CN_D224	CN_330
5	TMEM63A	CN_D302.2	CN_D296	CN_D292	CN_D288	CN_D252
5	TMEM217	CN_D296	CN_D292	CN_D288	CN_D252	CN_328
5	TMEM151B	CN_D292	CN_D288	CN_D253.2	CN_D252	CN_328
5	TMEM14A	CN_D296	CN_D292	CN_D288	CN_D252	CN_328
5	TMCC2	CN_D310	CN_D292	CN_D288	CN_D252	CN_320
5	TINAG	CN_D296	CN_D292	CN_D288	CN_D253.2	CN_D252
5	TIGD5	CN_328	CN_D318	CN_D254	CN_D224	CN_330
5	TFEB	CN_D296	CN_D292	CN_D288	CN_D252	CN_328
5	TERF1	CN_D224	CN_D318	CN_D310	CN_D254	CN_330
5	TDRD5	CN_D316	CN_D296	CN_D292	CN_D252	CN_D224
5	TDRD10	CN_D300	CN_D294	CN_D251	CN_D227	CN_328
5	TCTE1	CN_D292	CN_D288	CN_D253.2	CN_D252	CN_328
5	TCP11	CN_D292	CN_D288	CN_D252	CN_D224	CN_328
5	TCF19	CN_D292	CN_D288	CN_D253.2	CN_D252	CN_328
5	TBC1D22B	CN_D296	CN_D292	CN_D288	CN_D252	CN_328
5	TATDN1	CN_D224	CN_328	CN_D318	CN_D254	CN_330
5	TAPBP	CN_D292	CN_D288	CN_D253.2	CN_D252	CN_328
5	SYT2	CN_D300	CN_D298	CN_D292	CN_D252	CN_D224
5	SYBU	CN_D318	CN_D254	CN_D224	CN_330	CN_328
5	SWT1	CN_D294	CN_D292	CN_D288	CN_D252	CN_D224
5	SV2A	CN_D300	CN_D288	CN_D227	CN_D224	CN_328
5	STK19	CN_D292	CN_D288	CN_D253.2	CN_D252	CN_328
5	STH	ZS.DNA25	CN_D306	CN_D296	CN_328	CN_324
5	ST18	CN_328	CN_D318	CN_D310	CN_D288	CN_D224
5	SSR1	CN_D292	CN_D288	CN_D253.2	CN_D252	CN_328
5	SPPL2C	CN_D306	CN_D296	CN_D224	CN_328	CN_324
5	SPDEF	CN_D292	CN_D288	CN_D252	CN_D224	CN_328
5	SOX4	CN_D296	CN_D292	CN_D288	CN_D252	CN_328
5	SOX17	CN_D224	CN_328	CN_D318	CN_D288	CN_320
5	SNX16	CN_328	CN_D318	CN_D254	CN_D224	CN_330

5	SNRNP48	CN_D292	CN_D288	CN_D253.2	CN_D252	CN_328
5	SNORD81	CN_D302.2	CN_D300	CN_D292	CN_D252	CN_D224
5	SNORD80	CN_D302.2	CN_D300	CN_D292	CN_D252	CN_D224
5	SNORD79	CN_D302.2	CN_D300	CN_D292	CN_D252	CN_D224
5	SNORD78	CN_D302.2	CN_D300	CN_D292	CN_D252	CN_D224
5	SNORD77	CN_D302.2	CN_D300	CN_D292	CN_D252	CN_D224
5	SNORD76	CN_D302.2	CN_D300	CN_D292	CN_D252	CN_D224
5	SNORD75	CN_D302.2	CN_D300	CN_D292	CN_D252	CN_D224
5	SNORD74	CN_D302.2	CN_D300	CN_D292	CN_D252	CN_D224
5	SNORD52	CN_D292	CN_D288	CN_D253.2	CN_D252	CN_328
5	SNORD48	CN_D292	CN_D288	CN_D253.2	CN_D252	CN_328
5	SNORD47	CN_D302.2	CN_D300	CN_D292	CN_D252	CN_D224
5	SNORD44	CN_D302.2	CN_D300	CN_D292	CN_D252	CN_D224
5	SNORD32B	CN_D292	CN_D288	CN_D252	CN_D224	CN_328
5	SNORA72	CN_D318	CN_D254	CN_D224	CN_330	CN_328
5	SNORA38	CN_D296	CN_D292	CN_D288	CN_D252	CN_328
5	SNORA14B	CN_D294	CN_D292	CN_D288	CN_D252	CN_320
5	SNCAIP	CN_D254	CN_D253.2	CN_D252	CN_D224	CN_320
5	SMG7	CN_D302.2	CN_D300	CN_D292	CN_D252	CN_D224
5	SLURP1	CN_328	CN_D318	CN_D254	CN_D224	CN_330
5	SLIT3	CN_D298	CN_D296	CN_D254	CN_D253.2	CN_324
5	SLC9A8	CN_D318	CN_D298	CN_D296	CN_D253.2	CN_320
5	SLC44A4	CN_D292	CN_D288	CN_D253.2	CN_D252	CN_328
5	SLC35F3	CN_D300	CN_D294	CN_D292	CN_D288	CN_D252
5	SLC2A14	CN_D316	CN_D306	CN_D228	CN_330	CN_324
5	SLC22A4	CN_D316	CN_D298	CN_D254	CN_D253.2	CN_328
5	SLC22A23	CN_D292	CN_D288	CN_D253.2	CN_D252	CN_328
5	SLC1A3	CN_D224	CN_D296	CN_D288	CN_D253.2	CN_D225
5	SLC12A7	CN_D225	CN_D302.2	CN_D296	CN_D254	CN_328
5	SLAMF1	CN_D310	CN_D300	CN_D252	CN_D251	CN_D224
5	SKP2	CN_D224	CN_D298	CN_D288	CN_D252	CN_D225
5	SKIV2L	CN_D292	CN_D288	CN_D253.2	CN_D252	CN_328
5	SIRT5	CN_D296	CN_D292	CN_D288	CN_D252	CN_328
5	SIPA1L2	CN_D310	CN_D300	CN_D296	CN_D252	CN_320
5	SGK3	CN_328	CN_D318	CN_D254	CN_D224	CN_330
5	SF3B4	CN_D300	CN_D288	CN_D227	CN_D224	CN_328
5	SERPINB9	CN_D296	CN_D292	CN_D288	CN_D252	CN_328
5	SERPINB6	CN_D296	CN_D292	CN_D288	CN_D252	CN_328
5	SCXB	CN_328	CN_D310	CN_D254	CN_D224	CN_330
5	SCXA	CN_328	CN_D310	CN_D254	CN_D224	CN_330
5	SCRT1	CN_328	CN_D310	CN_D254	CN_D224	CN_330
5	SCGN	CN_D292	CN_D288	CN_D252	CN_D224	CN_328
5	SCAND3	CN_D292	CN_D288	CN_D253.2	CN_D252	CN_328

5	SBF1P1	CN_D224	CN_328	CN_D318	CN_D310	CN_D288
5	SAPCD1	CN_D292	CN_D288	CN_D253.2	CN_D252	CN_328
5	SAMD4A	ZS.DNA24	CN_D254	CN_D253.2	CN_D251	CN_320
5	RXFP4	CN_D294	CN_D292	CN_D252	CN_D227	CN_D224
5	RUFY1	ZS.DNA24	CN_D288	CN_D254	CN_D253.2	CN_328
5	RRS1	CN_328	CN_D318	CN_D254	CN_D224	CN_330
5	RRP15	CN_D296	CN_D294	CN_D292	CN_D288	CN_D252
5	RPS6KA2	CN_D298	CN_D296	CN_D288	CN_D253.2	CN_D251
5	RPS18	CN_D292	CN_D288	CN_D253.2	CN_D252	CN_328
5	RPL30	CN_D318	CN_D254	CN_D224	CN_330	CN_328
5	RNU5F-1	CN_D310	CN_D294	CN_D292	CN_D253.2	CN_D252
5	RNF2	CN_D294	CN_D292	CN_D288	CN_D252	CN_D224
5	RNF187	CN_D310	CN_D292	CN_D288	CN_D252	CN_320
5	RNF139	CN_D224	CN_328	CN_D318	CN_D254	CN_330
5	RHPN1	CN_328	CN_D318	CN_D254	CN_D224	CN_330
5	RHBG	CN_D292	CN_D253.2	CN_D252	CN_D251	CN_D224
5	RHAG	CN_D296	CN_D292	CN_D288	CN_D252	CN_328
5	RGS20	CN_D224	CN_328	CN_D318	CN_D310	CN_D288
5	RGL2	CN_D292	CN_D288	CN_D253.2	CN_D252	CN_328
5	RGL1	CN_D310	CN_D302.2	CN_D253.2	CN_D252	CN_D224
5	REXO1L2P	CN_D224	CN_D318	CN_D254	CN_330	CN_328
5	REXO1L1	CN_D224	CN_D318	CN_D254	CN_330	CN_328
5	RDBP	CN_D292	CN_D288	CN_D253.2	CN_D252	CN_328
5	RCOR3	CN_D310	CN_D302.2	CN_D298	CN_D292	CN_D252
5	RCAN2	CN_D296	CN_D292	CN_D288	CN_D252	CN_328
5	RBM34	CN_D294	CN_D292	CN_D288	CN_D252	CN_320
5	RANBP9	CN_D296	CN_D292	CN_D288	CN_D252	CN_328
5	PYHIN1	CN_D292	CN_D252	CN_D251	CN_D224	CN_320
5	PYCRL	CN_328	CN_D318	CN_D254	CN_D224	CN_330
5	PXT1	CN_D296	CN_D292	CN_D288	CN_D252	CN_328
5	PXDC1	CN_D296	CN_D292	CN_D252	CN_D224	CN_328
5	PTPN14	CN_D300	CN_D294	CN_D292	CN_D288	CN_D252
5	PTP4A3	CN_328	CN_D318	CN_D288	CN_D254	CN_D224
5	PTGS2	CN_D310	CN_D294	CN_D288	CN_D252	CN_D224
5	PSORS1C3	CN_D292	CN_D288	CN_D253.2	CN_D252	CN_328
5	PSMG4	CN_D292	CN_D288	CN_D253.2	CN_D252	CN_328
5	PSCA	CN_328	CN_D318	CN_D254	CN_D224	CN_330
5	PRRC2A	CN_D296	CN_D292	CN_D288	CN_D252	CN_328
5	PREX2	CN_D318	CN_D310	CN_D254	CN_D224	CN_328
5	PPP1R18	CN_D296	CN_D292	CN_D288	CN_D252	CN_328
5	PPP1R12B	CN_D300	CN_D298	CN_D292	CN_D252	CN_D224
5	PPP1R10	CN_D296	CN_D292	CN_D288	CN_D252	CN_328
5	PPIL1	CN_D292	CN_D288	CN_D253.2	CN_D252	CN_328

5	POU5F1	CN_D292	CN_D288	CN_D253.2	CN_D252	CN_328
5	POU5F1B	CN_D224	CN_D318	CN_D254	CN_330	CN_328
5	POP1	CN_D318	CN_D254	CN_D224	CN_330	CN_328
5	PMP2	CN_328	CN_D318	CN_D254	CN_D224	CN_330
5	PLEKHO1	CN_D300	CN_D292	CN_D288	CN_D227	CN_D224
5	PLEKHF2	CN_D318	CN_D254	CN_D224	CN_330	CN_328
5	PKIA	CN_D224	CN_D318	CN_D310	CN_D254	CN_328
5	PKHD1	CN_D296	CN_D292	CN_D288	CN_D253.2	CN_328
5	PIP5K1P1	CN_D292	CN_D288	CN_D252	CN_D224	CN_328
5	PIM1	CN_D296	CN_D292	CN_D288	CN_D252	CN_328
5	PI16	CN_D296	CN_D292	CN_D288	CN_D252	CN_328
5	PHF20L1	CN_328	CN_D318	CN_D254	CN_D224	CN_330
5	PHACTR1	CN_D296	CN_D292	CN_D288	CN_D253.2	CN_D252
5	PGC	CN_D296	CN_D292	CN_D288	CN_D252	CN_328
5	PGBD1	CN_D292	CN_D288	CN_D252	CN_D224	CN_328
5	PFDN6	CN_D292	CN_D288	CN_D253.2	CN_D252	CN_328
5	PFDN2	CN_D310	CN_D302.2	CN_D300	CN_D292	CN_D251
5	PEX19	CN_D310	CN_D294	CN_D292	CN_D251	CN_D224
5	PDP2	CN_D298	CN_D296	CN_D251	CN_328	CN_320
5	PDC	CN_D300	CN_D294	CN_D288	CN_D252	CN_D224
5	PCP4L1	CN_D310	CN_D292	CN_D253.2	CN_D252	CN_D224
5	PAQR8	CN_D296	CN_D292	CN_D288	CN_D252	CN_328
5	PACSIN1	CN_D292	CN_D288	CN_D252	CN_D224	CN_328
5	P4HA2	CN_D298	CN_D254	CN_D253.2	CN_D224	CN_328
5	OTUD7B	CN_D300	CN_D288	CN_D227	CN_D224	CN_328
5	OR2W1	CN_D292	CN_D288	CN_D253.2	CN_D252	CN_328
5	OR2J3	CN_D292	CN_D288	CN_D253.2	CN_D252	CN_328
5	OR2H2	CN_D292	CN_D288	CN_D252	CN_D224	CN_328
5	OR2B3	CN_D292	CN_D288	CN_D253.2	CN_D252	CN_328
5	ODZ2	CN_D298	CN_D296	CN_D254	CN_D253.2	CN_D224
5	NXN	ZS.DNA9	ZS.DNA25	CN_D306	CN_328	CN_324
5	NVL	CN_D310	CN_D292	CN_D288	CN_D252	CN_320
5	NUCKS1	CN_D300	CN_D294	CN_D292	CN_D288	CN_D252
5	NSFP1	CN_D306	CN_D296	CN_D251	CN_328	CN_324
5	NRSN1	CN_D296	CN_D292	CN_D288	CN_D253.2	CN_D252
5	NRM	CN_D296	CN_D292	CN_D288	CN_D252	CN_328
5	NR5A2	CN_D300	CN_D294	CN_D292	CN_D252	CN_320
5	NR1I3	CN_D310	CN_D300	CN_D292	CN_D252	CN_D224
5	NPM2	CN_D298	CN_D253.2	CN_D227	CN_328	CN_324
5	NPL	CN_D300	CN_D292	CN_D288	CN_D252	CN_D224
5	NPAS3	CN_D300	CN_D298	CN_D254	CN_D253.2	CN_324
5	NOV	CN_D318	CN_D254	CN_D224	CN_330	CN_328
5	NOL7	CN_D296	CN_D292	CN_D288	CN_D252	CN_328

5	NKAPL	CN_D292	CN_D288	CN_D252	CN_D224	CN_328
5	NIPBL	CN_D224	CN_D296	CN_D288	CN_D252	CN_D225
5	NEU1	CN_D292	CN_D288	CN_D253.2	CN_D252	CN_328
5	NES	CN_D294	CN_D292	CN_D288	CN_D252	CN_D224
5	NDUFB9	CN_D224	CN_328	CN_D318	CN_D254	CN_330
5	NCSTN	CN_D296	CN_D294	CN_D288	CN_D252	CN_D224
5	NCF2	CN_D302.2	CN_D292	CN_D253.2	CN_D252	CN_D224
5	NAPRT1	CN_328	CN_D318	CN_D254	CN_D224	CN_330
5	MYO16	CN_D318	CN_D302.2	CN_D296	CN_D253.2	CN_D251
5	MYLK4	CN_D292	CN_D288	CN_D253.2	CN_D252	CN_328
5	MUTED-TXND(	CN_D292	CN_D288	CN_D252	CN_D224	CN_328
5	MUTED	CN_D292	CN_D288	CN_D252	CN_D224	CN_328
5	MUT	CN_D296	CN_D292	CN_D288	CN_D252	CN_328
5	MTMR12	CN_D224	CN_D296	CN_D288	CN_D253.2	CN_D225
5	MTMR11	CN_D300	CN_D288	CN_D227	CN_D224	CN_328
5	MTCH1	CN_D296	CN_D292	CN_D288	CN_D252	CN_328
5	MRPS18B	CN_D296	CN_D292	CN_D288	CN_D252	CN_328
5	MPZL1	CN_D310	CN_D302.2	CN_D292	CN_D252	CN_D224
5	MOG	CN_D292	CN_D288	CN_D252	CN_D224	CN_328
5	MLIP	CN_D296	CN_D292	CN_D288	CN_D253.2	CN_D252
5	MIR939	CN_328	CN_D310	CN_D254	CN_D224	CN_330
5	MIR921	CN_D310	CN_D292	CN_D288	CN_D252	CN_D224
5	MIR877	CN_D296	CN_D292	CN_D288	CN_D252	CN_328
5	MIR580	CN_D224	CN_D298	CN_D288	CN_D252	CN_D225
5	MIR556	CN_D294	CN_D292	CN_D288	CN_D252	CN_D224
5	MIR488	CN_D296	CN_D294	CN_D292	CN_D252	CN_D224
5	MIR4666A	CN_D310	CN_D292	CN_D288	CN_D252	CN_320
5	MIR4664	CN_328	CN_D318	CN_D254	CN_D224	CN_330
5	MIR4663	CN_328	CN_D288	CN_D254	CN_D224	CN_330
5	MIR4646	CN_D296	CN_D292	CN_D288	CN_D252	CN_328
5	MIR4645	CN_D296	CN_D292	CN_D288	CN_D252	CN_328
5	MIR4642	CN_D292	CN_D288	CN_D253.2	CN_D252	CN_328
5	MIR4641	CN_D296	CN_D292	CN_D288	CN_D252	CN_328
5	MIR4640	CN_D292	CN_D288	CN_D253.2	CN_D252	CN_328
5	MIR4635	CN_D225	CN_D302.2	CN_D296	CN_D254	CN_328
5	MIR4257	CN_D292	CN_D288	CN_D227	CN_D224	CN_320
5	MIR320B2	CN_D310	CN_D292	CN_D288	CN_D252	CN_320
5	MIR3150B	CN_D318	CN_D254	CN_D224	CN_330	CN_328
5	MIR3150A	CN_D318	CN_D254	CN_D224	CN_330	CN_328
5	MIR205HG	CN_D294	CN_D292	CN_D288	CN_D252	CN_320
5	MIR205	CN_D294	CN_D292	CN_D288	CN_D252	CN_320
5	MIR1275	CN_D292	CN_D288	CN_D253.2	CN_D252	CN_328
5	MIR1236	CN_D292	CN_D288	CN_D253.2	CN_D252	CN_328

5	MGC39372	CN_D296	CN_D292	CN_D288	CN_D252	CN_328
5	MFSD4	CN_D300	CN_D294	CN_D288	CN_D252	CN_320
5	METTL13	CN_D296	CN_D292	CN_D252	CN_D224	CN_320
5	MED30	CN_D310	CN_D254	CN_D224	CN_330	CN_328
5	MDGA2	CN_D300	CN_D292	CN_D254	CN_D253.2	CN_D251
5	MDGA1	CN_D292	CN_D288	CN_D253.2	CN_D252	CN_328
5	MDFI	CN_D296	CN_D292	CN_D288	CN_D252	CN_328
5	MDC1	CN_D296	CN_D292	CN_D288	CN_D252	CN_328
5	MCL1	CN_D292	CN_D288	CN_D227	CN_D224	CN_320
5	MBOAT1	CN_D296	CN_D292	CN_D288	CN_D253.2	CN_D252
5	MATN2	CN_D318	CN_D254	CN_D224	CN_330	CN_328
5	MARK1	CN_D310	CN_D300	CN_D292	CN_D288	CN_D252
5	MAPT-AS1	CN_D306	CN_D296	CN_D224	CN_328	CN_324
5	MAPKAPK2	CN_D318	CN_D310	CN_D294	CN_D292	CN_D252
5	MAPK15	CN_328	CN_D318	CN_D254	CN_D224	CN_330
5	MAK	CN_D296	CN_D292	CN_D288	CN_D252	CN_328
5	MAG	CN_D316	CN_D310	CN_D298	CN_D294	CN_D251
5	MAFA	CN_328	CN_D318	CN_D254	CN_D224	CN_330
5	LYPD2	CN_328	CN_D318	CN_D254	CN_D224	CN_330
5	LYNX1	CN_328	CN_D318	CN_D254	CN_D224	CN_330
5	LY86-AS1	CN_D296	CN_D292	CN_D288	CN_D252	CN_328
5	LY6K	CN_328	CN_D318	CN_D254	CN_D224	CN_330
5	LY6G6F	CN_D296	CN_D292	CN_D288	CN_D252	CN_328
5	LY6G6E	CN_D296	CN_D292	CN_D288	CN_D252	CN_328
5	LY6G6D	CN_D296	CN_D292	CN_D288	CN_D252	CN_328
5	LY6G6C	CN_D296	CN_D292	CN_D288	CN_D252	CN_328
5	LY6G5C	CN_D296	CN_D292	CN_D288	CN_D252	CN_328
5	LY6G5B	CN_D296	CN_D292	CN_D288	CN_D252	CN_328
5	LY6D	CN_328	CN_D318	CN_D254	CN_D224	CN_330
5	LSR	CN_D316	CN_D310	CN_D298	CN_D294	CN_D251
5	LSM2	CN_D292	CN_D288	CN_D253.2	CN_D252	CN_328
5	LRRCC1	CN_D224	CN_D318	CN_D254	CN_330	CN_328
5	LRRC37A	CN_D306	CN_D296	CN_328	CN_324	CN_320
5	LRRC37A4	CN_D316	CN_D306	CN_D251	CN_328	CN_324
5	LRRC16A	CN_D292	CN_D288	CN_D252	CN_D224	CN_328
5	LPGAT1	CN_D318	CN_D310	CN_D294	CN_D292	CN_D252
5	LOC731779	CN_328	CN_D318	CN_D254	CN_D224	CN_330
5	LOC730101	CN_D296	CN_D292	CN_D288	CN_D252	CN_328
5	LOC728463	CN_D296	CN_D294	CN_D292	CN_D288	CN_D252
5	LOC646719	CN_D224	CN_D296	CN_D288	CN_D252	CN_D225
5	LOC645676	CN_D294	CN_D252	CN_D251	CN_D227	CN_D224
5	LOC553103	CN_D316	CN_D298	CN_D254	CN_D253.2	CN_328
5	LOC401242	CN_D292	CN_D288	CN_D253.2	CN_D252	CN_328

5	LOC400794	CN_D310	CN_D294	CN_D252	CN_D224	CN_320
5	LOC284581	CN_D310	CN_D294	CN_D292	CN_D288	CN_D252
5	LOC284578	CN_D300	CN_D294	CN_D288	CN_D252	CN_320
5	LOC283867	CN_D306	CN_D251	CN_328	CN_324	CN_320
5	LOC100506046	CN_D302.2	CN_D300	CN_D292	CN_D252	CN_D224
5	LOC100505676	CN_328	CN_D318	CN_D254	CN_D224	CN_330
5	LOC100505659	CN_328	CN_D318	CN_D254	CN_D224	CN_330
5	LOC100293534	CN_D292	CN_D288	CN_D253.2	CN_D252	CN_328
5	LOC100288748	CN_D316	CN_D254	CN_D224	CN_330	CN_328
5	LOC100288181	CN_328	CN_D318	CN_D254	CN_D224	CN_330
5	LOC100288079	CN_D294	CN_D292	CN_D288	CN_D252	CN_D224
5	LOC100131726	CN_328	CN_D288	CN_D254	CN_D224	CN_330
5	LOC100131047	CN_D296	CN_D292	CN_D288	CN_D253.2	CN_D252
5	LOC100129636	CN_D292	CN_D288	CN_D253.2	CN_D252	CN_328
5	LOC100128338	CN_328	CN_D318	CN_D254	CN_D224	CN_330
5	LOC100128126	CN_328	CN_D318	CN_D254	CN_D224	CN_330
5	LMX1A	CN_D300	CN_D296	CN_D294	CN_D252	CN_D224
5	LMBRD2	CN_D224	CN_D298	CN_D288	CN_D252	CN_D225
5	LINC00467	CN_D310	CN_D302.2	CN_D298	CN_D292	CN_D252
5	LINC00340	CN_D296	CN_D292	CN_D288	CN_D252	CN_328
5	LINC00313	CN_D318	CN_D298	CN_D254	CN_328	CN_324
5	LINC00293	CN_D318	CN_D288	CN_D254	CN_D253.2	CN_D251
5	LHX9	CN_D296	CN_D294	CN_D292	CN_D252	CN_D224
5	LEFTY2	CN_D302.2	CN_D300	CN_D292	CN_D288	CN_D252
5	LEFTY1	CN_D302.2	CN_D296	CN_D292	CN_D288	CN_D252
5	KRBA1	ZS.DNA26	CN_D300	CN_D292	CN_D253.2	CN_D225
5	KLHL38	CN_D224	CN_328	CN_D296	CN_D254	CN_330
5	KIFC1	CN_D292	CN_D288	CN_D253.2	CN_D252	CN_328
5	KIF6	CN_D292	CN_D288	CN_D253.2	CN_D252	CN_328
5	KIF14	CN_D294	CN_D292	CN_D253.2	CN_D252	CN_320
5	KIF13A	CN_D296	CN_D292	CN_D288	CN_D252	CN_328
5	KIAA1429	CN_D318	CN_D254	CN_D224	CN_330	CN_328
5	KHDRBS3	CN_D318	CN_D254	CN_D224	CN_330	CN_328
5	KCNK5	CN_D292	CN_D288	CN_D253.2	CN_D252	CN_328
5	KCNK1	CN_D296	CN_D294	CN_D288	CN_D252	CN_320
5	KCNK17	CN_D292	CN_D288	CN_D253.2	CN_D252	CN_328
5	KCNK16	CN_D292	CN_D288	CN_D253.2	CN_D252	CN_328
5	JPH1	CN_328	CN_D318	CN_D254	CN_D224	CN_330
5	JARID2	CN_D316	CN_D296	CN_D292	CN_D288	CN_D252
5	IVNS1ABP	CN_D294	CN_D292	CN_D288	CN_D252	CN_D224
5	ITGBL1	CN_D318	CN_D302.2	CN_D298	CN_D296	CN_D251
5	ILDR2	CN_D310	CN_D302.2	CN_D253.2	CN_D252	CN_D224
5	ID4	CN_D296	CN_D292	CN_D288	CN_D252	CN_328

5	HSPA1L	CN_D292	CN_D288	CN_D253.2	CN_D252	CN_328
5	HSPA1B	CN_D292	CN_D288	CN_D253.2	CN_D252	CN_328
5	HSPA1A	CN_D292	CN_D288	CN_D253.2	CN_D252	CN_328
5	HSF1	CN_328	CN_D310	CN_D254	CN_D224	CN_330
5	HSD17B7	CN_D292	CN_D288	CN_D252	CN_D251	CN_D224
5	HRSP12	CN_D318	CN_D254	CN_D224	CN_330	CN_328
5	HORMAD1	CN_D292	CN_D288	CN_D251	CN_D227	CN_D224
5	HLA-L	CN_D292	CN_D288	CN_D253.2	CN_D252	CN_328
5	HLA-J	CN_D292	CN_D288	CN_D253.2	CN_D252	CN_328
5	HLA-F	CN_D292	CN_D288	CN_D253.2	CN_D252	CN_328
5	HLA-DRB6	CN_D296	CN_D292	CN_D288	CN_D252	CN_328
5	HLA-DQB1	CN_D296	CN_D292	CN_D288	CN_D252	CN_328
5	HLA-A	CN_D292	CN_D288	CN_D253.2	CN_D252	CN_328
5	HIST3H3	CN_D310	CN_D292	CN_D288	CN_D252	CN_320
5	HIST3H2BB	CN_D310	CN_D292	CN_D288	CN_D252	CN_320
5	HIST3H2A	CN_D310	CN_D292	CN_D288	CN_D252	CN_320
5	HIST2H2BE	CN_D300	CN_D288	CN_D227	CN_D224	CN_328
5	HIST2H2BC	CN_D300	CN_D288	CN_D227	CN_D224	CN_328
5	HIST2H2AC	CN_D300	CN_D288	CN_D227	CN_D224	CN_328
5	HIST2H2AB	CN_D300	CN_D288	CN_D227	CN_D224	CN_328
5	HIST1H4I	CN_D294	CN_D292	CN_D288	CN_D253.2	CN_328
5	HIST1H2BK	CN_D294	CN_D292	CN_D288	CN_D253.2	CN_328
5	HIST1H2BJ	CN_D294	CN_D292	CN_D288	CN_D253.2	CN_328
5	HIST1H2AH	CN_D294	CN_D292	CN_D288	CN_D253.2	CN_328
5	HIST1H2AG	CN_D294	CN_D292	CN_D288	CN_D253.2	CN_328
5	HEY1	CN_328	CN_D318	CN_D254	CN_D224	CN_330
5	HCN1	CN_D224	CN_D296	CN_D288	CN_D252	CN_D225
5	HCG9	CN_D292	CN_D288	CN_D253.2	CN_D252	CN_328
5	HCG27	CN_D292	CN_D288	CN_D253.2	CN_D252	CN_328
5	HCG18	CN_D292	CN_D288	CN_D253.2	CN_D252	CN_328
5	HAMP	CN_D316	CN_D310	CN_D298	CN_D294	CN_D251
5	GUSBP1	CN_D224	CN_D298	CN_D294	CN_D288	CN_D225
5	GTF2H4	CN_D292	CN_D288	CN_D253.2	CN_D252	CN_328
5	GSTA7P	CN_D296	CN_D292	CN_D288	CN_D252	CN_328
5	GSTA2	CN_D296	CN_D292	CN_D288	CN_D252	CN_328
5	GSDMD	CN_328	CN_D318	CN_D254	CN_D224	CN_330
5	GRM4	CN_D292	CN_D288	CN_D253.2	CN_D252	CN_328
5	GREM2	CN_D310	CN_D294	CN_D292	CN_D288	CN_D252
5	GPX5	CN_D292	CN_D288	CN_D253.2	CN_D252	CN_328
5	GPSM3	CN_D292	CN_D288	CN_D252	CN_D224	CN_328
5	GPR172A	CN_328	CN_D310	CN_D254	CN_D224	CN_330
5	GPLD1	CN_D296	CN_D292	CN_D288	CN_D252	CN_328
5	GPC6	CN_D302.2	CN_D296	CN_D253.2	CN_D251	CN_324

5	GPC5	CN_D318	CN_D302.2	CN_D253.2	CN_D251	CN_D227
5	GPANK1	CN_D296	CN_D292	CN_D288	CN_D252	CN_328
5	GORAB	CN_D294	CN_D292	CN_D288	CN_D252	CN_D224
5	GOLPH3L	CN_D292	CN_D288	CN_D251	CN_D227	CN_D224
5	GOLPH3	CN_D224	ZS.DNA12	CN_D296	CN_D288	CN_D225
5	GMDS	CN_D292	CN_D288	CN_D253.2	CN_D252	CN_328
5	GLYATL3	CN_D296	CN_D292	CN_D288	CN_D252	CN_328
5	GLP1R	CN_D296	CN_D292	CN_D288	CN_D253.2	CN_D252
5	GLI4	CN_328	CN_D318	CN_D254	CN_D224	CN_330
5	GDAP1	CN_328	CN_D318	CN_D254	CN_D224	CN_330
5	GCNT2	CN_D292	CN_D288	CN_D253.2	CN_D252	CN_328
5	GAS5	CN_D302.2	CN_D300	CN_D292	CN_D252	CN_D224
5	GABBR1	CN_D292	CN_D288	CN_D252	CN_D224	CN_328
5	FOXP4	CN_D296	CN_D292	CN_D288	CN_D252	CN_328
5	FGF10	CN_D224	CN_D288	CN_D253.2	CN_D252	CN_D225
5	FGD2	CN_D296	CN_D292	CN_D288	CN_D252	CN_328
5	FCRL5	CN_D310	CN_D294	CN_D252	CN_D251	CN_D224
5	FCGR3B	CN_D294	CN_D292	CN_D252	CN_D251	CN_D224
5	FCGR3A	CN_D300	CN_D294	CN_D252	CN_D251	CN_D224
5	FBXO32	CN_D224	CN_328	CN_D296	CN_D254	CN_330
5	FBXO28	CN_D296	CN_D292	CN_D288	CN_D252	CN_320
5	FBXL6	CN_328	CN_D310	CN_D254	CN_D224	CN_330
5	FARS2	CN_D316	CN_D292	CN_D288	CN_D253.2	CN_D252
5	FAM91A1	CN_D224	CN_328	CN_D318	CN_D254	CN_330
5	FAM84B	CN_D224	CN_D288	CN_D254	CN_330	CN_328
5	FAM83H	CN_328	CN_D318	CN_D254	CN_D224	CN_330
5	FAM83A	CN_328	CN_D288	CN_D254	CN_D224	CN_330
5	FAM78B	CN_D310	CN_D292	CN_D288	CN_D252	CN_D224
5	FAM5B	CN_D300	CN_D292	CN_D288	CN_D252	CN_D224
5	FAM50B	CN_D296	CN_D292	CN_D288	CN_D252	CN_328
5	FAM177B	CN_D300	CN_D294	CN_D292	CN_D288	CN_D252
5	FAM150A	CN_328	CN_D318	CN_D310	CN_D288	CN_D224
5	FABP9	CN_328	CN_D318	CN_D254	CN_D224	CN_330
5	FABP5	CN_328	CN_D318	CN_D254	CN_D224	CN_330
5	FABP4	CN_328	CN_D318	CN_D254	CN_D224	CN_330
5	FABP12	CN_328	CN_D318	CN_D254	CN_D224	CN_330
5	EYA4	CN_D298	CN_D296	CN_D288	CN_D253.2	CN_328
5	ESRP1	CN_D316	CN_D254	CN_D224	CN_330	CN_328
5	ESR1	CN_D298	CN_D296	CN_D288	CN_D253.2	CN_D251
5	ERO1LB	CN_D300	CN_D292	CN_D288	CN_D252	CN_320
5	EPHX1	CN_D302.2	CN_D296	CN_D292	CN_D288	CN_D252
5	ENPP4	CN_D292	CN_D288	CN_D253.2	CN_D252	CN_328
5	EHMT2	CN_D292	CN_D288	CN_D253.2	CN_D252	CN_328

5	EFHA2	ZS.DNA26	CN_D302.2	CN_D298	CN_D296	CN_328
5	EEF1E1-MUTEI	CN_D292	CN_D288	CN_D252	CN_D224	CN_328
5	EEF1D	CN_328	CN_D318	CN_D254	CN_D224	CN_330
5	E2F5	CN_D224	CN_D318	CN_D254	CN_330	CN_328
5	E2F3	CN_D296	CN_D292	CN_D288	CN_D252	CN_328
5	DUSP5P	CN_D296	CN_D292	CN_D288	CN_D252	CN_320
5	DSP	CN_D292	CN_D288	CN_D253.2	CN_D252	CN_328
5	DPCR1	CN_D292	CN_D288	CN_D252	CN_D224	CN_328
5	DOM3Z	CN_D292	CN_D288	CN_D253.2	CN_D252	CN_328
5	DNAJC5B	CN_328	CN_D318	CN_D254	CN_D224	CN_330
5	DNAH8	CN_D296	CN_D292	CN_D288	CN_D253.2	CN_D252
5	DNAH5	CN_D298	CN_D296	CN_D288	CN_D252	CN_D225
5	DKFZP686I1521	CN_D296	CN_D292	CN_D288	CN_D252	CN_328
5	DKFZP564C196	CN_D302.2	CN_D300	CN_D292	CN_D252	CN_D224
5	DISP1	CN_D296	CN_D294	CN_D292	CN_D288	CN_D252
5	DISC2	CN_D310	CN_D294	CN_D292	CN_D288	CN_D252
5	DHX9	CN_D300	CN_D292	CN_D288	CN_D252	CN_D224
5	DHX16	CN_D296	CN_D292	CN_D288	CN_D252	CN_328
5	DGAT1	CN_328	CN_D310	CN_D254	CN_D224	CN_330
5	DERL1	CN_328	CN_D318	CN_D254	CN_D224	CN_330
5	DENND3	CN_328	CN_D310	CN_D254	CN_D224	CN_330
5	DEDD	CN_D310	CN_D300	CN_D292	CN_D252	CN_D251
5	DDR2	CN_D292	CN_D288	CN_D252	CN_D251	CN_D224
5	DDR1	CN_D292	CN_D288	CN_D253.2	CN_D252	CN_328
5	DAXX	CN_D292	CN_D288	CN_D253.2	CN_D252	CN_328
5	DAAM2	CN_D292	CN_D288	CN_D253.2	CN_D252	CN_328
5	CYP21A2	CN_D292	CN_D288	CN_D253.2	CN_D252	CN_328
5	CYP21A1P	CN_D292	CN_D288	CN_D253.2	CN_D252	CN_328
5	CTSS	CN_D292	CN_D288	CN_D251	CN_D227	CN_D224
5	CSNK2B	CN_D296	CN_D292	CN_D288	CN_D252	CN_328
5	CRISPLD1	CN_D318	CN_D310	CN_D254	CN_D224	CN_330
5	CRH	CN_328	CN_D318	CN_D254	CN_D224	CN_330
5	CRABP2	CN_D294	CN_D292	CN_D288	CN_D252	CN_D224
5	CR2	CN_D310	CN_D300	CN_D294	CN_D292	CN_D252
5	CR1	CN_D310	CN_D300	CN_D294	CN_D292	CN_D252
5	CNTNAP2	CN_D225	CN_D292	CN_D253.2	CN_D225	CN_D224
5	CNIH4	CN_D310	CN_D292	CN_D288	CN_D252	CN_320
5	CHRNB2	CN_D300	CN_D294	CN_D251	CN_D227	CN_328
5	CHRAC1	CN_D224	CN_328	CN_D254	CN_D251	CN_330
5	CHMP4C	CN_328	CN_D318	CN_D254	CN_D224	CN_330
5	CFB	CN_D292	CN_D288	CN_D253.2	CN_D252	CN_328
5	CENPQ	CN_D296	CN_D292	CN_D288	CN_D252	CN_328
5	CDK18	CN_D300	CN_D294	CN_D288	CN_D252	CN_320

5	CDH4	CN_D318	CN_D298	CN_D253.2	CN_D251	CN_D224
5	CDH18	CN_D224	CN_D296	CN_D288	CN_D252	CN_D225
5	CDH10	CN_D224	CN_D298	CN_D296	CN_D288	CN_D225
5	CD22	CN_D316	CN_D310	CN_D298	CN_D294	CN_D251
5	CCHCR1	CN_D292	CN_D288	CN_D253.2	CN_D252	CN_328
5	CACNA1C	CN_D318	CN_D298	CN_D296	CN_D254	CN_324
5	CA13	CN_D224	CN_D318	CN_D254	CN_330	CN_328
5	C8ORFK29	CN_328	CN_D310	CN_D254	CN_D224	CN_330
5	C8orf76	CN_328	CN_D288	CN_D254	CN_D224	CN_330
5	C8orf73	CN_328	CN_D318	CN_D254	CN_D224	CN_330
5	C8orf69	CN_D318	CN_D254	CN_D224	CN_330	CN_328
5	C8orf59	CN_D224	CN_D318	CN_D254	CN_330	CN_328
5	C8orf55	CN_328	CN_D318	CN_D254	CN_D224	CN_330
5	C8orf51	CN_328	CN_D318	CN_D254	CN_D224	CN_330
5	C8orf47	CN_D318	CN_D254	CN_D224	CN_330	CN_328
5	C8orf44-SGK3	CN_328	CN_D318	CN_D254	CN_D224	CN_330
5	C8orf44	CN_328	CN_D318	CN_D254	CN_D224	CN_330
5	C6orf89	CN_D296	CN_D292	CN_D288	CN_D252	CN_328
5	C6orf48	CN_D292	CN_D288	CN_D253.2	CN_D252	CN_328
5	C6orf47	CN_D296	CN_D292	CN_D288	CN_D252	CN_328
5	C6orf223	CN_D296	CN_D292	CN_D288	CN_D253.2	CN_328
5	C6orf141	CN_D296	CN_D292	CN_D288	CN_D252	CN_328
5	C6orf136	CN_D296	CN_D292	CN_D288	CN_D252	CN_328
5	C5orf42	CN_D224	CN_D296	CN_D288	CN_D252	CN_D225
5	C4B	CN_D292	CN_D288	CN_D253.2	CN_D252	CN_328
5	C4A	CN_D292	CN_D288	CN_D253.2	CN_D252	CN_328
5	C2	CN_D292	CN_D288	CN_D253.2	CN_D252	CN_328
5	C1orf96	CN_D300	CN_D292	CN_D288	CN_D252	CN_328
5	C1orf55	CN_D300	CN_D294	CN_D292	CN_D252	CN_328
5	C1orf53	CN_D296	CN_D294	CN_D292	CN_D252	CN_D224
5	C1orf198	CN_D310	CN_D300	CN_D292	CN_D288	CN_D252
5	C1orf182	CN_D300	CN_D292	CN_D252	CN_D251	CN_D224
5	C1orf114	CN_D310	CN_D292	CN_D252	CN_D224	CN_320
5	C1orf100	CN_D310	CN_D300	CN_D292	CN_D252	CN_320
5	BTBD9	CN_D292	CN_D288	CN_D253.2	CN_D252	CN_328
5	BOP1	CN_328	CN_D310	CN_D254	CN_D224	CN_330
5	BOLA1	CN_D300	CN_D288	CN_D227	CN_D224	CN_328
5	BMP6	CN_D294	CN_D292	CN_D288	CN_D252	CN_328
5	BLZF1	CN_D310	CN_D292	CN_D252	CN_D224	CN_320
5	BCAN	CN_D294	CN_D292	CN_D288	CN_D252	CN_D224
5	BAI1	CN_328	CN_D318	CN_D254	CN_D224	CN_330
5	BAG6	CN_D296	CN_D292	CN_D288	CN_D252	CN_328
5	B3GALT4	CN_D292	CN_D288	CN_D253.2	CN_D252	CN_328

5	AZIN1	CN_328	CN_D318	CN_D254	CN_D224	CN_330
5	AXDND1	CN_D310	CN_D296	CN_D292	CN_D252	CN_D224
5	ATXN1	CN_D292	CN_D288	CN_D253.2	CN_D252	CN_328
5	ATP6V1H	CN_D224	CN_328	CN_D318	CN_D310	CN_D288
5	ATAT1	CN_D296	CN_D292	CN_D288	CN_D252	CN_328
5	ATAD2	CN_328	CN_D288	CN_D254	CN_D224	CN_330
5	ARV1	CN_D310	CN_D302.2	CN_D292	CN_D288	CN_D252
5	APOM	CN_D296	CN_D292	CN_D288	CN_D252	CN_328
5	ANKRD46	CN_328	CN_D318	CN_D254	CN_D224	CN_330
5	ANGEL2	CN_D298	CN_D294	CN_D292	CN_D288	CN_D252
5	AIM2	CN_D300	CN_D294	CN_D252	CN_D251	CN_D224
5	AIF1	CN_D296	CN_D292	CN_D288	CN_D252	CN_328
5	ADCK5	CN_328	CN_D310	CN_D254	CN_D224	CN_330
5	ADAR	CN_D300	CN_D294	CN_D251	CN_D227	CN_328
5	ADAMTSL4	CN_D292	CN_D288	CN_D227	CN_D224	CN_320
5	ABR	ZS.DNA26	ZS.DNA11	CN_D306	CN_328	CN_324
5	ABL2	CN_D300	CN_D296	CN_D292	CN_D252	CN_D224
5	ABHD16A	CN_D296	CN_D292	CN_D288	CN_D252	CN_328
5	ABCF1	CN_D296	CN_D292	CN_D288	CN_D252	CN_328
5	AARS2	CN_D292	CN_D288	CN_D253.2	CN_D252	CN_328
4	ZSCAN23	CN_D292	CN_D288	CN_D252	CN_328	
4	ZSCAN16	CN_D292	CN_D288	CN_D252	CN_328	
4	ZSCAN12P1	CN_D292	CN_D288	CN_D252	CN_328	
4	ZSCAN12	CN_D292	CN_D288	CN_D252	CN_328	
4	ZNF850	CN_D316	CN_D310	CN_D298	CN_D294	
4	ZNF792	CN_D316	CN_D310	CN_D298	CN_D251	
4	ZNF76	CN_D292	CN_D288	CN_D252	CN_328	
4	ZNF767	CN_D300	CN_D292	CN_D253.2	CN_D225	
4	ZNF746	CN_D300	CN_D292	CN_D253.2	CN_D225	
4	ZNF707	CN_328	CN_D318	CN_D254	CN_D224	
4	ZNF687	CN_D310	CN_D227	CN_D224	CN_320	
4	ZNF599	CN_D316	CN_D298	CN_D294	CN_D251	
4	ZNF567	CN_D316	CN_D310	CN_D298	CN_D294	
4	ZNF467	CN_D300	CN_D292	CN_D253.2	CN_D225	
4	ZNF461	CN_D316	CN_D310	CN_D298	CN_D294	
4	ZNF407	CN_D294	CN_D292	CN_D253.2	CN_D251	
4	ZNF323	CN_D292	CN_D288	CN_D252	CN_328	
4	ZNF322	CN_D292	CN_D288	CN_D252	CN_328	
4	ZNF318	CN_D292	CN_D288	CN_D252	CN_328	
4	ZNF30	CN_D316	CN_D310	CN_D298	CN_D251	
4	ZNF302	CN_D316	CN_D298	CN_D294	CN_D251	
4	ZNF252	CN_D288	CN_D254	CN_D224	CN_330	
4	ZNF193	CN_D292	CN_D288	CN_D252	CN_328	

4	ZNF192	CN_D292	CN_D288	CN_D252	CN_328
4	ZNF181	CN_D316	CN_D298	CN_D294	CN_D251
4	ZNF165	CN_D292	CN_D288	CN_D252	CN_328
4	ZKSCAN3	CN_D292	CN_D288	CN_D252	CN_328
4	ZFP82	CN_D316	CN_D310	CN_D298	CN_D251
4	ZFP57	CN_D292	CN_D288	CN_D252	CN_328
4	ZFP36	CN_D316	CN_D310	CN_D298	CN_D251
4	ZFHX3	ZS.DNA9	CN_D318	CN_D306	CN_324
4	ZFAND3	CN_D292	CN_D288	CN_D252	CN_328
4	ZCCHC17	CN_D300	CN_D296	CN_D292	CN_D253.2
4	ZBTB9	CN_D292	CN_D288	CN_D252	CN_328
4	ZBTB10	CN_328	CN_D254	CN_D224	CN_330
4	ZADH2	ZS.DNA24	CN_D298	CN_D292	CN_D251
4	YWHAE	CN_D316	CN_D306	CN_328	CN_324
4	YOD1	CN_D294	CN_D288	CN_D253.2	CN_D252
4	YIPF3	CN_D292	CN_D288	CN_D252	CN_328
4	XRCC6BP1	CN_D310	CN_D298	CN_D296	CN_D254
4	XPO5	CN_D292	CN_D288	CN_D252	CN_328
4	WHSC1L1	CN_D318	CN_D300	CN_D253.2	CN_320
4	WDYHV1	CN_D224	CN_328	CN_D254	CN_330
4	WDR87	CN_D316	CN_D310	CN_D298	CN_D294
4	WDR62	CN_D310	CN_D294	CN_D253.2	CN_D251
4	WDR26	CN_D294	CN_D292	CN_D288	CN_D252
4	VPS45	CN_D292	CN_D288	CN_D227	CN_D224
4	VHLL	CN_D292	CN_D252	CN_D251	CN_D224
4	VEGFA	CN_D292	CN_D288	CN_D252	CN_328
4	VANGL2	CN_D294	CN_D288	CN_D252	CN_D224
4	URB2	CN_D310	CN_D300	CN_D292	CN_D252
4	UNC5CL	CN_D292	CN_D288	CN_D253.2	CN_D252
4	UNC45A	CN_D318	CN_D300	CN_D253.2	CN_324
4	UGT3A1	CN_D224	CN_D298	CN_D288	CN_D225
4	UGGT2	CN_D302.2	CN_D296	CN_D253.2	CN_D251
4	UBR2	CN_D292	CN_D288	CN_D253.2	CN_D252
4	UBE2W	CN_328	CN_D254	CN_D224	CN_330
4	UBE2Q2	CN_D318	CN_D300	CN_D253.2	CN_324
4	UBD	CN_D292	CN_D288	CN_D252	CN_328
4	UBAC2	CN_D318	CN_D302.2	CN_D296	CN_D251
4	TXNIP	CN_D310	CN_D296	CN_D227	CN_D224
4	TXNDC5	CN_D292	CN_D288	CN_D252	CN_328
4	TULP1	CN_D292	CN_D288	CN_D252	CN_328
4	TUBB	CN_D292	CN_D288	CN_D252	CN_328
4	TUBB2A	CN_D292	CN_D288	CN_D252	CN_328
4	TTC33	CN_D224	CN_D296	CN_D288	CN_D225

4	TTC23L	CN_D224	CN_D288	CN_D252	CN_D225
4	TTC13	CN_D310	CN_D292	CN_D288	CN_D252
4	TSPO2	CN_D292	CN_D288	CN_D253.2	CN_D252
4	TSPAN31	CN_D310	CN_D298	CN_D296	CN_D254
4	TSFM	CN_D310	CN_D298	CN_D296	CN_D254
4	TSC1	ZS.DNA28	CN_D296	CN_328	CN_324
4	TRIM46	CN_D300	CN_D294	CN_D251	CN_328
4	TRIM38	CN_D292	CN_D288	CN_D252	CN_328
4	TRERF1	CN_D292	CN_D253.2	CN_D252	CN_328
4	TPTE2P3	CN_D302.2	CN_D296	CN_D294	CN_D253.2
4	TPMT	CN_D296	CN_D292	CN_D252	CN_328
4	TP53BP2	CN_D294	CN_D292	CN_D288	CN_D252
4	TOR3A	CN_D296	CN_D292	CN_D252	CN_D224
4	TOR1AIP1	CN_D310	CN_D292	CN_D252	CN_D224
4	TOB2P1	CN_D292	CN_D288	CN_D252	CN_328
4	TMEM81	CN_D310	CN_D292	CN_D252	CN_320
4	TMEM79	CN_D292	CN_D252	CN_D251	CN_D224
4	TMEM206	CN_D310	CN_D292	CN_D288	CN_D252
4	TMEM14C	CN_D292	CN_D288	CN_D252	CN_328
4	TMEM14B	CN_D292	CN_D288	CN_D252	CN_328
4	TLL2	CN_D294	CN_D288	CN_D253.2	CN_330
4	TJAP1	CN_D292	CN_D288	CN_D252	CN_328
4	TIMM50	CN_D316	CN_D310	CN_D298	CN_D251
4	TIMM17A	CN_D300	CN_D292	CN_D252	CN_D224
4	THSD7A	CN_D225	CN_D298	CN_D253.2	CN_D224
4	THEM4	CN_D302.2	CN_D300	CN_D253.2	CN_320
4	THBS3	CN_D300	CN_D294	CN_D251	CN_328
4	TFAP2A	CN_D292	CN_D288	CN_D252	CN_328
4	TEX30	CN_D318	CN_D302.2	CN_D296	CN_D251
4	TERT	CN_D225	CN_D302.2	CN_D296	CN_328
4	TEAD3	CN_D292	CN_D288	CN_D252	CN_328
4	TDRG1	CN_D296	CN_D292	CN_D288	CN_D252
4	TCOF1	CN_D298	CN_D254	CN_D253.2	CN_328
4	TCF24	CN_328	CN_D254	CN_D224	CN_330
4	TCF12	CN_D318	CN_D300	CN_D253.2	CN_324
4	TCEA1	CN_D224	CN_328	CN_D318	CN_D310
4	TBCB	CN_D310	CN_D294	CN_D253.2	CN_D251
4	TBC1D2B	CN_D318	CN_D300	CN_D253.2	CN_324
4	TARBP1	CN_D294	CN_D292	CN_D252	CN_320
4	TAGAP	CN_D298	CN_D296	CN_D288	CN_D251
4	TAF8	CN_D292	CN_D288	CN_D252	CN_328
4	TADA1	CN_D310	CN_D253.2	CN_D252	CN_D224
4	SYNGAP1	CN_D292	CN_D288	CN_D252	CN_328

4	SYNE1	CN_D298	CN_D296	CN_D288	CN_D253.2
4	SV2B	CN_D318	CN_D318	CN_D300	CN_D253.2
4	SUSD4	CN_D310	CN_D300	CN_D292	CN_D252
4	SUPT5H	CN_D316	CN_D310	CN_D298	CN_D251
4	SUB1	CN_D224	CN_D296	CN_D288	CN_D225
4	STX7	CN_D298	CN_D296	CN_D288	CN_D253.2
4	ST6GALNAC3	CN_D300	CN_D296	CN_D253.2	CN_D224
4	SSPO	CN_D300	CN_D292	CN_D253.2	CN_D225
4	SRP9	CN_D302.2	CN_D292	CN_D288	CN_D252
4	SPHAR	CN_D300	CN_D292	CN_D288	CN_D252
4	SPEF2	CN_D224	CN_D296	CN_D288	CN_D225
4	SPATC1	CN_328	CN_D254	CN_D224	CN_330
4	SNTG1	CN_D318	CN_D288	CN_D251	CN_328
4	SNORD87	CN_328	CN_D254	CN_D224	CN_330
4	SNORD72	CN_D224	CN_D296	CN_D288	CN_D225
4	SNORA16B	CN_D310	CN_D292	CN_D288	CN_D252
4	SNHG6	CN_328	CN_D254	CN_D224	CN_330
4	SNAPIN	CN_D310	CN_D296	CN_D227	CN_328
4	SNAI2	CN_328	CN_D318	CN_D288	CN_D254
4	SMOC2	CN_D296	CN_D292	CN_D288	CN_D253.2
4	SMG5	CN_D292	CN_D252	CN_D251	CN_D224
4	SLC6A19	CN_D225	CN_D302.2	CN_D296	CN_328
4	SLC6A18	CN_D225	CN_D302.2	CN_D296	CN_328
4	SLC45A2	CN_D224	CN_D288	CN_D253.2	CN_D225
4	SLC41A1	CN_D294	CN_D292	CN_D288	CN_D252
4	SLC39A6	CN_D318	CN_D294	CN_D253.2	CN_D251
4	SLC35F1	CN_D298	CN_D296	CN_D288	CN_D253.2
4	SLC35B2	CN_D292	CN_D288	CN_D252	CN_328
4	SLC30A10	CN_D310	CN_D292	CN_D253.2	CN_D252
4	SLC29A1	CN_D292	CN_D288	CN_D252	CN_328
4	SLC26A10	CN_D310	CN_D298	CN_D296	CN_D254
4	SLC25A44	CN_D292	CN_D252	CN_D251	CN_D224
4	SLC24A2	CN_D302.2	CN_D296	CN_D251	CN_324
4	SLC22A5	CN_D298	CN_D254	CN_D253.2	CN_328
4	SLC17A4	CN_D292	CN_D288	CN_D252	CN_328
4	SLC17A3	CN_D292	CN_D288	CN_D252	CN_328
4	SLC17A2	CN_D292	CN_D288	CN_D252	CN_328
4	SLC17A1	CN_D292	CN_D288	CN_D252	CN_328
4	SLC14A2	CN_D294	CN_D292	CN_D253.2	CN_D251
4	SLC10A2	CN_D318	CN_D302.2	CN_D296	CN_D251
4	SIPA1L3	CN_D316	CN_D310	CN_D298	CN_D294
4	SIL1	ZS.DNA11	CN_D252	CN_D224	CN_324
4	SIKE1	CN_D300	CN_D296	CN_D253.2	CN_D224

4	SHISA4	CN_D300	CN_D292	CN_D252	CN_D224
4	SHC1	CN_D253.2	CN_D251	CN_D224	CN_328
4	SHARPIN	CN_328	CN_D254	CN_D224	CN_330
4	SGCZ	CN_D304	CN_D300	CN_D298	CN_D296
4	SERPINC1	CN_D300	CN_D292	CN_D252	CN_D224
4	SERINC2	CN_D300	CN_D296	CN_D292	CN_D253.2
4	SEMA6A	CN_D298	CN_D254	CN_D253.2	CN_D224
4	SELV	CN_D316	CN_D310	CN_D298	CN_D251
4	SELL	CN_D292	CN_D253.2	CN_D252	CN_D224
4	SDR42E1	CN_D296	CN_D251	CN_330	CN_320
4	SCYL3	CN_D302.2	CN_D292	CN_D288	CN_D252
4	SCUBE3	CN_D292	CN_D288	CN_D252	CN_328
4	SCRIB	CN_328	CN_D254	CN_D224	CN_330
4	SCN1B	CN_D316	CN_D310	CN_D298	CN_D251
4	SCGB2B3P	CN_D316	CN_D298	CN_D294	CN_D251
4	SCGB2B2	CN_D316	CN_D298	CN_D294	CN_D251
4	SCCPDH	CN_D302.2	CN_D296	CN_D292	CN_D252
4	SAYSD1	CN_D292	CN_D288	CN_D253.2	CN_D252
4	SACS	ZS.DNA25	CN_D318	CN_D302.2	CN_D253.2
4	S100A1	CN_D310	CN_D296	CN_D227	CN_328
4	S100A16	CN_D310	CN_D296	CN_D227	CN_328
4	S100A14	CN_D310	CN_D296	CN_D227	CN_328
4	S100A13	CN_D310	CN_D296	CN_D227	CN_328
4	RXFP3	CN_D224	CN_D288	CN_D253.2	CN_D225
4	RSPH9	CN_D292	CN_D288	CN_D252	CN_328
4	RPS21	CN_D318	CN_D298	CN_D296	CN_D251
4	RPS16P5	CN_D296	CN_D292	CN_D288	CN_D252
4	RPS16	CN_D316	CN_D310	CN_D298	CN_D251
4	RPS14	CN_D298	CN_D254	CN_D253.2	CN_328
4	RPS10-NUDT3	CN_D292	CN_D288	CN_D252	CN_328
4	RPS10	CN_D292	CN_D288	CN_D252	CN_328
4	RPP21	CN_D292	CN_D288	CN_D252	CN_328
4	RPL7L1	CN_D292	CN_D288	CN_D252	CN_328
4	RPL37	CN_D224	CN_D296	CN_D288	CN_D225
4	RPL10A	CN_D292	CN_D288	CN_D252	CN_328
4	RP1	CN_D224	CN_328	CN_D318	CN_D254
4	ROR1	CN_D300	CN_D296	CN_D288	CN_D253.2
4	RNPEP	CN_D300	CN_D292	CN_D252	CN_D224
4	RNF8	CN_D292	CN_D288	CN_D252	CN_328
4	RNF5	CN_D292	CN_D288	CN_D252	CN_328
4	RNF182	CN_D292	CN_D288	CN_D252	CN_328
4	RIPK1	CN_D292	CN_D288	CN_D252	CN_328
4	RGS5	CN_D292	CN_D252	CN_D251	CN_D224

4	RGS4	CN_D294	CN_D292	CN_D252	CN_D224
4	RCSD1	CN_D302.2	CN_D292	CN_D252	CN_D224
4	RCCD1	CN_D318	CN_D300	CN_D253.2	CN_324
4	RC3H1	CN_D292	CN_D288	CN_D252	CN_D224
4	RBMS1	CN_D318	CN_320	CN_D253.2	CN_D224
4	RBM38	CN_D318	CN_D296	CN_D253.2	CN_D251
4	RBBP5	CN_D310	CN_D292	CN_D252	CN_320
4	RB1CC1	CN_328	CN_D318	CN_D310	CN_D224
4	RASAL2	CN_D302.2	CN_D298	CN_D294	CN_D252
4	RAE1	CN_D318	CN_D296	CN_D253.2	CN_D251
4	RAD1	CN_D224	CN_D288	CN_D252	CN_D225
4	RAD17	CN_328	CN_D298	CN_330	CN_324
4	RAB7L1	CN_D294	CN_D292	CN_D288	CN_D252
4	RAB4A	CN_D300	CN_D292	CN_D288	CN_D252
4	PYGO2	CN_D253.2	CN_D251	CN_D224	CN_328
4	PXDNL	CN_328	CN_D318	CN_D288	CN_D224
4	PUF60	CN_328	CN_D254	CN_D224	CN_330
4	PTTG3P	CN_328	CN_D254	CN_D224	CN_330
4	PTPRT	CN_D318	CN_D253.2	CN_D251	CN_D224
4	PTPRR	CN_D310	CN_D298	CN_D254	CN_D253.2
4	PTPRN2	CN_D225	CN_D292	CN_D253.2	CN_D224
4	PTPRB	CN_D310	CN_D298	CN_D254	CN_D253.2
4	PTGER4	CN_D224	CN_D296	CN_D288	CN_D225
4	PSTPIP1	CN_D318	CN_D300	CN_D253.2	CN_324
4	PSORS1C2	CN_D292	CN_D288	CN_D252	CN_328
4	PSORS1C1	CN_D292	CN_D288	CN_D252	CN_328
4	PRUNE	CN_D294	CN_D251	CN_D227	CN_D224
4	PRSS16	CN_D292	CN_D288	CN_D253.2	CN_328
4	PRRT1	CN_D292	CN_D288	CN_D252	CN_328
4	PRR3	CN_D292	CN_D288	CN_D252	CN_328
4	PRPF4B	CN_D296	CN_D292	CN_D288	CN_328
4	PRPF3	CN_D300	CN_D292	CN_D227	CN_D224
4	PROX1	CN_D310	CN_D294	CN_D288	CN_D252
4	PRLR	CN_D224	CN_D288	CN_D253.2	CN_D225
4	PRL	CN_D296	CN_D292	CN_D288	CN_D252
4	PRKAA1	CN_D224	CN_D296	CN_D288	CN_D225
4	PRIM1	CN_D310	CN_D296	CN_D254	CN_D253.2
4	PRG4	CN_D294	CN_D292	CN_D252	CN_D224
4	PPT2-EGFL8	CN_D292	CN_D288	CN_D252	CN_328
4	PPT2	CN_D292	CN_D288	CN_D252	CN_328
4	PPP2R5A	CN_D310	CN_D292	CN_D288	CN_D252
4	PPP2R2B	CN_D298	CN_D254	CN_D253.2	CN_D224
4	PPP1R42	CN_328	CN_D254	CN_D224	CN_330

4	PPIP5K2	CN_D298	CN_D296	CN_D253.2	CN_D224
4	PPARD	CN_D292	CN_D288	CN_D252	CN_328
4	POU2F1	CN_D292	CN_D252	CN_D224	CN_320
4	POLR2I	CN_D310	CN_D294	CN_D253.2	CN_D251
4	POLR1C	CN_D292	CN_D288	CN_D252	CN_328
4	POLH	CN_D292	CN_D288	CN_D252	CN_328
4	PMVK	CN_D253.2	CN_D251	CN_D224	CN_328
4	PMF1	CN_D292	CN_D252	CN_D251	CN_D224
4	PMF1-BGLAP	CN_D292	CN_D252	CN_D251	CN_D224
4	PMCHL1	CN_D224	CN_D298	CN_D288	CN_D225
4	PM20D1	CN_D294	CN_D292	CN_D288	CN_D252
4	PLEKHG2	CN_D316	CN_D310	CN_D298	CN_D251
4	PLEC	CN_328	CN_D254	CN_D224	CN_330
4	PLCXD3	CN_D224	CN_D288	CN_D252	CN_D225
4	PLCG2	CN_D300	CN_D296	CN_D253.2	CN_D251
4	PKP1	CN_D294	CN_D292	CN_D252	CN_D224
4	PKHD1L1	CN_D310	CN_D254	CN_D224	CN_328
4	PIP4K2C	CN_D310	CN_D298	CN_D296	CN_D254
4	PIEZO2	CN_D318	CN_D294	CN_D251	CN_D224
4	PIAS3	CN_D292	CN_D253.2	CN_D227	CN_D224
4	PI4KB	CN_D310	CN_D227	CN_D224	CN_320
4	PHIP	CN_D296	CN_D292	CN_D288	CN_D253.2
4	PHF1	CN_D292	CN_D288	CN_D252	CN_328
4	PFKFB2	CN_D294	CN_D288	CN_D253.2	CN_D252
4	PEAK1	CN_D318	CN_D318	CN_D300	CN_D253.2
4	PEA15	CN_D310	CN_D292	CN_D251	CN_D224
4	PDZD2	CN_D224	CN_D288	CN_D252	CN_D225
4	PDLIM4	CN_D298	CN_D254	CN_D253.2	CN_328
4	PDGFRB	CN_D296	CN_D254	CN_D224	CN_328
4	PDE7B	CN_D298	CN_D296	CN_D288	CN_D253.2
4	PDE4D	CN_D298	CN_D296	CN_D224	CN_324
4	PCMTD1	CN_328	CN_D318	CN_D288	CN_D224
4	PCM1	CN_D300	CN_D296	CN_D253.2	CN_D251
4	PCDH9	CN_D302.2	CN_D298	CN_D296	CN_D253.2
4	PCCA	CN_D318	CN_D302.2	CN_D296	CN_D251
4	PBXIP1	CN_D253.2	CN_D251	CN_D224	CN_328
4	PBX2	CN_D292	CN_D288	CN_D252	CN_328
4	PARP10	CN_328	CN_D254	CN_D224	CN_330
4	PAQR6	CN_D292	CN_D252	CN_D251	CN_D224
4	PAPD7	CN_D296	CN_D288	CN_D253.2	CN_D225
4	PAM	CN_D298	CN_D296	CN_D253.2	CN_D224
4	PAK1IP1	CN_D292	CN_D288	CN_D252	CN_328
4	OSGIN1	CN_D227	CN_D225	CN_328	CN_324

4	OS9	CN_D310	CN_D298	CN_D296	CN_D254
4	OR5V1	CN_D292	CN_D288	CN_D252	CN_328
4	OR2J2	CN_D292	CN_D288	CN_D252	CN_328
4	OR2H1	CN_D292	CN_D288	CN_D252	CN_328
4	OR2B6	CN_D292	CN_D288	CN_D252	CN_328
4	OR2B2	CN_D292	CN_D288	CN_D252	CN_328
4	OR14J1	CN_D292	CN_D288	CN_D252	CN_328
4	OR12D3	CN_D292	CN_D288	CN_D252	CN_328
4	OR12D2	CN_D292	CN_D288	CN_D252	CN_328
4	OR11A1	CN_D292	CN_D288	CN_D252	CN_328
4	OR10J1	CN_D302.2	CN_D298	CN_D252	CN_D251
4	OR10C1	CN_D292	CN_D288	CN_D252	CN_328
4	OPRK1	CN_D224	CN_328	CN_D318	CN_D310
4	OPN5	CN_D292	CN_D288	CN_D253.2	CN_D252
4	OPLAH	CN_328	CN_D254	CN_D224	CN_330
4	OBSCN	CN_D294	CN_D292	CN_D288	CN_D252
4	NUP153	CN_D292	CN_D288	CN_D252	CN_328
4	NUP133	CN_D310	CN_D292	CN_D252	CN_328
4	NUF2	CN_D292	CN_D252	CN_D251	CN_D224
4	NUDT3	CN_D292	CN_D288	CN_D252	CN_328
4	NSF	CN_D306	CN_D251	CN_328	CN_324
4	NRG4	CN_D318	CN_D300	CN_D253.2	CN_324
4	NRBP2	CN_328	CN_D254	CN_D224	CN_330
4	NQO2	CN_D292	CN_D288	CN_D252	CN_328
4	NPR1	CN_D310	CN_D296	CN_D227	CN_328
4	NPM1	CN_D254	CN_D253.2	CN_328	CN_324
4	NPHS2	CN_D296	CN_D292	CN_D252	CN_D224
4	NPBWR1	CN_328	CN_D318	CN_D310	CN_D224
4	NOTCH2NL	CN_D310	CN_D288	CN_D251	CN_D224
4	NNT	CN_D224	CN_D296	CN_D252	CN_D225
4	NID1	CN_D294	CN_D292	CN_D288	CN_D252
4	NHLRC1	CN_D296	CN_D292	CN_D252	CN_328
4	NHLH1	CN_D294	CN_D288	CN_D252	CN_D224
4	NFYA	CN_D292	CN_D288	CN_D253.2	CN_D252
4	NFKBIE	CN_D292	CN_D288	CN_D252	CN_328
4	NFASC	CN_D310	CN_D292	CN_D252	CN_320
4	NETO1	CN_D296	CN_D294	CN_D292	CN_D251
4	NENF	CN_D310	CN_D292	CN_D288	CN_D252
4	NEK2	CN_D302.2	CN_D294	CN_D292	CN_D252
4	NEDD9	CN_D292	CN_D288	CN_D252	CN_328
4	NEDD4	CN_D318	CN_D300	CN_D253.2	CN_D252
4	NECAB2	CN_D227	CN_D225	CN_328	CN_324
4	NCR2	CN_D296	CN_D292	CN_D288	CN_D252

4	NBEA	CN_D302.2	CN_D298	CN_D252	CN_D251
4	NAV2	CN_D254	CN_D253.2	CN_D252	CN_D251
4	NAP1L1	CN_D310	CN_D298	CN_D296	CN_D254
4	NALCN	CN_D318	CN_D302.2	CN_D296	CN_D251
4	NAE1	CN_D298	CN_D251	CN_328	CN_320
4	NADKD1	CN_D224	CN_D288	CN_D252	CN_D225
4	NACA	CN_D310	CN_D296	CN_D254	CN_D253.2
4	MYO5A	CN_D318	CN_D300	CN_D253.2	CN_330
4	MYO1C	CN_D316	CN_D292	CN_328	CN_324
4	MYLIP	CN_D296	CN_D292	CN_D288	CN_D252
4	MUC1	CN_D300	CN_D294	CN_D251	CN_328
4	MSTO1	CN_D252	CN_D251	CN_D227	CN_D224
4	MRS2	CN_D296	CN_D292	CN_D288	CN_D252
4	MRPS30	CN_D224	CN_D288	CN_D252	CN_D225
4	MRPS21	CN_D300	CN_D292	CN_D227	CN_D224
4	MRPS18A	CN_D292	CN_D288	CN_D252	CN_328
4	MOXD1	CN_D298	CN_D296	CN_D288	CN_D253.2
4	MOS	CN_D224	CN_328	CN_D288	CN_330
4	MNDA	CN_D292	CN_D252	CN_D251	CN_D224
4	MLPH	CN_D318	CN_D292	CN_D253.2	CN_D224
4	MLLT4	CN_D298	CN_D296	CN_D288	CN_328
4	MLLT11	CN_D294	CN_D251	CN_D227	CN_D224
4	MLL3	CN_D292	CN_D253.2	CN_D225	CN_324
4	MIR937	CN_328	CN_D254	CN_D224	CN_330
4	MIR92B	CN_D300	CN_D294	CN_D251	CN_328
4	MIR661	CN_328	CN_D254	CN_D224	CN_330
4	MIR555	CN_D310	CN_D294	CN_D252	CN_D251
4	MIR548T	CN_D225	CN_D292	CN_D253.2	CN_D225
4	MIR548I4	CN_D225	CN_D292	CN_D253.2	CN_D224
4	MIR548AN	CN_D318	CN_D302.2	CN_D296	CN_D251
4	MIR548A1	CN_D316	CN_D292	CN_D288	CN_D252
4	MIR5095	CN_D318	CN_D296	CN_D253.2	CN_D251
4	MIR4758	CN_D318	CN_D298	CN_D296	CN_D251
4	MIR4748	ZS.DNA11	CN_D316	CN_328	CN_324
4	MIR4735	CN_D310	CN_D292	CN_D252	CN_D224
4	MIR4705	CN_D302.2	CN_D296	CN_D253.2	CN_D251
4	MIR4654	CN_D292	CN_D288	CN_D252	CN_D224
4	MIR4647	CN_D292	CN_D288	CN_D252	CN_328
4	MIR4639	CN_D296	CN_D292	CN_D288	CN_D252
4	MIR4530	CN_D316	CN_D310	CN_D298	CN_D251
4	MIR4462	CN_D292	CN_D288	CN_D252	CN_328
4	MIR4457	CN_D225	CN_D302.2	CN_D296	CN_328
4	MIR4424	CN_D310	CN_D292	CN_D252	CN_D224

4	MIR3936	CN_D298	CN_D254	CN_D253.2	CN_328
4	MIR3912	CN_D254	CN_D253.2	CN_328	CN_324
4	MIR3143	CN_D292	CN_D288	CN_D253.2	CN_328
4	MIR3134	CN_D306	CN_D296	CN_D253.2	CN_D224
4	MIR3121	CN_D300	CN_D292	CN_D252	CN_D224
4	MIR26A2	CN_D310	CN_D298	CN_D296	CN_D254
4	MIR2681	CN_D318	CN_D302.2	CN_D296	CN_D251
4	MIR2053	CN_D318	CN_D224	CN_330	CN_328
4	MIR181B1	CN_D294	CN_D292	CN_D253.2	CN_D252
4	MIR181A1	CN_D294	CN_D292	CN_D253.2	CN_D252
4	MIR1231	CN_D300	CN_D292	CN_D252	CN_D224
4	MIA3	CN_D294	CN_D292	CN_D288	CN_D252
4	MGC4473	CN_D294	CN_D292	CN_D252	CN_D224
4	METTL21CP1	CN_D318	CN_D302.2	CN_D296	CN_D251
4	METTL21C	CN_D318	CN_D302.2	CN_D296	CN_D251
4	METTL21B	CN_D310	CN_D298	CN_D296	CN_D254
4	METTL1	CN_D310	CN_D298	CN_D296	CN_D254
4	MEP1A	CN_D296	CN_D292	CN_D288	CN_D252
4	MED29	CN_D316	CN_D310	CN_D298	CN_D251
4	MED20	CN_D292	CN_D288	CN_D252	CN_328
4	MED10	CN_D296	CN_D288	CN_D253.2	CN_D225
4	MCM3	CN_D296	CN_D292	CN_D288	CN_D252
4	MAS1L	CN_D292	CN_D288	CN_D252	CN_328
4	MARVELD2	CN_328	CN_D298	CN_330	CN_324
4	MARCH9	CN_D310	CN_D298	CN_D296	CN_D254
4	MARCH6	CN_D288	CN_D253.2	CN_D225	CN_328
4	MAPT-IT1	CN_D306	CN_D296	CN_328	CN_324
4	MAN2A2	CN_D318	CN_D300	CN_D253.2	CN_324
4	MAML1	CN_D296	CN_D288	CN_328	CN_324
4	MAF1	CN_328	CN_D254	CN_D224	CN_330
4	MAD2L1BP	CN_D292	CN_D288	CN_D252	CN_328
4	MACROD2	CN_D298	CN_D296	CN_D253.2	CN_D224
4	LYPLA1	CN_D224	CN_328	CN_D318	CN_D310
4	LYN	CN_D224	CN_328	CN_D310	CN_330
4	LY96	CN_328	CN_D254	CN_D224	CN_330
4	LY86	CN_D292	CN_D288	CN_D252	CN_328
4	LRRC73	CN_D292	CN_D288	CN_D252	CN_328
4	LRRC52	CN_D310	CN_D294	CN_D252	CN_320
4	LRRC37A2	CN_D316	CN_D306	CN_328	CN_324
4	LRP1B	CN_D292	CN_D254	CN_D253.2	CN_D224
4	LPAR5	CN_D296	CN_D254	CN_D252	CN_324
4	LPAL2	CN_D296	CN_D288	CN_D253.2	CN_D251
4	LOC91450	CN_D318	CN_D300	CN_D253.2	CN_324

4	LOC730159	CN_D294	CN_D292	CN_D252	CN_D224
4	LOC729177	CN_D296	CN_D292	CN_D288	CN_D252
4	LOC727849	CN_D318	CN_D304	CN_D300	CN_D253.2
4	LOC653566	CN_D316	CN_D306	CN_D253.2	CN_324
4	LOC645166	CN_D306	CN_D292	CN_D252	CN_D227
4	LOC643401	CN_D224	CN_D298	CN_D296	CN_D225
4	LOC554223	CN_D292	CN_D288	CN_D252	CN_328
4	LOC440354	CN_D298	CN_328	CN_D252	CN_324
4	LOC440297	CN_D318	CN_D304	CN_D300	CN_D253.2
4	LOC401397	CN_D225	CN_D316	CN_D253.2	CN_D224
4	LOC400685	CN_D316	CN_D298	CN_D294	CN_D251
4	LOC390660	CN_D318	CN_D318	CN_D304	CN_D253.2
4	LOC340113	CN_D224	CN_D296	CN_D288	CN_D225
4	LOC340107	CN_D224	CN_D298	CN_D288	CN_D225
4	LOC339298	CN_D296	CN_D294	CN_D292	CN_D253.2
4	LOC286094	CN_D318	CN_D254	CN_D224	CN_330
4	LOC285768	CN_D292	CN_D288	CN_D252	CN_328
4	LOC221442	CN_D292	CN_D288	CN_D253.2	CN_D252
4	LOC153910	CN_D296	CN_D288	CN_D251	CN_328
4	LOC149134	CN_D302.2	CN_D296	CN_D292	CN_D252
4	LOC100652909	CN_D316	CN_D310	CN_D298	CN_D251
4	LOC100631378	CN_D316	CN_D310	CN_D298	CN_D294
4	LOC100508120	CN_D292	CN_D288	CN_D253.2	CN_D252
4	LOC100507632	CN_D224	CN_328	CN_D310	CN_D253.2
4	LOC100507547	CN_D292	CN_D288	CN_D252	CN_328
4	LOC100507362	CN_D292	CN_D288	CN_D252	CN_328
4	LOC100507194	CN_D292	CN_D288	CN_D252	CN_328
4	LOC100507173	CN_D292	CN_D288	CN_D224	CN_328
4	LOC100506844	CN_D310	CN_D298	CN_D296	CN_D254
4	LOC100506548	CN_D224	CN_D296	CN_D288	CN_D225
4	LOC100506207	CN_D296	CN_D292	CN_D288	CN_D252
4	LOC100506023	CN_D294	CN_D292	CN_D253.2	CN_D252
4	LOC100287718	CN_D296	CN_D292	CN_D288	CN_D252
4	LOC100270746	CN_D292	CN_D288	CN_D253.2	CN_328
4	LOC100132735	CN_D298	CN_D296	CN_D288	CN_D251
4	LOC100132356	CN_D224	CN_D298	CN_D296	CN_D225
4	LOC100131234	CN_D294	CN_D292	CN_D253.2	CN_D252
4	LOC100130776	CN_D310	CN_D298	CN_D296	CN_D254
4	LOC100130357	CN_D292	CN_D288	CN_D253.2	CN_D252
4	LOC100128993	CN_D298	CN_D253.2	CN_D251	CN_324
4	LOC100128675	CN_D316	CN_D310	CN_D298	CN_D251
4	LMOD1	CN_D300	CN_D292	CN_D252	CN_D224
4	LINC00518	CN_D292	CN_D288	CN_D252	CN_328

4	LINC00326	CN_D318	CN_D296	CN_D288	CN_D253.2
4	LINC00240	CN_D292	CN_D288	CN_D253.2	CN_328
4	LIN9	CN_D294	CN_D292	CN_D252	CN_328
4	LGR6	CN_D294	CN_D292	CN_D252	CN_D224
4	LGI4	CN_D316	CN_D310	CN_D298	CN_D251
4	LCE4A	CN_D292	CN_D251	CN_328	CN_320
4	LCE2A	CN_D292	CN_D251	CN_328	CN_320
4	LBR	CN_D294	CN_D292	CN_D252	CN_320
4	LAMA5	CN_D318	CN_D298	CN_D296	CN_D251
4	LAMA3	CN_D318	CN_D294	CN_D253.2	CN_D251
4	KRTCAP2	CN_D300	CN_D294	CN_D251	CN_328
4	KRT36	CN_D306	CN_D296	CN_D252	CN_D224
4	KRT35	CN_D306	CN_D296	CN_D252	CN_D224
4	KRT32	CN_D306	CN_D296	CN_D252	CN_D224
4	KRR1	CN_D310	CN_D298	CN_D296	CN_D254
4	KMO	CN_D300	CN_D292	CN_D253.2	CN_D252
4	KLHL1	CN_D302.2	CN_D296	CN_D253.2	CN_D251
4	KLHDC9	CN_D310	CN_D302.2	CN_D300	CN_D292
4	KIRREL	CN_D292	CN_D288	CN_D252	CN_D224
4	KIFAP3	CN_D300	CN_D292	CN_D288	CN_D252
4	KIF25	CN_D298	CN_D296	CN_D288	CN_328
4	KIF21B	CN_D310	CN_D292	CN_D252	CN_D224
4	KIAA1875	CN_328	CN_D254	CN_D224	CN_330
4	KIAA1715	CN_D318	CN_D306	CN_D253.2	CN_D224
4	KIAA1614	CN_D298	CN_D294	CN_D252	CN_D224
4	KIAA0319	CN_D292	CN_D288	CN_D252	CN_328
4	KIAA0240	CN_D292	CN_D288	CN_D252	CN_328
4	KDM4C	ZS.DNA24	CN_D296	CN_D224	CN_324
4	KDM1B	CN_D296	CN_D292	CN_D252	CN_328
4	KDELC1	CN_D318	CN_D302.2	CN_D296	CN_D251
4	KCTD3	CN_D294	CN_D292	CN_D288	CN_D252
4	KCNN3	CN_D253.2	CN_D251	CN_D224	CN_328
4	KCNC2	CN_D310	CN_D296	CN_D254	CN_D252
4	JRK	CN_328	CN_D318	CN_D254	CN_D224
4	ITGA10	CN_D310	CN_D253.2	CN_D227	CN_D224
4	ITFG1	CN_D304	CN_D296	CN_D253.2	CN_320
4	ISPD	CN_D298	CN_D253.2	CN_D225	CN_D224
4	IRX4	CN_D298	CN_D288	CN_D225	CN_328
4	IRF4	CN_D292	CN_D288	CN_D252	CN_328
4	IRF2BP2	CN_D300	CN_D294	CN_D292	CN_D252
4	IPO9	CN_D300	CN_D292	CN_D252	CN_D224
4	INPP5K	CN_D316	CN_D292	CN_328	CN_324
4	ING4	CN_D300	CN_D254	CN_D252	CN_324

4	ILF2	CN_D310	CN_D296	CN_D227	CN_328
4	IL7R	CN_D224	CN_D296	CN_D288	CN_D225
4	IL3	CN_D296	CN_D224	CN_328	CN_324
4	IL24	CN_D294	CN_D292	CN_D288	CN_D252
4	IL19	CN_D310	CN_D294	CN_D292	CN_D252
4	IL17F	CN_D296	CN_D292	CN_D288	CN_D252
4	IL10	CN_D310	CN_D294	CN_D292	CN_D252
4	IKBKAP	CN_D302.2	CN_D296	CN_D253.2	CN_D224
4	IFITM4P	CN_D292	CN_D288	CN_D252	CN_328
4	IFI16	CN_D252	CN_D251	CN_D224	CN_320
4	IER3	CN_D292	CN_D288	CN_D252	CN_328
4	ICK	CN_D296	CN_D292	CN_D288	CN_D252
4	IBA57	CN_D300	CN_D292	CN_D252	CN_320
4	HSP90AB1	CN_D292	CN_D288	CN_D252	CN_328
4	HS6ST3	CN_D302.2	CN_D296	CN_D253.2	CN_D251
4	HS3ST4	ZS.DNA24	CN_D294	CN_D253.2	CN_320
4	HPSE2	CN_D288	CN_D253.2	CN_D251	CN_330
4	HPN	CN_D316	CN_D310	CN_D298	CN_D251
4	HNRNPA1L2	CN_D302.2	CN_D296	CN_D294	CN_D253.2
4	HMGCLL1	CN_D296	CN_D292	CN_D288	CN_D253.2
4	HMGA2	CN_D310	CN_D298	CN_D296	CN_D254
4	HMGA1	CN_D292	CN_D288	CN_D252	CN_328
4	HLA-G	CN_D292	CN_D288	CN_D252	CN_328
4	HLA-F-AS1	CN_D292	CN_D288	CN_D252	CN_328
4	HLA-E	CN_D292	CN_D288	CN_D252	CN_328
4	HLA-DRB3	CN_D296	CN_D292	CN_D288	CN_328
4	HLA-DQA1	CN_D292	CN_D288	CN_D252	CN_328
4	HIST1H4L	CN_D292	CN_D288	CN_D252	CN_328
4	HIST1H4H	CN_D292	CN_D288	CN_D252	CN_328
4	HIST1H4G	CN_D292	CN_D288	CN_D252	CN_328
4	HIST1H4F	CN_D292	CN_D288	CN_D252	CN_328
4	HIST1H4E	CN_D292	CN_D288	CN_D252	CN_328
4	HIST1H4D	CN_D292	CN_D288	CN_D252	CN_328
4	HIST1H4C	CN_D292	CN_D288	CN_D252	CN_328
4	HIST1H4B	CN_D292	CN_D288	CN_D252	CN_328
4	HIST1H4A	CN_D292	CN_D288	CN_D252	CN_328
4	HIST1H3J	CN_D292	CN_D288	CN_D252	CN_328
4	HIST1H3I	CN_D292	CN_D288	CN_D252	CN_328
4	HIST1H3G	CN_D292	CN_D288	CN_D252	CN_328
4	HIST1H3F	CN_D292	CN_D288	CN_D252	CN_328
4	HIST1H3E	CN_D292	CN_D288	CN_D252	CN_328
4	HIST1H3D	CN_D292	CN_D288	CN_D252	CN_328
4	HIST1H3C	CN_D292	CN_D288	CN_D252	CN_328

4	HIST1H3B	CN_D292	CN_D288	CN_D252	CN_328
4	HIST1H3A	CN_D292	CN_D288	CN_D252	CN_328
4	HIST1H2BO	CN_D292	CN_D288	CN_D252	CN_328
4	HIST1H2BI	CN_D292	CN_D288	CN_D252	CN_328
4	HIST1H2BH	CN_D292	CN_D288	CN_D252	CN_328
4	HIST1H2BG	CN_D292	CN_D288	CN_D252	CN_328
4	HIST1H2BF	CN_D292	CN_D288	CN_D252	CN_328
4	HIST1H2BE	CN_D292	CN_D288	CN_D252	CN_328
4	HIST1H2BD	CN_D292	CN_D288	CN_D252	CN_328
4	HIST1H2BC	CN_D292	CN_D288	CN_D252	CN_328
4	HIST1H2BB	CN_D292	CN_D288	CN_D252	CN_328
4	HIST1H2BA	CN_D292	CN_D288	CN_D252	CN_328
4	HIST1H2APS1	CN_D292	CN_D288	CN_D252	CN_328
4	HIST1H2AM	CN_D292	CN_D288	CN_D252	CN_328
4	HIST1H2AE	CN_D292	CN_D288	CN_D252	CN_328
4	HIST1H2AD	CN_D292	CN_D288	CN_D252	CN_328
4	HIST1H2AC	CN_D292	CN_D288	CN_D252	CN_328
4	HIST1H2AB	CN_D292	CN_D288	CN_D252	CN_328
4	HIST1H2AA	CN_D292	CN_D288	CN_D252	CN_328
4	HIST1H1T	CN_D292	CN_D288	CN_D252	CN_328
4	HIST1H1E	CN_D292	CN_D288	CN_D252	CN_328
4	HIST1H1D	CN_D292	CN_D288	CN_D252	CN_328
4	HIST1H1C	CN_D292	CN_D288	CN_D252	CN_328
4	HIST1H1B	CN_D292	CN_D288	CN_D252	CN_328
4	HIST1H1A	CN_D292	CN_D288	CN_D252	CN_328
4	HHIPL2	CN_D292	CN_D288	CN_D252	CN_320
4	HGC6.3	CN_D298	CN_D296	CN_D288	CN_328
4	HFE	CN_D292	CN_D288	CN_D252	CN_328
4	HEATR7B2	CN_D224	CN_D296	CN_D288	CN_D225
4	HEATR7A	CN_328	CN_D254	CN_D224	CN_330
4	HDGFL1	CN_D296	CN_D292	CN_D288	CN_D252
4	HDDC3	CN_D318	CN_D300	CN_D253.2	CN_324
4	HCRTR2	CN_D296	CN_D292	CN_D288	CN_D253.2
4	HCG4	CN_D292	CN_D288	CN_D252	CN_328
4	HCG22	CN_D292	CN_D288	CN_D252	CN_328
4	HBS1L	CN_D298	CN_D296	CN_D288	CN_D253.2
4	HAS3	CN_D296	CN_328	CN_324	CN_320
4	HAS2	CN_D254	CN_D224	CN_330	CN_328
4	HAS2-AS1	CN_D254	CN_D224	CN_330	CN_328
4	H3F3AP4	CN_D294	CN_D292	CN_D252	CN_328
4	H3F3A	CN_D294	CN_D292	CN_D252	CN_328
4	GUSBP2	CN_D292	CN_D288	CN_D253.2	CN_328
4	GTPBP2	CN_D292	CN_D288	CN_D252	CN_328

4	GRINA	CN_328	CN_D254	CN_D224	CN_330
4	GRID2	CN_D302.2	CN_D298	CN_D251	CN_D224
4	GRAMD1A	CN_D316	CN_D310	CN_D298	CN_D251
4	GPX6	CN_D292	CN_D288	CN_D252	CN_328
4	GPR85	CN_D225	CN_D316	CN_D253.2	CN_D224
4	GPR126	CN_D296	CN_D288	CN_D251	CN_328
4	GPR116	CN_D296	CN_D292	CN_D288	CN_D252
4	GPR115	CN_D292	CN_D288	CN_D253.2	CN_D252
4	GPR111	CN_D292	CN_D288	CN_D253.2	CN_D252
4	GPAA1	CN_328	CN_D254	CN_D224	CN_330
4	GON4L	CN_D252	CN_D251	CN_D227	CN_D224
4	GNL1	CN_D292	CN_D288	CN_D252	CN_328
4	GNG4	CN_D310	CN_D294	CN_D292	CN_D252
4	GMPR	CN_D292	CN_D288	CN_D252	CN_328
4	GMNN	CN_D306	CN_D292	CN_D288	CN_D252
4	GLIS3	CN_D296	CN_D253.2	CN_D224	CN_324
4	GLIPR1L1	CN_D310	CN_D298	CN_D254	CN_D252
4	GIN1	CN_D298	CN_D296	CN_D253.2	CN_D224
4	GFRAL	CN_D296	CN_D292	CN_D288	CN_D253.2
4	GFOD1	CN_D292	CN_D288	CN_D252	CN_328
4	GCLC	CN_D296	CN_D292	CN_D288	CN_D252
4	GAN	CN_D296	CN_D253.2	CN_D251	CN_320
4	GALNTL6	CN_D304	CN_D302.2	CN_D288	CN_320
4	GALNTL4	CN_D251	CN_D298	CN_D254	CN_D253.2
4	GALNT11	CN_D292	CN_D253.2	CN_D225	CN_324
4	GABPB2	CN_D294	CN_D251	CN_D227	CN_D224
4	FXYD7	CN_D316	CN_D310	CN_D298	CN_D251
4	FXYD5	CN_D316	CN_D310	CN_D298	CN_D251
4	FXYD3	CN_D316	CN_D310	CN_D298	CN_D251
4	FXYD1	CN_D316	CN_D310	CN_D298	CN_D251
4	FURIN	CN_D318	CN_D300	CN_D253.2	CN_324
4	FTSJD2	CN_D292	CN_D288	CN_D252	CN_328
4	FSBP	CN_D318	CN_D254	CN_330	CN_328
4	FRMD1	CN_D298	CN_D296	CN_D288	CN_328
4	FREM1	CN_D296	CN_D292	CN_D253.2	CN_D224
4	FOXQ1	CN_D292	CN_D288	CN_D252	CN_328
4	FOXC1	CN_D292	CN_D288	CN_D252	CN_328
4	FMO9P	CN_D292	CN_D253.2	CN_D252	CN_D224
4	FLOT1	CN_D292	CN_D288	CN_D252	CN_328
4	FLJ41649	CN_D296	CN_D292	CN_D288	CN_D252
4	FLJ33360	CN_D298	CN_D288	CN_D253.2	CN_D225
4	FLJ30838	CN_D298	CN_D253.2	CN_D227	CN_D224
4	FKBPL	CN_D292	CN_D288	CN_D252	CN_328

4	FKBP5	CN_D296	CN_D292	CN_D252	CN_328
4	FHOD3	CN_D318	CN_D294	CN_D253.2	CN_D251
4	FGF18	CN_D254	CN_D253.2	CN_328	CN_324
4	FGF17	CN_D298	CN_D253.2	CN_D227	CN_324
4	FFAR3	CN_D316	CN_D310	CN_D298	CN_D251
4	FFAR1	CN_D316	CN_D310	CN_D298	CN_D251
4	FES	CN_D318	CN_D300	CN_D253.2	CN_324
4	FCRL4	CN_D294	CN_D252	CN_D251	CN_D224
4	FANCE	CN_D292	CN_D288	CN_D252	CN_328
4	FAM91A2	CN_D300	CN_D288	CN_D251	CN_320
4	FAM8A1	CN_D292	CN_D288	CN_D252	CN_328
4	FAM83B	CN_D296	CN_D292	CN_D288	CN_D253.2
4	FAM65B	CN_D306	CN_D292	CN_D288	CN_D252
4	FAM63A	CN_D294	CN_D251	CN_D227	CN_D224
4	FAM58BP	CN_D300	CN_D294	CN_D252	CN_320
4	FAM217A	CN_D296	CN_D292	CN_D288	CN_328
4	FAM214A	CN_D318	CN_D300	CN_D253.2	CN_324
4	FAM20B	CN_D296	CN_D292	CN_D252	CN_D224
4	FAM203A	CN_328	CN_D254	CN_D224	CN_330
4	FAM187B	CN_D316	CN_D310	CN_D298	CN_D251
4	FAM155A	CN_D302.2	CN_D296	CN_D253.2	CN_D251
4	FAM129A	CN_D296	CN_D288	CN_D252	CN_D224
4	FAIM3	CN_D294	CN_D292	CN_D288	CN_D252
4	FABP3	CN_D300	CN_D296	CN_D292	CN_D253.2
4	F13B	CN_D310	CN_D294	CN_D288	CN_D252
4	EYA3	CN_D316	CN_D306	CN_D253.2	CN_324
4	EXOSC4	CN_328	CN_D254	CN_D224	CN_330
4	EXOC2	CN_D292	CN_D288	CN_D252	CN_328
4	ETV7	CN_D292	CN_D288	CN_D252	CN_328
4	ETV3L	CN_D288	CN_D252	CN_D251	CN_D224
4	ETV3	CN_D288	CN_D252	CN_D251	CN_D224
4	ERCC5	CN_D318	CN_D302.2	CN_D296	CN_D251
4	ERC1	CN_D318	CN_D254	CN_D251	CN_324
4	EPRS	CN_D294	CN_D292	CN_D253.2	CN_D252
4	EPPK1	CN_328	CN_D254	CN_D224	CN_330
4	EPB41L4A	CN_D298	CN_D254	CN_D253.2	CN_328
4	ENY2	CN_D310	CN_D254	CN_D224	CN_328
4	ENSA	CN_D292	CN_D288	CN_D227	CN_D224
4	ENPP5	CN_D292	CN_D288	CN_D252	CN_328
4	ENPP1	CN_D298	CN_D296	CN_D288	CN_D253.2
4	ENAH	CN_D294	CN_D292	CN_D252	CN_320
4	ELOVL5	CN_D296	CN_D292	CN_D288	CN_D252
4	ELK4	CN_D300	CN_D288	CN_D252	CN_320

4	ELF3	CN_D300	CN_D292	CN_D252	CN_D224
4	EIF4G3	ZS.DNA26	CN_D296	CN_D253.2	CN_D251
4	EIF2C2	CN_D224	CN_328	CN_D254	CN_330
4	EID2B	CN_D316	CN_D310	CN_D298	CN_D251
4	EGFL8	CN_D292	CN_D288	CN_D252	CN_328
4	EFNB2	CN_D318	CN_D302.2	CN_D253.2	CN_D251
4	EFCAB2	CN_D300	CN_D292	CN_D252	CN_330
4	EDEM3	CN_D296	CN_D288	CN_D252	CN_D224
4	EBF1	CN_D298	CN_D254	CN_D253.2	CN_D224
4	EBAG9	CN_D254	CN_D224	CN_330	CN_328
4	DUSP22	CN_D292	CN_D288	CN_D252	CN_328
4	DTX3	CN_D310	CN_D298	CN_D296	CN_D254
4	DTNBP1	CN_D296	CN_D292	CN_D288	CN_D252
4	DROSHA	CN_D224	CN_D288	CN_D253.2	CN_D225
4	DPY19L4	CN_D254	CN_D224	CN_330	CN_328
4	DPT	CN_D294	CN_D292	CN_D252	CN_D224
4	DOCK9	CN_D302.2	CN_D296	CN_D253.2	CN_D251
4	DOCK4	CN_D225	CN_D316	CN_D253.2	CN_D224
4	DOCK1	CN_D296	CN_D294	CN_D288	CN_D253.2
4	DNM2	ZS.DNA11	CN_D316	CN_328	CN_324
4	DNAJC21	CN_D224	CN_D288	CN_D252	CN_D225
4	DLL3	CN_D316	CN_D310	CN_D298	CN_D251
4	DLK2	CN_D292	CN_D288	CN_D252	CN_328
4	DLGAP1	CN_D318	CN_D294	CN_D253.2	CN_D251
4	DKFZp686O132	CN_D318	CN_D292	CN_D224	CN_328
4	DIAPH3	CN_D302.2	CN_D298	CN_D296	CN_D251
4	DGKI	CN_D225	CN_D316	CN_D253.2	CN_D224
4	DEGS1	CN_D292	CN_D288	CN_D252	CN_320
4	DEFB112	CN_D296	CN_D292	CN_D288	CN_D252
4	DEFB110	CN_D296	CN_D292	CN_D288	CN_D252
4	DEF6	CN_D292	CN_D288	CN_D252	CN_328
4	DDX59	CN_D294	CN_D292	CN_D252	CN_320
4	DAP3	CN_D252	CN_D251	CN_D224	CN_320
4	DAB1	CN_D300	CN_D296	CN_D251	CN_D224
4	CYP27B1	CN_D310	CN_D298	CN_D296	CN_D254
4	CYCSP52	CN_D288	CN_D252	CN_D251	CN_D224
4	CYC1	CN_328	CN_D254	CN_D224	CN_330
4	CUTA	CN_D292	CN_D288	CN_D252	CN_328
4	CTSK	CN_D288	CN_D251	CN_D227	CN_D224
4	CTSE	CN_D310	CN_D300	CN_D292	CN_D252
4	CTGF	CN_D298	CN_D296	CN_D288	CN_D253.2
4	CTDSP2	CN_D310	CN_D298	CN_D296	CN_D254
4	CSF2	CN_D296	CN_D224	CN_328	CN_324

4	CSDE1	CN_D300	CN_D296	CN_D253.2	CN_D224
4	CRK	CN_D316	CN_D306	CN_328	CN_324
4	CRISP3	CN_D292	CN_D288	CN_D252	CN_328
4	CRISP2	CN_D292	CN_D288	CN_D252	CN_328
4	COPG2	CN_D225	CN_D253.2	CN_D225	CN_D224
4	COPA	CN_D294	CN_D292	CN_D251	CN_D224
4	COL4A2	CN_D302.2	CN_D296	CN_D253.2	CN_D251
4	COL4A1	CN_D302.2	CN_D296	CN_D253.2	CN_D251
4	COG2	CN_D310	CN_D292	CN_D288	CN_D252
4	CNTNAP4	CN_D306	CN_D302.2	CN_D254	CN_320
4	CNTN6	CN_D298	CN_D296	CN_D253.2	CN_D225
4	CNTN4	CN_D298	CN_D296	CN_D253.2	CN_D225
4	CNTN2	CN_D310	CN_D292	CN_D252	CN_320
4	CNOT4	CN_D316	CN_D253.2	CN_D225	CN_D224
4	CMBL	CN_D288	CN_D253.2	CN_D225	CN_328
4	CMAHP	CN_D292	CN_D288	CN_D253.2	CN_D252
4	CLPTM1L	CN_D225	CN_D302.2	CN_D296	CN_328
4	CLIC5	CN_D292	CN_D288	CN_D253.2	CN_D252
4	CIRH1A	CN_D296	CN_328	CN_324	CN_320
4	CHTOP	CN_D310	CN_D296	CN_D227	CN_328
4	CHTF8	CN_D296	CN_328	CN_324	CN_320
4	CHIT1	CN_D310	CN_D294	CN_D252	CN_D224
4	CENPL	CN_D294	CN_D292	CN_D252	CN_D224
4	CDYL	CN_D292	CN_D288	CN_D252	CN_328
4	CDSN	CN_D292	CN_D288	CN_D252	CN_328
4	CDK4	CN_D310	CN_D298	CN_D296	CN_D254
4	CDK14	CN_D225	CN_D316	CN_D253.2	CN_D224
4	CDC42SE1	CN_D294	CN_D251	CN_D227	CN_D224
4	CD83	CN_D292	CN_D288	CN_D252	CN_328
4	CD74	CN_D298	CN_D254	CN_D253.2	CN_328
4	CD55	CN_D310	CN_D300	CN_D288	CN_D252
4	CD48	CN_D300	CN_D294	CN_D251	CN_D224
4	CD2AP	CN_D292	CN_D288	CN_D253.2	CN_D252
4	CD1E	CN_D302.2	CN_D294	CN_D292	CN_D252
4	CD1C	CN_D292	CN_D252	CN_D251	CN_D224
4	CD1B	CN_D302.2	CN_D294	CN_D292	CN_D252
4	CD160	CN_D310	CN_D292	CN_D227	CN_D224
4	CCT3	CN_D292	CN_D252	CN_D251	CN_D224
4	CCND3	CN_D292	CN_D288	CN_D252	CN_328
4	CCDC90A	CN_D292	CN_D288	CN_D252	CN_328
4	CCDC168	CN_D318	CN_D302.2	CN_D296	CN_D251
4	CCDC167	CN_D292	CN_D288	CN_D252	CN_328
4	CCDC166	CN_328	CN_D318	CN_D254	CN_D224

4	CASQ1	CN_D310	CN_D292	CN_D251	CN_D224
4	CARD6	CN_D224	CN_D296	CN_D288	CN_D225
4	CAPSL	CN_D224	CN_D296	CN_D288	CN_D225
4	CAPS2	CN_D310	CN_D298	CN_D254	CN_D252
4	CAPN9	CN_D310	CN_D292	CN_D288	CN_D252
4	CAMTA1	CN_D300	CN_D296	CN_D253.2	CN_D251
4	CAMK1D	CN_D316	CN_D253.2	CN_330	CN_320
4	CACNA1E	CN_D296	CN_D288	CN_D252	CN_D224
4	CABLES2	CN_D318	CN_D298	CN_D296	CN_D251
4	CA7	CN_D298	CN_D251	CN_328	CN_320
4	CA5A	CN_D253.2	CN_328	CN_324	CN_320
4	CA14	CN_D300	CN_D292	CN_D227	CN_D224
4	C8orf80	CN_D298	CN_D253.2	CN_D251	CN_324
4	C8orf4	CN_D318	CN_D298	CN_D296	CN_D253.2
4	C8orf45	CN_328	CN_D254	CN_D224	CN_330
4	C8orf22	CN_328	CN_D318	CN_D288	CN_D254
4	C7	CN_D224	CN_D296	CN_D288	CN_D225
4	C6orf226	CN_D292	CN_D288	CN_D252	CN_328
4	C6orf1	CN_D292	CN_D288	CN_D252	CN_328
4	C6orf195	CN_D292	CN_D288	CN_D253.2	CN_D252
4	C6orf15	CN_D292	CN_D288	CN_D252	CN_328
4	C6orf132	CN_D292	CN_D288	CN_D252	CN_328
4	C6orf130	CN_D292	CN_D288	CN_D253.2	CN_D252
4	C6orf106	CN_D292	CN_D288	CN_D252	CN_328
4	C6	CN_D224	CN_D296	CN_D288	CN_D225
4	C5orf56	CN_D254	CN_D253.2	CN_328	CN_324
4	C5orf30	CN_D298	CN_D296	CN_D253.2	CN_D224
4	C4BPB	CN_D294	CN_D288	CN_D253.2	CN_D252
4	C4BPA	CN_D294	CN_D288	CN_D253.2	CN_D252
4	C20orf151	CN_D318	CN_D298	CN_D296	CN_D251
4	C1QTNF3-AMA	CN_D224	CN_D298	CN_D288	CN_D225
4	C1orf9	CN_D288	CN_D252	CN_D224	CN_320
4	C1orf85	CN_D292	CN_D252	CN_D251	CN_D224
4	C1orf68	CN_D292	CN_D251	CN_328	CN_320
4	C1orf56	CN_D294	CN_D251	CN_D227	CN_D224
4	C1orf54	CN_D300	CN_D292	CN_D227	CN_D224
4	C1orf51	CN_D300	CN_D292	CN_D227	CN_D224
4	C1orf220	CN_D310	CN_D292	CN_D252	CN_D224
4	C1orf186	CN_D310	CN_D300	CN_D292	CN_D252
4	C1orf116	CN_D294	CN_D288	CN_D253.2	CN_D252
4	C1orf112	CN_D302.2	CN_D292	CN_D252	CN_D224
4	C15orf38-AP3S2	CN_D318	CN_D300	CN_D253.2	CN_324
4	C12orf53	CN_D300	CN_D298	CN_328	CN_324

4	C12orf4	CN_D318	CN_D298	CN_D254	CN_324
4	BYSL	CN_D292	CN_D288	CN_D252	CN_328
4	BROX	CN_D294	CN_D292	CN_D288	CN_D252
4	BRIX1	CN_D224	CN_D288	CN_D252	CN_D225
4	BREA2	CN_328	CN_D318	CN_D254	CN_D224
4	BPHL	CN_D292	CN_D288	CN_D252	CN_328
4	BNIPL	CN_D294	CN_D251	CN_D227	CN_D224
4	BIVM-ERCC5	CN_D318	CN_D302.2	CN_D296	CN_D251
4	BIVM	CN_D318	CN_D302.2	CN_D296	CN_D251
4	BHLHA9	ZS.DNA26	CN_D306	CN_328	CN_324
4	BGLAP	CN_D292	CN_D252	CN_D251	CN_D224
4	B4GALNT1	CN_D310	CN_D298	CN_D296	CN_D254
4	B3GAT1	CN_D298	CN_D254	CN_D253.2	CN_D251
4	AVIL	CN_D310	CN_D298	CN_D296	CN_D254
4	ATP6V1G3	CN_D300	CN_D294	CN_D292	CN_D252
4	ATP6V0A4	CN_D225	CN_D316	CN_D253.2	CN_D224
4	ATP1A4	CN_D310	CN_D292	CN_D251	CN_D224
4	ATP1A2	CN_D310	CN_D292	CN_D251	CN_D224
4	ATF6B	CN_D292	CN_D288	CN_D252	CN_328
4	ASPM	CN_D310	CN_D294	CN_D288	CN_D252
4	ARPC5	CN_D302.2	CN_D253.2	CN_D252	CN_D224
4	ARMC2	CN_D298	CN_D296	CN_D288	CN_D253.2
4	ARHGEF25	CN_D310	CN_D298	CN_D296	CN_D254
4	ARF1	CN_D300	CN_D292	CN_D252	CN_320
4	ARC	CN_328	CN_D318	CN_D254	CN_D224
4	APOBEC4	CN_D302.2	CN_D253.2	CN_D252	CN_D224
4	APOBEC2	CN_D292	CN_D288	CN_D253.2	CN_D252
4	APH1A	CN_D300	CN_D292	CN_D227	CN_D224
4	AP3S2	CN_D318	CN_D300	CN_D253.2	CN_324
4	ANP32E	CN_D300	CN_D292	CN_D227	CN_D224
4	ANKS1B	CN_D298	CN_D288	CN_D254	CN_D253.2
4	ANKS1A	CN_D292	CN_D288	CN_D252	CN_328
4	ANKRD45	CN_D294	CN_D292	CN_D252	CN_D224
4	ANKRD35	CN_D292	CN_D253.2	CN_D227	CN_D224
4	ANK3	CN_D296	CN_D253.2	CN_D251	CN_320
4	ALDH5A1	CN_D292	CN_D288	CN_D252	CN_328
4	AIDA	CN_D294	CN_D292	CN_D288	CN_D252
4	AHCTF1	CN_D302.2	CN_D296	CN_D292	CN_D252
4	AGXT2	CN_D224	CN_D288	CN_D252	CN_D225
4	AGT	CN_D310	CN_D292	CN_D288	CN_D252
4	AGSK1	CN_D318	CN_D304	CN_D300	CN_D253.2
4	AGPAT1	CN_D292	CN_D288	CN_D252	CN_328
4	AGER	CN_D292	CN_D288	CN_D252	CN_328

4	AGBL1	CN_D318	CN_D300	CN_330	CN_324
4	AGAP2	CN_D310	CN_D298	CN_D296	CN_D254
4	ADTRP	CN_D296	CN_D292	CN_D288	CN_D252
4	ADSS	CN_D310	CN_D300	CN_D252	CN_320
4	ADRA1B	CN_D316	CN_D298	CN_D254	CN_D253.2
4	ADARB2	ZS.DNA9	CN_D253.2	CN_D224	CN_320
4	ADAMTSL1	CN_D296	CN_D251	CN_D224	CN_324
4	ADAMTS6	CN_D298	CN_D296	CN_D253.2	CN_D224
4	ADAM5P	CN_D318	CN_D296	CN_D253.2	CN_320
4	ACTA1	CN_D310	CN_D292	CN_D252	CN_328
4	ACBD3	CN_D294	CN_D292	CN_D252	CN_328
4	ABRA	CN_D254	CN_D224	CN_330	CN_328
4	ABHD2	CN_D318	CN_D300	CN_D253.2	CN_324
4	ABCC4	CN_D302.2	CN_D296	CN_D253.2	CN_D251
4	ABCC10	CN_D292	CN_D288	CN_D252	CN_328
4	ABCB10	CN_D310	CN_D292	CN_D252	CN_328
4	A2LD1	CN_D318	CN_D302.2	CN_D296	CN_D251
4	A1CF	CN_D296	CN_D288	CN_D253.2	CN_D224

Supplementary Table 4: Full list of 138 overlapped genes following integration analysis of both datasets

Gene Symbol	Chromosome	CNV Status	Gene Expression Status	Expression adjusted *p* -value	Expression log2FoldChange	Total Number of CNV Samples	Total Duplications/Deletions
ACBD3	1	Duplications	Up-regulated	5.19725E-11	1.015333312	33	4
ADAR	1	Duplications	Up-regulated	3.05531E-07	1.001635197	33	5
ADSS	1	Duplications	Up-regulated	1.77142E-08	1.135201241	33	4
AIDA	1	Duplications	Up-regulated	1.45919E-05	1.029605764	33	4
ANP32E	1	Duplications	Up-regulated	5.60981E-09	1.096149188	33	4
ARID4B	1	Duplications	Up-regulated	6.6997E-11	1.097242837	33	7
ARMC1	8	Duplications	Up-regulated	8.51789E-08	1.186415755	33	7
ARPC5	1	Duplications	Up-regulated	1.23783E-07	1.068340654	33	4
ASPM	1	Duplications	Up-regulated	1.04534E-17	3.367553875	33	4
ATAD2	8	Duplications	Up-regulated	6.91321E-13	1.891988491	33	5
ATP6V1C1	8	Duplications	Up-regulated	2.61883E-09	1.011276926	33	6
B4GALT3	1	Duplications	Up-regulated	8.19703E-08	1.01449472	33	6
C6orf48	6	Duplications	Up-regulated	2.65864E-07	1.211013113	33	5
CACYBP	1	Duplications	Up-regulated	1.19404E-09	1.050002019	33	6
CAP2	6	Duplications	Up-regulated	1.52322E-16	2.491638731	33	6
CCT3	1	Duplications	Up-regulated	7.03867E-19	1.815390436	33	4
CDK4	12	Duplications	Up-regulated	1.03685E-08	1.282058376	33	4
CENPF	1	Duplications	Up-regulated	7.14296E-11	1.06993333	33	7
CLIC1	6	Duplications	Up-regulated	1.10412E-08	1.351323029	33	6
CNIH4	1	Duplications	Up-regulated	2.20092E-13	1.986601668	33	5
COG2	1	Duplications	Up-regulated	8.32169E-11	1.134883139	33	4
COL4A1	13	Duplications	Up-regulated	2.19093E-09	1.784282051	33	4
DAP3	1	Duplications	Up-regulated	3.82722E-12	1.410621511	33	4
DTL	1	Duplications	Up-regulated	2.62845E-13	2.252950236	33	9
EIF3E	8	Duplications	Up-regulated	7.51137E-15	1.048527713	33	6
EIF3H	8	Duplications	Up-regulated	7.77337E-10	1.148135259	33	6
ENAH	1	Duplications	Up-regulated	6.55246E-14	2.108118095	33	4
ENPP2	8	Duplications	Up-regulated	0.000267407	1.250674423	33	7
EPRS	1	Duplications	Up-regulated	1.24017E-11	1.146792964	33	4
FZD6	8	Duplications	Up-regulated	1.24727E-06	1.098197638	33	7
GGPS1	1	Duplications	Up-regulated	1.18356E-11	1.12455013	33	6
GMNN	6	Duplications	Up-regulated	1.33128E-12	2.349779838	33	4
GOLPH3L	1	Duplications	Up-regulated	7.14681E-08	1.346170521	33	5
HEATR1	1	Duplications	Up-regulated	7.4628E-16	1.628983935	33	8
HMGA2	12	Duplications	Up-regulated	0.01518059	1.072957533	33	4
HSP90AB1	6	Duplications	Up-regulated	1.02655E-09	1.544227397	33	4
ILF2	1	Duplications	Up-regulated	8.24083E-15	1.96701415	33	4
INTS7	1	Duplications	Up-regulated	7.59344E-10	1.039422795	33	9
INTS8	8	Duplications	Up-regulated	2.6598E-10	1.301380737	33	6
KIAA0196	8	Duplications	Up-regulated	2.65328E-06	1.027417037	33	7
KIF14	1	Duplications	Up-regulated	1.9182E-08	1.05977456	33	5
LAMC1	1	Duplications	Up-regulated	1.18718E-16	1.763236242	33	8
LAPTM4B	8	Duplications	Up-regulated	5.08607E-10	2.171776317	33	6
LBR	1	Duplications	Up-regulated	1.14157E-12	1.56008928	33	4

LPGAT1	1	Duplications	Up-regulated	1.0792E-10	1.838599702	33	5
MCM3	6	Duplications	Up-regulated	5.04374E-10	1.396072937	33	4
MEP1A	6	Duplications	Up-regulated	0.001013466	1.0357586	33	4
MRPL13	8	Duplications	Up-regulated	1.11905E-10	1.186405352	33	7
MTR	1	Duplications	Up-regulated	2.21687E-08	1.305989566	33	6
NDRG1	8	Duplications	Up-regulated	5.59364E-05	1.092679103	33	6
NEK2	1	Duplications	Up-regulated	6.48297E-11	1.068276358	33	4
NEU1	6	Duplications	Up-regulated	5.85057E-10	1.591728219	33	5
NPM1	5	Duplications	Up-regulated	6.63136E-14	1.216657938	33	4
NUP133	1	Duplications	Up-regulated	1.69253E-14	1.524039529	33	4
NVL	1	Duplications	Up-regulated	4.69803E-10	1.035153207	33	5
P4HA2	5	Duplications	Up-regulated	5.24475E-08	1.490031261	33	5
PABPC1	8	Duplications	Up-regulated	3.45398E-14	1.055000425	33	8
PEA15	1	Duplications	Up-regulated	2.11508E-12	1.314348466	33	4
POLR2K	8	Duplications	Up-regulated	1.85171E-11	1.005782163	33	8
PRCC	1	Duplications	Up-regulated	5.41265E-13	1.357751933	33	9
PRIM1	12	Duplications	Up-regulated	2.94762E-09	1.419182684	33	4
RAD21	8	Duplications	Up-regulated	4.77126E-11	1.257372767	33	6
RGS5	1	Duplications	Up-regulated	9.16335E-07	1.479597871	33	4
RNF187	1	Duplications	Up-regulated	7.56477E-09	1.135227304	33	5
RPL8	8	Duplications	Up-regulated	9.0357E-12	1.098502999	33	6
RPS6KC1	1	Duplications	Up-regulated	6.63136E-14	1.422173834	33	7
RRP15	1	Duplications	Up-regulated	1.97034E-15	1.866463489	33	5
RRS1	1	Duplications	Up-regulated	2.24674E-08	1.27362958	33	5
SCRIB	8	Duplications	Up-regulated	3.56729E-06	1.038538473	33	4
SERPINB1	6	Duplications	Up-regulated	1.20476E-11	1.449968522	33	6
SF3B4	1	Duplications	Up-regulated	5.5408E-15	1.416537044	33	5
SHC1	1	Duplications	Up-regulated	4.90936E-11	1.214273709	33	4
SMYD3	1	Duplications	Up-regulated	1.41286E-09	1.622141333	33	8
SOX4	6	Duplications	Up-regulated	1.29675E-07	1.164555504	33	5
SQLE	8	Duplications	Up-regulated	2.01898E-10	1.918933758	33	6
SSR1	6	Duplications	Up-regulated	1.02392E-09	1.010241693	33	5
SSR2	1	Duplications	Up-regulated	1.46431E-14	1.591435906	33	6
TARBP1	1	Duplications	Up-regulated	1.35668E-07	1.359373346	33	4
TBCE	1	Duplications	Up-regulated	7.99683E-13	1.704009057	33	7
TOMM20	1	Duplications	Up-regulated	1.84301E-08	1.278939264	33	5
TP53BP2	1	Duplications	Up-regulated	4.36223E-12	1.614068398	33	4
TTC13	1	Duplications	Up-regulated	7.45168E-12	1.574987498	33	4
TUBB	6	Duplications	Up-regulated	1.06846E-07	1.253520071	33	4
TUBB2A	6	Duplications	Up-regulated	6.32964E-06	1.284578325	33	4
UBD	6	Duplications	Up-regulated	7.56477E-09	2.747886046	33	4
UBE2Q1	1	Duplications	Up-regulated	2.28481E-12	1.278338376	33	5
UCHL5	1	Duplications	Up-regulated	2.15601E-10	1.015561686	33	7
UCK2	1	Duplications	Up-regulated	3.70541E-11	1.486016609	33	6
UFC1	1	Duplications	Up-regulated	3.61618E-10	1.050496177	33	5
WDYHV1	8	Duplications	Up-regulated	1.0792E-10	1.374535497	33	4

YWHAZ	8	Duplications	Up-regulated	3.03975E-09	1.504428643	33	9
YY1AP1	1	Duplications	Up-regulated	4.3478E-05	1.027145752	33	8
ZNF706	8	Duplications	Up-regulated	8.2716E-09	1.083608975	33	6
ABCG2	4	Deletions	Down-regulated	3.205E-05	-1.577090151	33	5
ADH1B	4	Deletions	Down-regulated	1.15015E-06	-2.55318541	33	4
ADH1C	4	Deletions	Down-regulated	7.97758E-10	-3.37959439	33	4
ADH6	4	Deletions	Down-regulated	2.20982E-11	-2.23257594	33	4
ADK	10	Deletions	Down-regulated	6.73621E-08	-1.199639622	33	4
AKR7A3	1	Deletions	Down-regulated	1.08103E-09	-2.228795953	33	4
ANXA10	4	Deletions	Down-regulated	1.98662E-13	-2.859657319	33	5
CBR4	4	Deletions	Down-regulated	1.02255E-15	-1.820031068	33	5
CFI	4	Deletions	Down-regulated	1.75143E-07	-1.422497052	33	4
CTSO	4	Deletions	Down-regulated	2.83141E-06	-1.490523521	33	4
CUX2	12	Deletions	Down-regulated	3.36881E-06	-1.953247094	33	4
CXCL2	4	Deletions	Down-regulated	1.35392E-08	-2.590694017	33	4
ENPEP	4	Deletions	Down-regulated	4.98828E-08	-1.41979484	33	4
EPB41L4B	9	Deletions	Down-regulated	3.8999E-13	-1.470491725	33	5
EPHX2	8	Deletions	Down-regulated	2.73525E-11	-2.213837852	33	4
ETFDH	4	Deletions	Down-regulated	1.3363E-10	-1.996228523	33	4
F11	4	Deletions	Down-regulated	1.79028E-16	-2.493657749	33	5
FAM13A	4	Deletions	Down-regulated	2.73177E-17	-2.64866903	33	4
FAM149A	4	Deletions	Down-regulated	2.53276E-17	-2.076668936	33	5
FANCC	9	Deletions	Down-regulated	8.46969E-14	-1.380770952	33	4
GABARAPL1	12	Deletions	Down-regulated	6.49534E-11	-1.599009799	33	4
GPM6A	4	Deletions	Down-regulated	1.33394E-13	-1.125053298	33	5
HADH	4	Deletions	Down-regulated	5.7825E-10	-1.192164915	33	4
HP	16	Deletions	Down-regulated	1.0887E-06	-1.155205829	33	4
HPR	16	Deletions	Down-regulated	1.63345E-05	-1.649449401	33	4
KIAA0922	4	Deletions	Down-regulated	6.6997E-11	-1.59050454	33	5
KLHL2	4	Deletions	Down-regulated	1.96201E-12	-1.296621375	33	4
KLKB1	4	Deletions	Down-regulated	2.9606E-17	-2.991960946	33	5
LPA	6	Deletions	Down-regulated	1.26306E-19	-3.283355412	33	4
MAN1C1	1	Deletions	Down-regulated	1.91886E-13	-2.014221798	33	4
MPDZ	9	Deletions	Down-regulated	5.44066E-08	-1.575808707	33	4
MSRA	8	Deletions	Down-regulated	5.85057E-10	-1.729221579	33	4
MTTP	4	Deletions	Down-regulated	2.72551E-07	-1.944409978	33	4
NAT1	8	Deletions	Down-regulated	2.35465E-10	-1.561523709	33	4
NAT2	8	Deletions	Down-regulated	1.04534E-17	-3.972639429	33	4
NPY1R	4	Deletions	Down-regulated	1.02255E-15	-1.452671246	33	4
NR3C2	4	Deletions	Down-regulated	2.61967E-11	-1.189591741	33	4
PIK3C2G	12	Deletions	Down-regulated	2.94762E-09	-1.36497582	33	6
PLAC8	4	Deletions	Down-regulated	4.6421E-11	-2.026119829	33	4
PPP1R1A	12	Deletions	Down-regulated	6.11064E-05	-1.542331273	33	4
PSD3	8	Deletions	Down-regulated	3.13205E-08	-1.622003797	33	5
SLCO1B1	12	Deletions	Down-regulated	4.47005E-06	-1.736287588	33	6
SORBS2	4	Deletions	Down-regulated	3.61929E-09	-1.498277802	33	5

TDO2	4	Deletions	Down-regulated	6.71455E-06	-2.423017937	33	4
ZBTB16	11	Deletions	Down-regulated	9.82968E-06	-1.462011743	33	4

Supplementary Table 5: Functional and pathway enrichment analysis of CNV-driven DEGs in module

Category	Term/gene function	Count	*P* value
GOTERM_BP_DIRECT	GO:0006413~translational initiation	4	5.31E-07
GOTERM_BP_DIRECT	GO:0000184~nuclear-transcribed mRNA catabolic process, nonsense-mediated decay	3	1.49E-04
GOTERM_BP_DIRECT	GO:0001731~formation of translation preinitiation complex	2	0.0041037
GOTERM_BP_DIRECT	GO:0006446~regulation of translational initiation	2	0.0064182
GOTERM_CC_DIRECT	GO:0016020~membrane	4	0.001757
GOTERM_CC_DIRECT	GO:0033290~eukaryotic 48S preinitiation complex	2	0.002467
GOTERM_CC_DIRECT	GO:0016282~eukaryotic 43S preinitiation complex	2	0.002467
GOTERM_CC_DIRECT	GO:0005852~eukaryotic translation initiation factor 3 complex	2	0.002796
GOTERM_CC_DIRECT	GO:0005829~cytosol	4	0.006014
GOTERM_MF_DIRECT	GO:0044822~poly(A) RNA binding	4	2.98E-04
GOTERM_MF_DIRECT	GO:0003743~translation initiation factor activity	2	0.010802
KEGG PATHWAY	hsa03013: RNA transport	3	3.34E-07
KEGG PATHWAY	hsa03018: RNA degradation	1	0.0078235
KEGG PATHWAY	hsa03015: mRNA surveillance pathway	1	0.0093228
KEGG PATHWAY	hsa05160: Hepatitis C	1	0.013412
KEGG PATHWAY	hsa03010: Ribosome	1	0.01391

If there were more than five terms enriched in this category, top five terms were selected according to *P* value.

Count: the number of enriched genes in each term

DEGs, differentially expressed genes; GO, gene ontology; KEGG, Kyoto Encyclopedia of Genes and Genomes; CNV, copy number variation
